# Proline-promoted dehydroxylation of α-ketols†
†Electronic supplementary information (ESI) available. See DOI: 10.1039/c9sc02543j


**DOI:** 10.1039/c9sc02543j

**Published:** 2019-08-19

**Authors:** Yelena Mostinski, David Lankri, Yana Konovalov, Riva Nataf, Dmitry Tsvelikhovsky

**Affiliations:** a The Institute for Drug Research , Faculty of Medicine , The Hebrew University of Jerusalem , Jerusalem 9112102 , Israel . Email: dmitryt@ekmd.huji.ac.il

## Abstract

A new single-step proline-potassium acetate promoted reductive dehydroxylation of α-ketols is reported. We introduce the unexplored reactivity of proline and, for the first time, reveal its ability to function as a reducing agent. The developed metal-free and open-flask operation generally results in good yields. Our protocol allows the challenging selective dehydroxylation of hydroxyketones without affecting other functional groups.

## Abstract 2

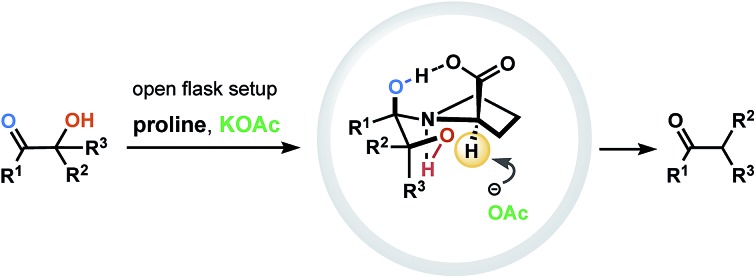
A new single-step proline-potassium acetate promoted reductive dehydroxylation of α-ketols is reported. We introduce the unexplored reactivity of proline and, for the first time, reveal its ability to function as a reducing agent.

## Introduction

**Fig. 1:**
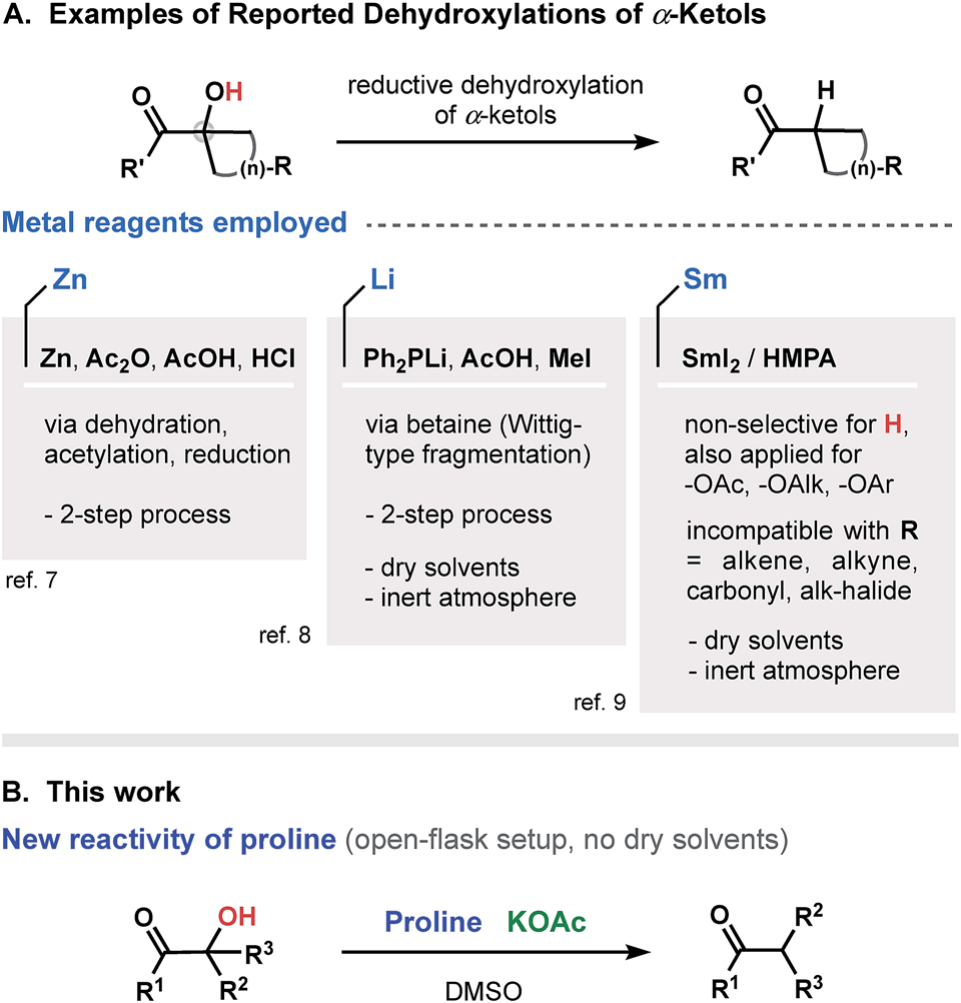
Reported and projected dehydroxylations of α-ketols.

Over the past few decades, tremendous progress has been made in the development of protocols for molecular functionalization, revolutionizing the logic of chemical synthesis and opening new ways to prepare complex molecular structures (*e.g.*, natural products, biologically important compounds, medicines and drug candidates). Among various activation/derivatization strategies, the carbon–halogenation (most commonly C–F bond formations),^1^ and C–H functionalization (borylation, trifluoromethylation, amidation, oxidation, and others),^2^ can be noted as the most prominent. A dehydroxylation through the reduction of α-ketols is another emerging method for a functionalization/derivatization, facilitating access to various molecular frameworks that are not otherwise readily accessible. The critical challenge of such transformation is to secure an appropriate reducing reagent that, on one hand, will not hinder the functionality of the catalyst–substrate complex and that, on the other hand, will activate the α-ketol despite its steric complexity. Several recognized approaches have been established during the past few decades (Fig. 1A covers the most prominent ones reported to date).^3^–^6^ Most employ metal reagents such as Zn,^7^ Li,^8^ and Sm.^9^ Nevertheless, as versatile as they may be, all of the modern strategies suffer from common disadvantages, such as multi-step operations, requirement of inert atmosphere and dry solvents, utilization of organometallic reagents, undesired dehydration, reduction or rearrangements,10*a* protection of hydroxyl groups10*b* and, finally, distortion or damage to other (proximal) functional groups integrated within the molecular scaffold.

In this study, we report on a new single-step selective reductive dehydroxylation process based on the interaction of α-ketols with proline in the presence of KOAc (Fig. 1B). We introduce the unexplored reactivity of proline and demonstrate its ability to function as a reducing agent. The developed metal-free and open-flask operation generally results in good yields and minimal formation of side products.

## Results and discussion

### Conditions evaluation

**Fig. 2:**
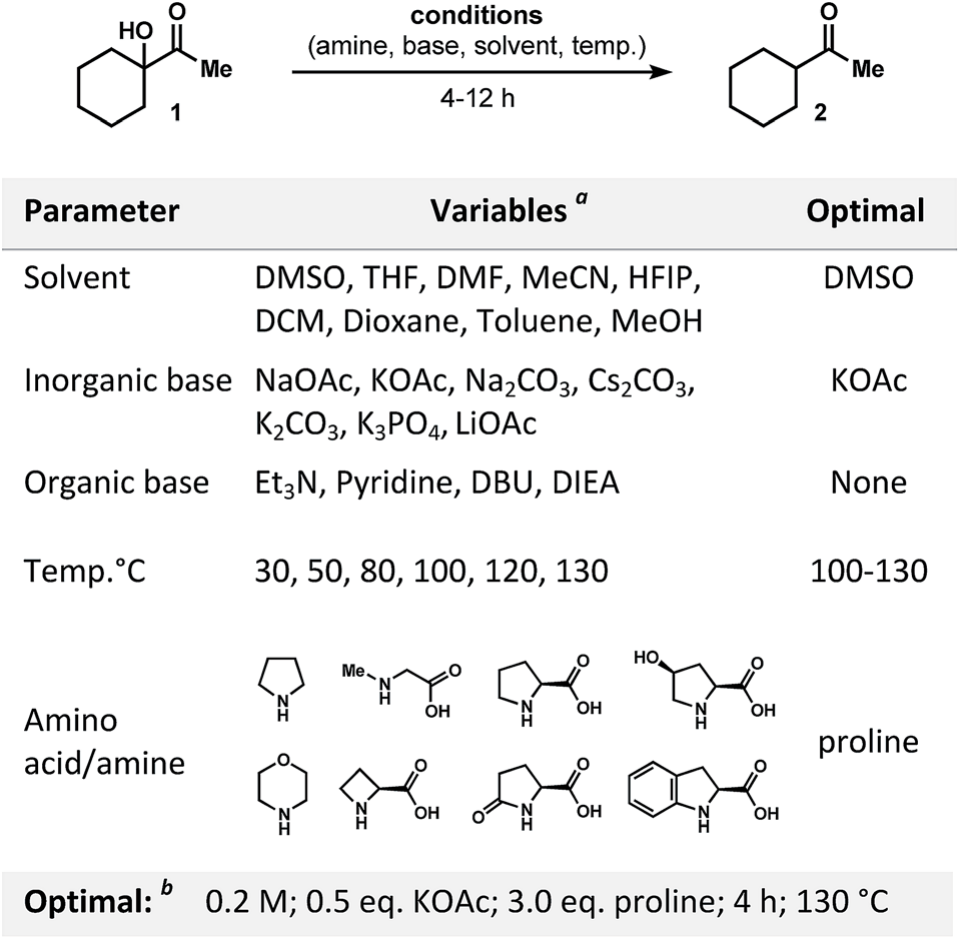
Optimization of proline-promoted dehydroxylation of α-ketols. ^*a*^ See ESI section;† ^*b* 1^H-NMR yield for optimal set 98%.

Our interest in this project began when, as a result of the reaction of α-ketol **1** with proline under basic conditions, the reduced product **2** was obtained (Fig. 2). Intriguingly, after reviewing the available literature, we did not find any reports (or even a mention) of general protocols for dehydroxylation of α-ketols using proline (or any kind of enamine/iminium proline-derived analogues). Our assumption was that four major factors would control the course of transformation: base, amino acid, solvent and temperature. We examined these variables (Fig. 2) and discovered that the mode of reduction is almost exclusively controlled by the amino acid employed. We tested a variety of amines and amino acids and found that proline was the only effective promoter for the reduction of **1** to form **2**. Apart from a rate dependence on the nature of amino acid, we found that the process is influenced by the nature of base (see Fig. 2 for bases examined). We also noticed that dehydroxylation was accompanied by a side-condensation of proline, resulted in the formation of pyrrolopyrazinedione.^11^ For this reason, the use of a superstoichiometric amount of proline was required (Fig. 2).

Among the solvents screened, the use of DMSO was superior in terms of solubility, yield, and conversion of the starting ketol. An efficient system for the desired reductive dehydroxylation was finally formed from the combination of proline (3 equiv.) and KOAc (0.5 equiv.) at 130 °C and concentration of 0.2 M. It should be noted that no other side products were formed under the optimized conditions. Other combinations of solvent, base, and various additives (phase transfer catalysts, surfactants, or organometallic reagents; see Tables 1 and 2 of the ESI section†) resulted in low yields (if any) of the desired product.^12^

**Fig. 3:**
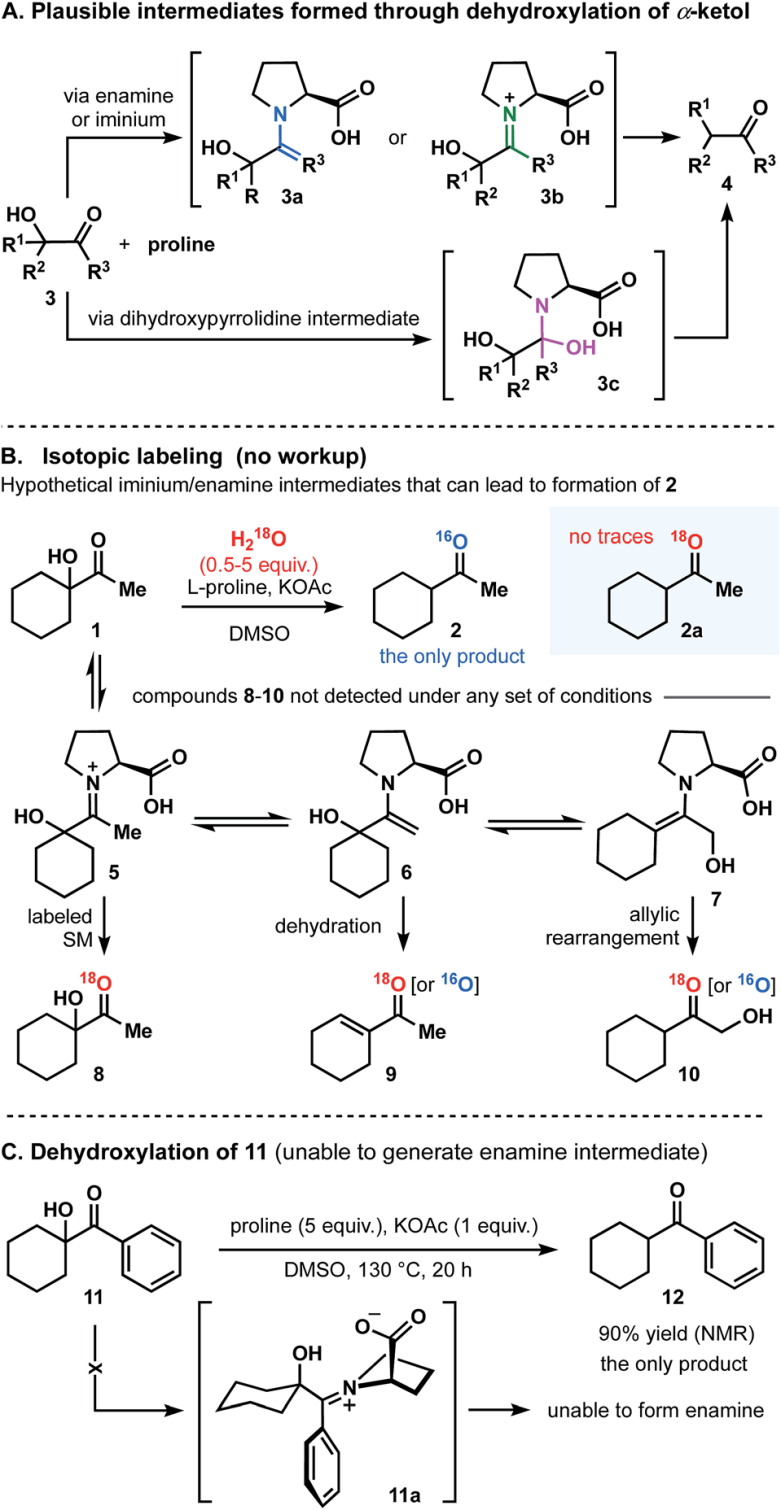
Disqualification of enamine and iminium pathways.

As we contemplated a possible mechanism to deliver the dehydroxylated product, a classical condensation of α-ketol **1** with l-proline first came to mind.^13^ It could have been assumed that the transformation in question is a two-step process in which enamine (**3a**), iminium (**3b**) or dihydroxypyrrolidine (**3c**) intermediates are formed (Fig. 3A).^14^–^18^ Thus, by analogy to previous studies,^13^–^18^ one would expect the intermediate to dehydrate (regardless of the pathway) and to then be further hydrolyzed, generating a ketone. But in such case, the role of a base (KOAc) in this process remains incomprehensible. Control experiments were performed and demonstrated that no reactions occurred in the absence of base. Below, we describe a series of experiments that helped us to understand and explain the proposed mechanism of this transformation.

### Disqualification of the enamine/iminium mechanism

In principle, the reaction of **3** with proline could produce intermediates **3a** and **3b** that could give rise to **4**.^13^ In order to differentiate among the pathways, the reaction of α-ketol **1** was carried out under the optimized conditions but in the presence of dry DMSO and molecular sieves. Compound **2** was then found in a crude reaction mixture (with no workup) as the only observable product, and the structure was assigned on the basis of its NMR spectra and the results of GCMS analysis. No evidence for the presence of starting ketol was detected. It should also be noted that no dehydrated outcome, rearrangement, or any other side-products were discovered in the reaction mixture.

In general, if the process was to occur through the enamine intermediate, water (released during the condensation) would have been necessary for the hydrolysis to obtain the product. In this experiment, the use of molecular sieves would have prevented this process from occurring. Since reduced product **2** is nevertheless obtained, we proposed that the enamine intermediate fails to take part in this reaction. In addition, it can be assumed that if dehydroxylation of **1** had progressed through iminium intermediate, the only available source of oxygen atom in the product molecule would be water. We thus carried out isotope labeling experiments introducing H_2_^18^O into reaction mixture (Fig. 3B).^17^ The ^18^O-labeled water was added at the start of the reaction course, at different rates, and in different quantities (0.5–5 equivalents range). As a result, no evidence was found for the incorporation of ^18^O into either the desired product (**2a**), or the starting material (**8**). We would also like to highlight that no additional ^18^O-labeled compounds (products or intermediates; Fig. 3B) **5-7**, or **9-10** were detected, thus proving the water to be a non-essential component in this reaction.

Finally, to disqualify the enamine pathway, we conducted an experiment replacing the aliphatic α-ketol **1** by an aromatic analogue **11**, which was ultimately unable to generate enamine (Fig. 3C). Thus, when the reaction was conducted under the optimized set of conditions, 90% reduced cyclohexylmethyl-methanone **12** was obtained (NMR and GC analysis). In light of this experiment and those described above, we concluded that the transformation of **1** to **2** does not follow the enamine pathway.

### The bi-bridged dihydroxypyrrolidinium as a key-intermediate in dehydroxylation pathway

Since KOAc proved to be an essential component in the proline-promoted dehydroxylation of α-ketols, its involvement in the transformation was investigated. Here, we propose a mechanism which allows us to determine the role of the base in this process.

**Fig. 4:**
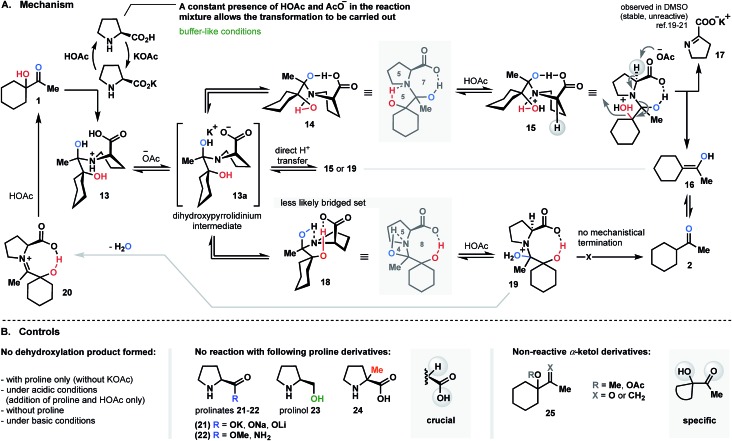
Proline-supported dehydroxylation (proposed mechanism).

We believe that the reduction process is likely initiated by generation of an acetate buffer upon the introduction of KOAc into the mixture of proline and α-ketol **1** (the stage where the first equivalent of proline is consumed; Fig. 4A). Only a constant presence of resulting acetic acid and acetate ions would allow for the transformation to take place. It should be mentioned that all control experiments, such as direct addition of AcOH (with no KOAc), reaction without base, or introduction of prepared-in-advance potassium-prolinate, had no effect, and no product was detected. Other metal-prolinate pairs (**21**; Fig. 4B) also failed to promote the reaction course. In all cases, the starting material was recovered. Next, condensation of ketol with proline is conducted to yield **13**. Further deprotonation of **13** with formed acetate base (^–^OAc) affords key dihydroxy-pyrrolidinium intermediate **13a**. We assumed that slow and direct intramolecular hydrogen transfer towards compounds **15** or **19** also might occur, but this route is less likely due to the presence of base. Therefore, the deprotonation pathway is more reasonable. Next, the folding of **13a** occurs to produce angularly fused transient intermediates **14** or **18** as a result of intramolecular hydrogen mediation. It is obvious that further dehydroxylation of intermediate **13a** must be associated with such bridging.^14^–^18^ To strengthen our hypothesis, we conducted a series of experiments replacing the acid moiety of proline by ester, amide or hydroxyl groups (**22** and **23**; Fig. 4B). Since none of the efforts mentioned yielded a reduced product, it can be concluded that “the acid” functionality is crucial in the reductive pathway. Considering the two intermediates **14** and **18**, we assume that structure **14** would be the most likely topological set, forming a stable tricyclic spiranoid 5/7/5 ring system. The intermediate **18**, on the other hand, would be arranged into a precarious frame of unstable 5/8/4 combination with no further mechanistic termination to release the product (see Fig. 4A).

The decisive step of the dehydroxylation circuit is an α-deprotonation of **15** (resulting after protonation of **14**) under acetate base conditions to liberate enol **16** (as shown in Fig. 4A) and the potassium salt of pyrroline-2-carboxylate **17** (observed in DMSO as salt; shows diminished tendency to decompose or react,^19^ but could be reduced and recovered as proline).^20^,^21^ To the best of our understanding, this is the only logical termination of the reductive dehydroxylation cycle. To verify this hypothesis, the α-methylated proline – compound **24** (Fig. 4B) was selected as a control promoter and subjected to the optimal conditions. No reaction was detected in this case, and starting ketol **1** was recovered. This result is consistent with our assumption and indicates the feasibility of the proposed dehydroxylation cycle.

The theory of a bi-bridged spiranoid intermediate was also investigated by performing structural modifications of the ketol counterpart (this time, the proline structure remained unchanged). We employed alternative starting materials comprised of substituted hydroxyl and carbonyl groups. Compounds **25** (Fig. 4B) were synthesized and subjected to optimized conditions. As in previous experiments, the examination of the reaction mixture failed to reveal the presence of any product.

**Fig. 5:**
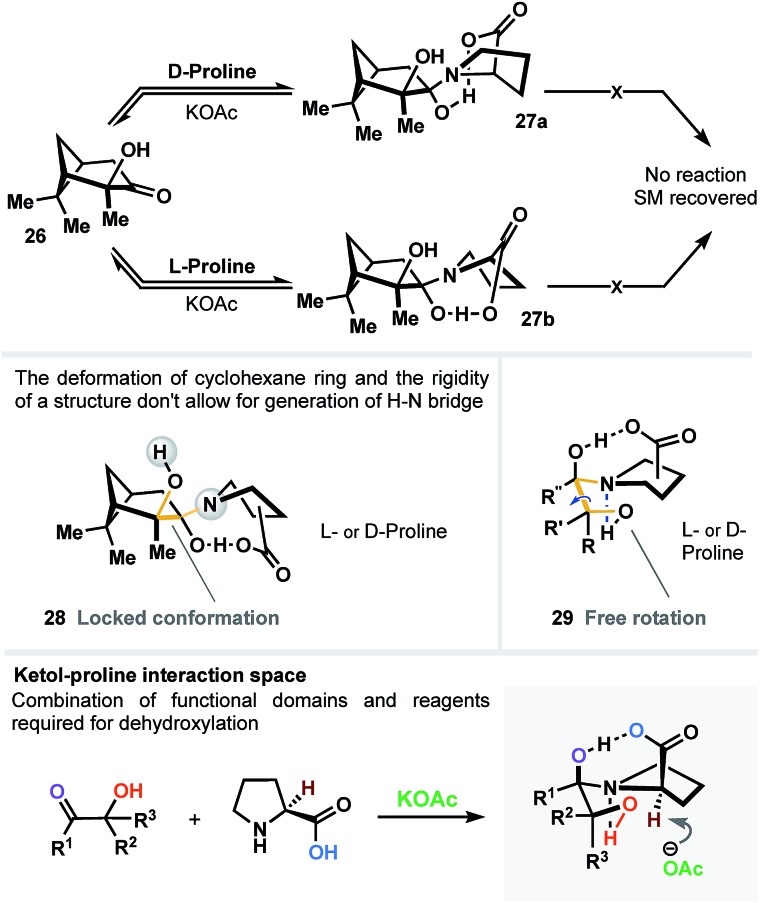
Rigid geometry of α-hydroxyketone domain inhibits formation of the bi-bridged intermediate, vital to initiate the dehydroxylation process.

In another scenario, chiral hydroxypinanone **26** was subjected to dehydroxylation conditions (Fig. 5) in the presence of l- and d-proline, separately. As a result (regardless the proline employed), we detected no sign of reaction. We believe that such behavior is directly attributable to the significant geometrical deformation of cyclohexane ring (**28**) and the rigidity of the structure (in contrast to unlocked topology **29**) – both don't allow for generation of a vital H–N bridge, narrowing the interaction space of the carboxylic acid, α-OH group and the ketone, all simultaneously, as three critical domains (Fig. 5).

### Kinetic resolution

**Fig. 6:**
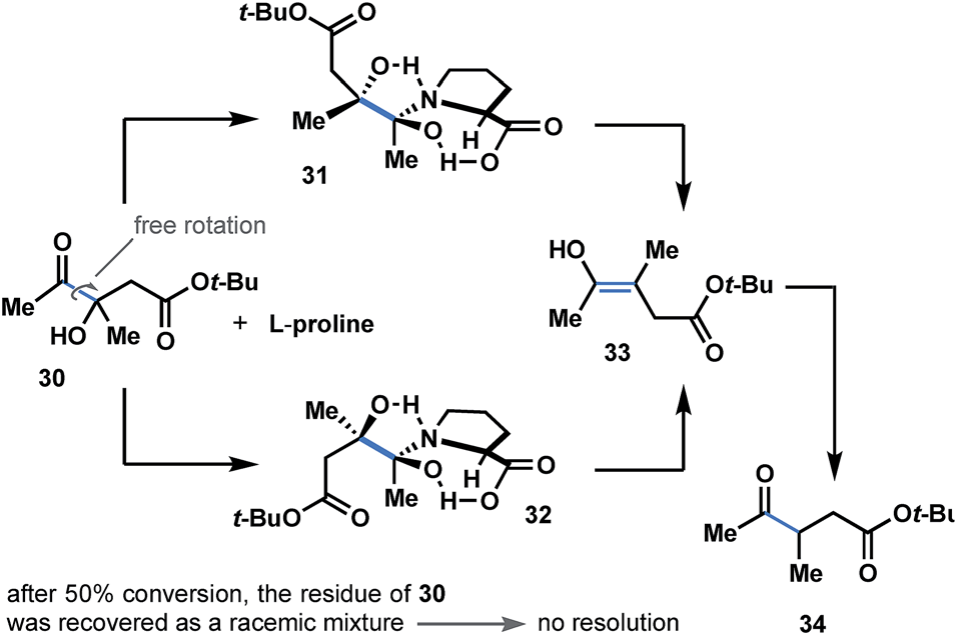
Attempted kinetic resolution of racemic ketol. Both enantiomers of **30** react in a similar way with l-proline to generate dihydroxy-intermediate **31** and **32** with no resolution.

Considering the importance of steric and topological factors, the next question in mind was whether proline-promoted kinetic resolution might be achieved through the dehydroxylation course of α-hydroxyketones. To answer this question, we prepared hydroxyketone **30** (see ESI†) and subjected both enantiomers to the reaction conditions (l-proline; Fig. 6). After 50% conversion of the starting material, the residue of **30** was recovered and detected as a racemic mixture (see ESI†). This brings us to a conclusion that both enantiomers of **30** react in a similar way with l-proline to generate dihydroxypyrrolidinium intermediates **31b** and **32b** (Fig. 6) with no resolution.

### Scope

**Fig. 7:**
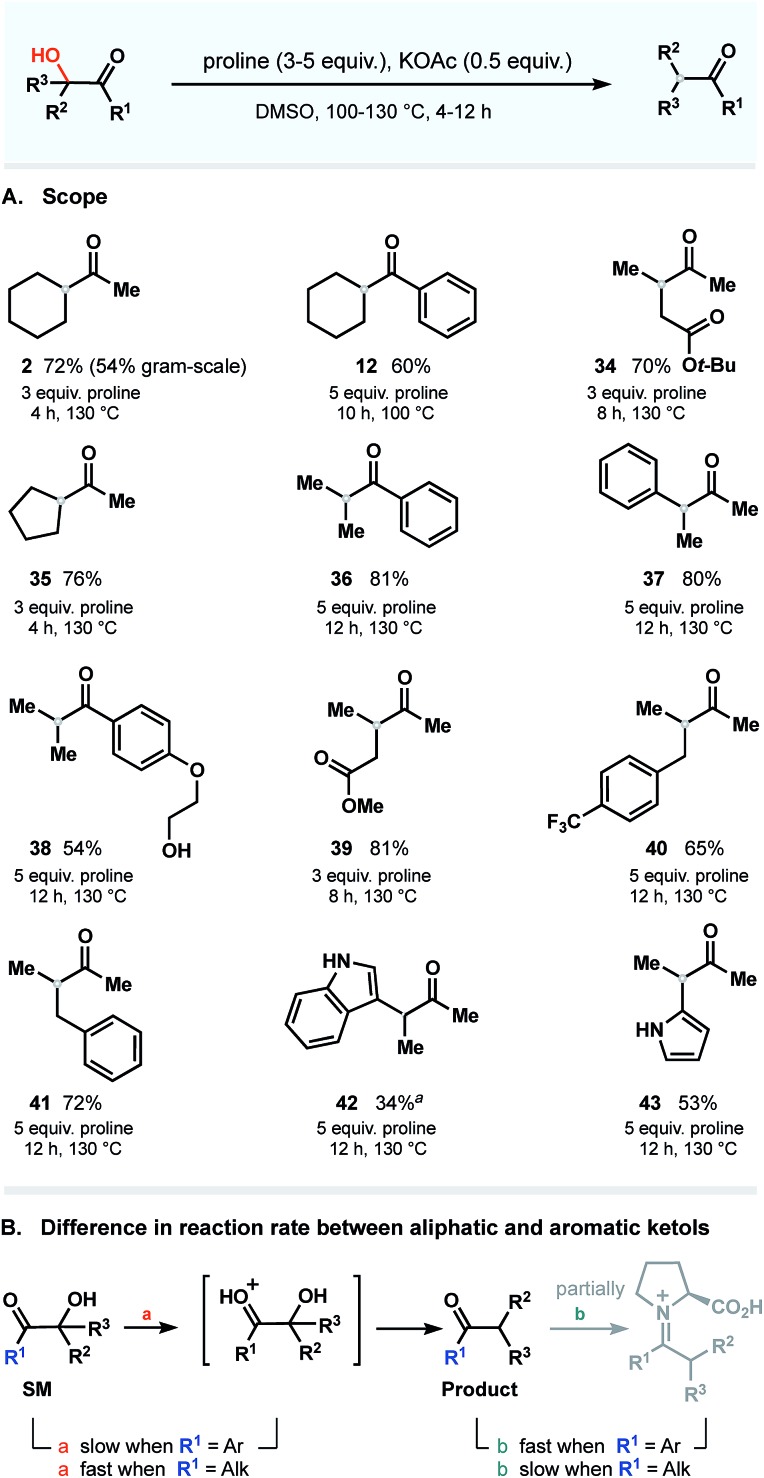
Scope: all reactions were performed with 0.5–1.0 mmol of the substrate (isolated yields). ^*a*^ Incomplete conversion within 24 h (starting material recovered); all attempts to increase the conversion rate by elevating the temperature or prolonging the reaction time resulted in decomposition of starting material and rapid dimerization of proline.

Next, using the general conditions, we targeted other ketols to explore the substrate scope. The studies were conducted on a 0.5–1.0 mmol scale in the presence of 0.5 equivalent of KOAc and 3–5 equivalents of proline at temperatures ranging from 100 to 130 °C (Fig. 7A). Under these conditions, aromatic and aliphatic ketols were tolerated to provide deoxygenated products in good yields. To our delight, the process occurs in a selective manner and does not affect vulnerable moieties such as primary alcohols (**38**), unprotected amines (**42** and **43**), ether (**38**) and ester groups (**34** and **39**). There is, however, a clear dependence of the rate on the nature of the starting ketols. We noticed that the reaction rates of substrates bearing phenone cores differ from those of aliphatic ketols and that prolonged times were required to complete the dehydroxylation. These observations are summarized in Fig. 7B.

**Fig. 8:**
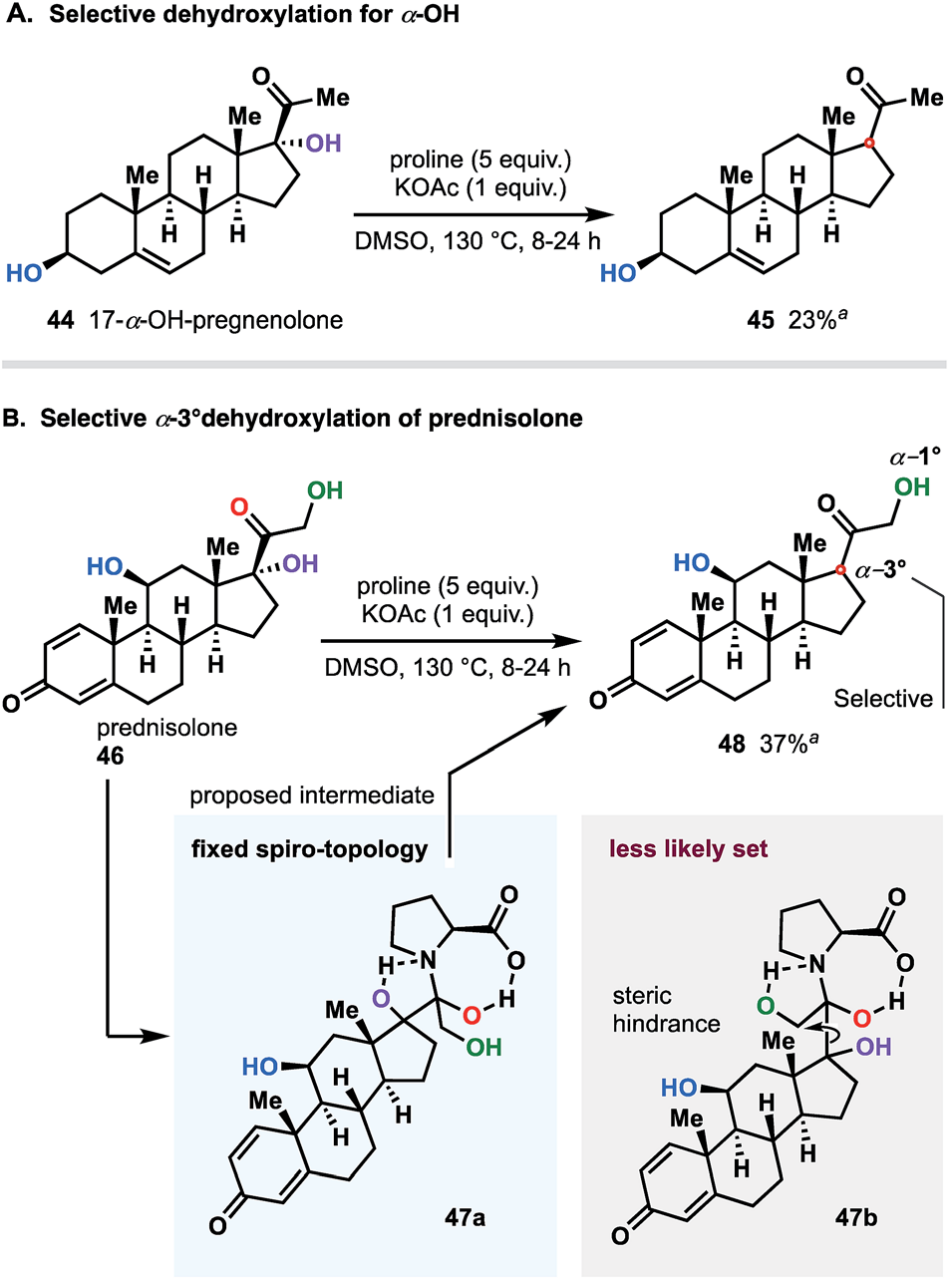
Application of methodology to natural and synthetic steroid drugs: late-stage selective dehydroxylation. All reactions were performed with 0.5 mmol of starting steroid (isolated yields). ^*a*^ Unreacted starting material was recovered. All attempts to increase the yield by extending reaction time up to 24 hours, were unsuccessful.

We next were interested in challenging the synthetic value of our methodology applying the dehydroxylation protocol to natural product and commonly used synthetic drugs. To this end, we chose a family of steroid substances: prednisolone and 17-α-OH-pregnenolone. As shown in Fig. 8A, 17-α-hydroxypregnenolone was deoxygenated in a selective manner without affecting other hydroxy group. Compound **45** was obtained in 23% yield, with the remaining mass balance of the starting material. Notably, no other products were detected in the reaction mixture.^23^

An interesting twist emerged when, prednisolone (**46**), integrated with two α-OH groups (1° and 3°), was subjected to the reaction conditions (Fig. 8B). Even though the dehydroxylation of both α-hydroxy groups was mechanistically allowed, in practice, we detected exclusive selectivity, which led to the sole reduction of the 3° group. We believe that such a pronounced differentiation between two sites is directly attributable to significant geometrical (steric) variations of the dihydroxypyrrolidinium intermediates **47a** and **47b** resulted through the reaction course of both variants (see Fig. 8B). In this case, similarly to 17-α-hydroxypregnenolone (**44**), considerable amount of bulky unreacted starting material was recovered as well.

### Kinetic isotope effect (KIE)

Lastly, in attempt to identify whether an α-deprotonation of proline-ketol intermediate **15** (Fig. 4A) is the rate-controlling step in the dehydroxylation pathway of **1** (Fig. 4A), we conducted kinetic isotope effect experiments. The reaction profiles of α-H and α-D prolines with aliphatic hydroxycyclo-hexylethane (**1**) and aromatic 2-hydroxy-2-methylphenyl-propanone (starting ketol of product **37**), at 100 °C in DMSO, were evaluated by recording ^1^H NMR spectra of the crude reaction mixtures in regular intervals for a period of 7 hours (see ESI section†). A minor isotope effect was found in both experiments with the *k*_H_/*k*_D_ values of 1.03 (aliphatic) and 1.01 (aromatic). The magnitude of the observed KIE for both hydroxyketones befitting the secondary KIE, attributable to the changes in hybridization of sp^3^ to sp^2^,^22^ which is consistent with the mechanistic pathway proposed in Fig. 4 (**15** → **17**).

## Conclusion

We have reported an unprecedented reactivity of proline and demonstrated, for the first time, its ability to function as a reducing agent in the dehydroxylation process of α-ketols. The synthetic advantage of our method is exemplified by the simple and safe setup. The developed metal-free and open-flask operation generally results in good yields, minimal formation of side products, and allows the challenging reduction of hydroxyketones without affecting other functional groups.

## Conflicts of interest

There are no conflicts to declare.

## Supplementary Material

Supplementary information
