# Supplementary material for: Proline-promoted dehydroxylation of α-ketols
Source: Chem Sci. 2019 Aug 19;10(40):9345–50. doi: 10.1039/c9sc02543j (PMC7006627; doi:10.1039/c9sc02543j)
Supplement: Supplementary file 1 [file SC-010-C9SC02543J-s001.pdf]

Electronic Supplementary Material (ESI) for Chemical Science.
This journal is © The Royal Society of Chemistry 2019

SUPPORTING INFORMATION

Proline-Supported Dehydroxylation of α-Ketols

Yelena Mostinski, David Lankri, Yana Konovalov, Riva Nataf, and Dmitry Tsvelikhovsky

The Institute for Drug Research, Faculty of Medicine, The Hebrew University of Jerusalem, Jerusalem 91120, Israel

E-mail: dmitryt@ekmd.huji.ac.il

Table of contents:

General information	S1
Synthesis of α–hydroxyketones (general procedures A, B, C And D)	S1-2
General procedure for dehydroxylation of α–hydroxyketones	S2
Conditions evaluation	S2
Optimization of proline-promoted dehydroxylation of α–hydroxyketones	S3
KIE	S3-4
The influence of time on product yield	S5
Kinetic resolution studies	S5
Compounds characterization (Yield, NMR, IR, MP, HRMS)	S6-11
References	S11
NMR spectra	S12-37

General information

- Unless otherwise stated, all reagents were purchased from commercial suppliers and used without further purification. Reactions were monitored by thin-layer chromatography (TLC) on silica gel 60-F_{254} aluminum plates (Merck) and/or gas chromatography-mass spectrometry (GCMS). Visualization of compounds on TLC was accomplished by irradiation with UV light at 254 nm and/or vanillin stain. GCMS Analysis was performed with 'Agilent 7820A' gas chromatograph equipped with 'Agilent 5975' quadrupole mass selective detector, using Agilent HP-5MS capillary column (30 m, 0.25 mm, 0.25 μm film).
- Column chromatography was performed using silica gel 60 (particle size 0.040-0.063 mm) purchased from Sigma-Aldrich.
- Proton and Carbon NMR spectra were recorded on Varian Mercury 300 MHz spectrometer in deuterated solvent. Proton chemical shifts are reported in ppm (δ) relative to tetramethylsilane with the solvent resonance employed as the internal standard ($CDCl_3$, δ 7.26 ppm). ^{13}C Chemical shifts are reported in ppm from tetramethylsilane with the solvent resonance as the internal standard ($CDCl_3$, δ 77.0 ppm). Data is reported as follows: chemical shift, multiplicity (*s* = singlet, *d* = doublet, *t* = triplet, *q* = quartet, *m* = multiplet), integration and coupling constants (Hz).
- High resolution mass spectra were determined on a ThermoScientific LTQ Orbitrap XL (FTMS).
- Infrared (IR) spectra were recorded on a ThermoFischer Scientific NICOLET iS10 spectrometer.

General procedures for preparation of α-hydroxyketones

***Procedure A*:** to a solution of Mg (3.0 equiv.) with I_2 (cat.) in dry THF (0.2 M), was added Alk/Ar-bromide dropwise (1.5 equiv.), under nitrogen atmosphere. The solution was left to stir for 2 hours at room temp followed by 1 h reflux. 2,5-Heptanedione or 2,3-butanedione (1.0 equiv.) was added dropwise under nitrogen atmosphere, in an ice bath. The reaction was left to stir at room temperature for 5 h and then quenched with HCl solution (0.1M, 20 ml). The mixture was extracted with diethyl ether four times. The combined organic layers were washed with brine, dried (Na_2SO_4), filtered, and concentrated under high vacuum. The product was purified by flash chromatography (silica gel, hexane/ethyl acetate) to yield the corresponding α-hydroxyketone.

Procedure B: to a solution of HgO (0.1 equiv.) and H_2SO_4 (0.1 equiv.) in water (2.0 M) warmed to 60 °C, was added alkyne-alcohol (1.0 equiv.) in one portion. The reaction was left to stir at 60 °C for 2 h. The aqueous layer was extracted with ethyl acetate four times. The combined organic layers were washed with brine, dried (Na_2SO_4), filtered and concentrated under high vacuum. The product was purified by flash chromatography (silica gel, hexane/EtOAc) to yield the corresponding α-hydroxyketone. [1]

Procedure C: Diketone (1.0 equiv.), indium powder (1.05 equiv.), NaI (1.6 equiv.) and Alk/ArI-bromide (1.6 equiv.) in dry DMF (0.3 M) were stirred under 50 °C for 2 h. The reaction was quenched with a solution of HCl 0.1 M, and the aqueous layer was extracted with ethyl acetate four times. The combined organic layers were washed with brine, dried (Na_2SO_4), filtered and concentrated under high vacuum. The product was purified by flash chromatography (silica gel, hexane/EtOAc) to yield the corresponding α-hydroxyketone. [2]

Procedure D: amine (1.0 equiv.) and 2,3-butanedione (1.0 equiv.), were mixed in a flask with a catalytic amount of formic acid. TFA (0.03 equiv.) was added slowly in an ice bath, the reaction mixture was stirred at room temperature for 2 h, under nitrogen atmosphere. The reaction was quenched with an aqueous solution of trimethylamine. The aqueous layer was extracted with ethyl acetate four times. The combined organic layers were washed with brine, dried (Na_2SO_4), filtered and concentrated under high vacuum. The product was purified by flash chromatography (silica gel, hexane/EtOAc) to yield the corresponding α-hydroxyketone. [3]

General procedure for dehydroxylation of α-hydroxyketones

To a flask containing proline (3.0-5.0 equiv., 2.5 mmol, 287.5 mg), KOAc (0.5 equiv., 0.25 mmol, 24.5 mg) in DMSO (0.2 M, 2.5 ml) was added α-hydroxyketone (1.0 equiv., 0.5 mmol). The reaction mixture was allowed to stir for 4-12 h at 100-130 ^{0}C. The reaction was quenched with conc. HCl, stirred in an ice bath for 15 min. The aqueous layer was extracted with diethyl ether four times. The combined organic layers were washed with brine, dried (Na_2SO_4), filtered, and concentrated under high vacuum. The product was purified by flash chromatography (silica gel, hexane/EtOAc/Et_2O) to yield the corresponding ketone.

Conditions Evaluation: base, amino acid, solvent, temperature, time

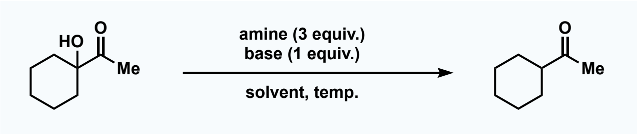

Entry	Solvent	Temp. °C	Time	Base	Amine	GC yield (%)
1	DMSO	25	24	KOAc	Proline	0
2	DMSO	40	24	KOAc	Proline	0
3	DMSO	100	24	KOAc	Proline	92
4	DMSO	100	5	KOAc	Proline	90
5	DMSO	100	10	KOAc	Proline	92
6	DMSO	100	24	NaOAc	Proline	85
7	DMSO	100	24	DBU	Proline	13
8	DMSO	100	24	DIEA	Proline	7
9	DMSO	100	24	Na_2CO_3	Proline	5
10	DMSO	100	24	K_2CO_3	Proline	8
11	DMSO	100	24	Cs_2CO_3	Proline	12
12	DMSO	100	24	K_3PO_4	Proline	5
13	MeOH	100	24	KOAc	Proline	33
14	MeCN	100	24	KOAc	Proline	20
15	DCM	100	24	KOAc	Proline	22
16	THF	100	24	KOAc	Proline	10
17	Acetone	100	24	KOAc	Proline	0
18	Dioxane	100	24	KOAc	Proline	0
19	HFIP	100	24	KOAc	Proline	0
20	DMSO	100	24	KOAc	Pyrrolidine	0
21	DMSO	100	24	KOAc	DIPEA	0
22	DMSO	100	24	KOAc	Morpholine	0
23	DMSO	100	24	KOAc	Sarcosine	0
24	DMSO	100	24	KOAc	Azetidine-carboxylic acid	0
25	DMSO	100	24	KOAc	Alanine	0

Optimization of proline-promoted dehydroxylation of *α*-hydroxyketones

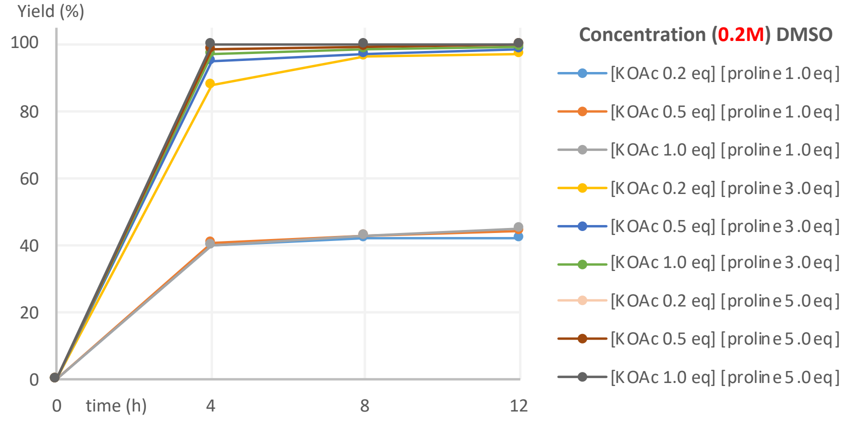

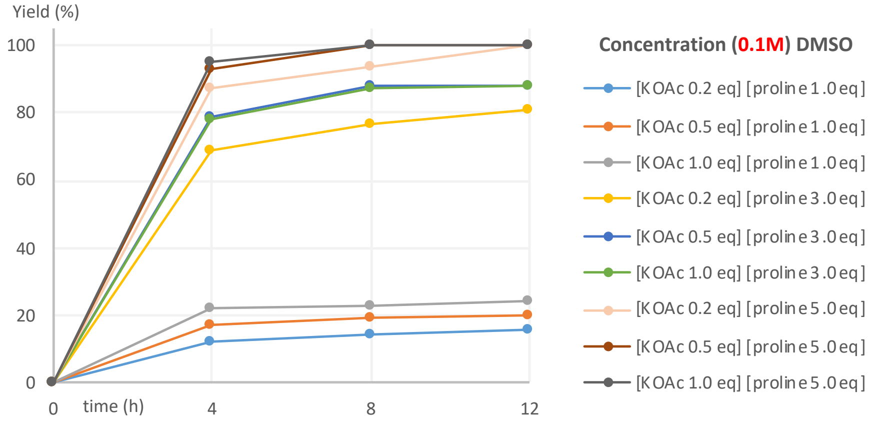

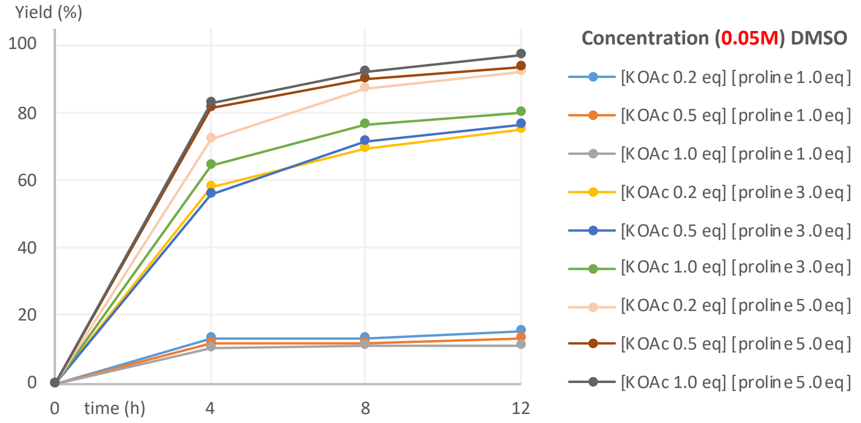

Kinetic Isotope Effect (KIE) studies

Proline-2-d_1 was purchased from Sigma-Aldrich. Cat: 589500, Lot: MBBC6759, assay: 98%. The reaction profiles of α–H and α-D prolines with aliphatic and aromatic hydroxyketones (at 100 °C in DMSO) were evaluated by recording ^{1}H NMR spectra of the crude reaction mixtures in regular intervals for a period of 7 hours.

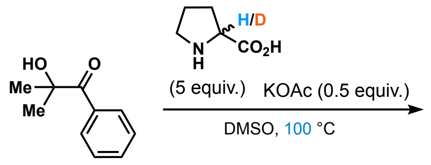

Calculated: KIE = kH/kD = 1.01

Time (h)	Concentration (M) H	Concentration (M) D	ln Concentration H	ln Concentration D
0.00	0.200	0.200	-1.6094379	-1.6094379
0.33	0.199	0.200	-1.6144505	-1.6094379
0.66	0.197	0.199	-1.6245516	-1.6144505
1.00	0.193	0.196	-1.6450651	-1.6296406
3.00	0.170	0.176	-1.7719568	-1.7372713
5.00	0.154	0.162	-1.8708027	-1.8201589
7.00	0.144	0.152	-1.937942	-1.8838748

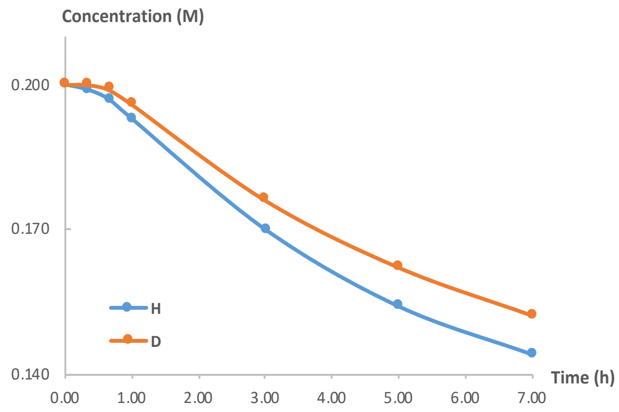

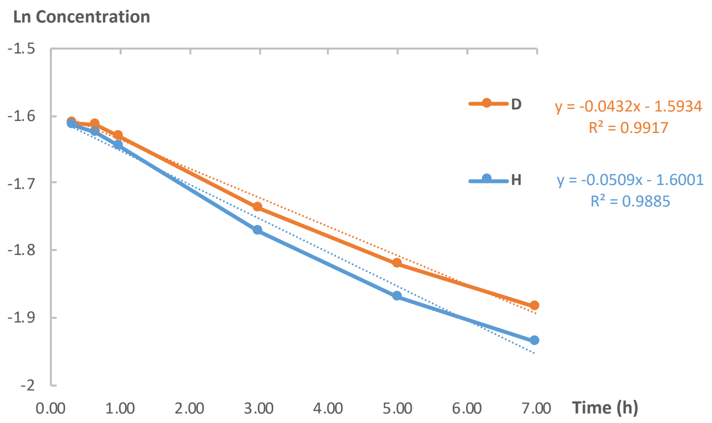

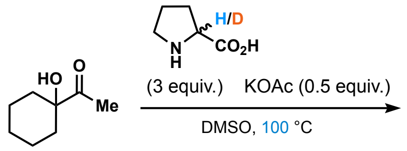

Calculated: KIE = kH/kD = 1.03

Time (h)	Concentration (M) H	Concentration (M) D	ln Concentration H	ln Concentration D
0.00	0.200	0.200	-1.609437912	-1.609437912
0.33	0.172	0.180	-1.760260802	-1.714798428
0.66	0.161	0.170	-1.826350914	-1.771956842
1.00	0.156	0.164	-1.857899272	-1.807888851
3.00	0.138	0.144	-1.980501594	-1.937941979
5.00	0.128	0.134	-2.055725015	-2.009915479
7.00	0.116	0.124	-2.154165088	-2.087473713

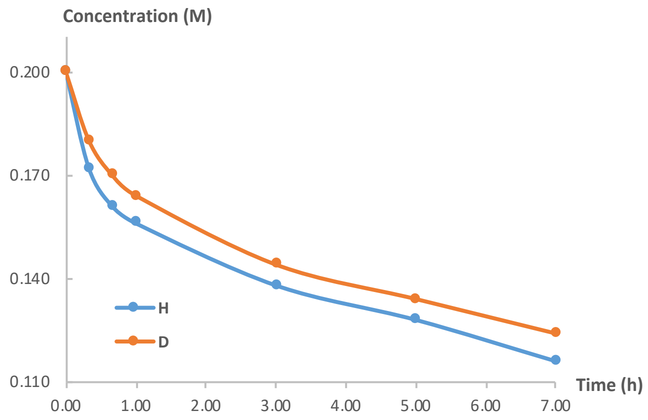

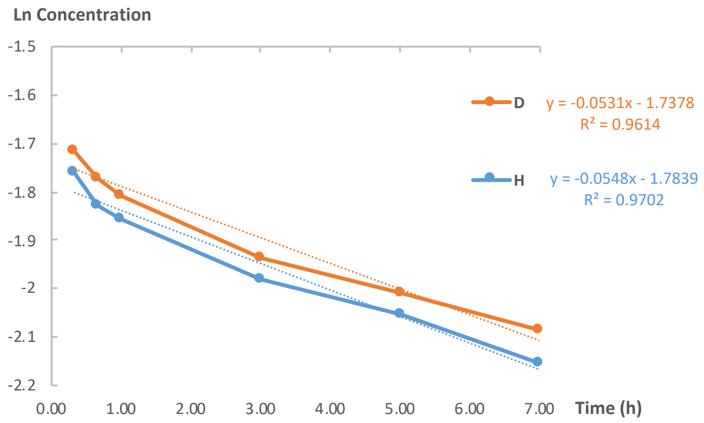

The influence of time on product yield

When calculating the amount of resulting ketone 2, a slight increase in the yield was recorded once the reaction mixture was acidified with HCl, as opposed to the untreated crude mixture. We believe that an excess of proline in the reaction mixture could have affected the yield of reduced α-ketol, promoting side-reactions of the product to generate enamine or oxazolidone. To test this assumption, we conducted an experiment in which the resultant ketone **2** was isolated and re-subjected to the reaction conditions. Partial conversion into enamine was detected. Further treatment of the mixture with HCl left no traces of side products, and the ketone **2** was recovered. Importantly, no enamine derived from the starting ketol was detected in the reaction mixture under the optimized conditions.

We also studied the reaction profile of **1** with proline in the presence of KOAc. We monitored the course of reaction using NMR and GCMS techniques. As we expected, a slight decrease in yield (before workup) was observed.

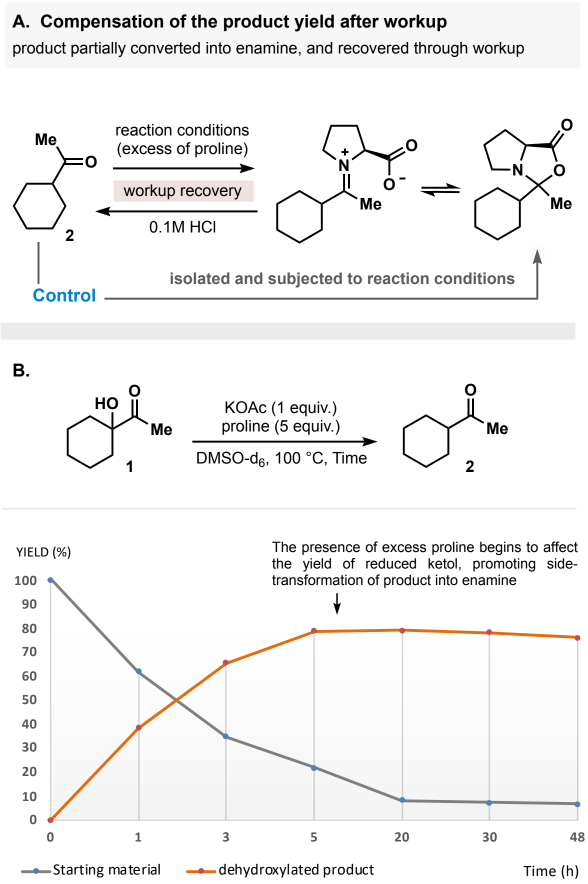

Kinetic resolution studies

Optical rotations were determined using OPTICAL ACTIVITY LTD AA-10R Polarimeter (240V, 50/60 Hz, 250 mA). Solutions were prepared in Methanol. Rotation measurements were recorded with sodium lamp (589.44 nm) at 26 °C using a 2 dm cell. Methyl-3-hydroxy-3-methyl-4-oxopentanoate (0.5mmol) was dehydroxylated to yield ketone **33** (according to General Procedure) and stopped at 50% conversion (the reaction was monitored by ^{1}H NMR in DMSO-d_6).

The crude product was purified by flash chromatography (15% ethyl acetate in hexane) to afford the corresponding **33** (32.0 mg, 34% yield) and the recovered racemic hydroxyketone **30** (34.6 mg, 34% yield)

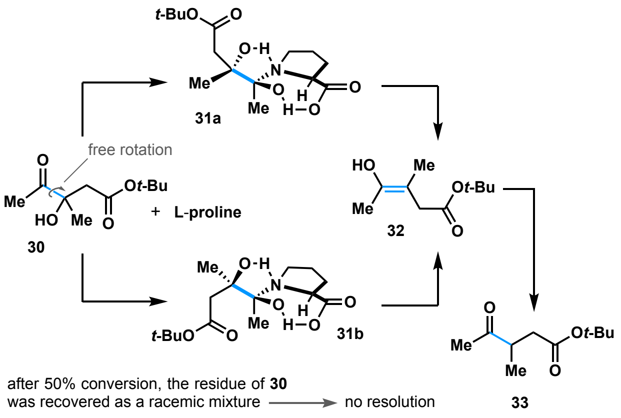

Hydroxyketones

Following α–hydroxyketones were purchased from Merck:

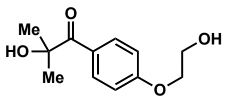

CAS Number: **106797-53-9**

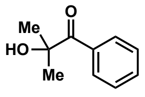

CAS Number: **7473-98-5**

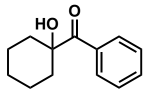

CAS Number: **947-19-3**

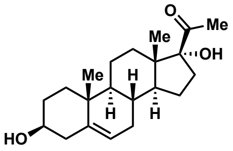

17-α-OH-pregnenolone

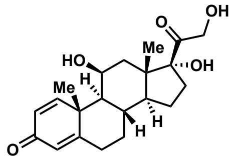

prednisolone

Prepared:

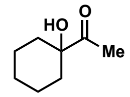

1-(1-Hydroxycyclohexyl)ethanone [CAS: 1123-27-9]**:** general procedure **B** was applied using 1-ethynyl-cyclohexanol (1.00 equiv., 2.00 g, 16.00 mmol), HgO (0.10 equiv., 0.20 g, 1.00 mmol), H_2SO_4 (0.30 ml, 1.70 mmol) in water (2.0 M, 8.00 ml). Purification of the crude product by flash column chromatography (10% diethyl ether in hexane) yielded α-hydroxyketone (1.60 g, 76% yield, yellow oil). **^{1}H NMR** (300 MHz, $CDCl_3$): δ 3.68-3.51 (*s*, 1H), 2.28-2.10 (*s*, 3H), 1.76-1.50 (*m*, 7H), 1.50-1.33 (*m*, 2H), 1.33-1.11 (*m*, 1H). **^{13}C NMR** (75 MHz, $CDCl_3$): δ 213.0, 78.0, 33.7, 25.2, 23.71, 21.0. **IR** (neat): 3464, 2932, 2858, 1700, 1448, 1350, 1148, 983 cm^{-1}.

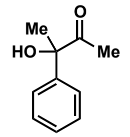

3-Hydroxy-3-phenylbutan-2-one [CAS: 3155-01-9]**:** general procedure **A** was applied using bromobenzene (1.50 equiv., 2.7 g, 17.40 mmol), 2,3-butanedione (1.00 equiv., 1.0 g, 11.60 mmol), Mg (3.00 equiv., 0.84 g) in dry THF (0.2 M, 50.00 ml). Purification of the crude product by flash column chromatography (15% ethyl acetate in hexane) yielded α-hydroxyketone (0.80 g, 42% yield, yellow oil). **^{1}H NMR** (300 MHz, $CDCl_3$): δ 7.65 –7.04 (*m*, 5H), 4.62-4.47 (*s*, 1H), 2.16-1.98 (*s*, 3H), 1.861.73 (*s*, 3H). **^{13}C NMR** (75 MHz, $CDCl_3$): δ 209.7, 141.3, 128.7, 128.1, 125.9, 79.9, 24.0, 23.5. **IR** (neat): 3455, 2980, 1707, 1599, 493, 1133, 698 cm^{-1}.

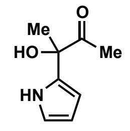

3-Hydroxy-3-(1H-pyrrol-2-yl)butan-2-one [CAS: 35893-38-0]**:** general procedure **D** was applied using pyrrole (1.00 equiv., 0.80 ml, 11.60 mmol,), 2,3-butanedione (1.00 equiv., 1.00 g, 11.60 mmol,), formic acid (0.01 mmol), TFA (0.03 equiv., 0.03 ml, 0.32 mmol,). Purification of the crude product by flash column chromatography (20% EtOAc in hexane) yielded α-hydroxyketone (1.30 g, 75% yield, brown oil). **^{1}H NMR** (300 MHz, $CDCl_3$): δ 8.65-8.25 (*s*, 1H), 6.84-6.62 (*m*, 1H), 6.45 – 5.92 (*m*, 2H), 4.71-4.46 (*s*, 1H), 2.28-2.08 (*s*, 3H), 1.79-1.64 (*s*, 3H). **^{13}C NMR** (75 MHz, $CDCl_3$): δ 209.4, 131.4, 118.2, 109.0, 106.4, 77.0, 24.8, 23.0. **IR** (neat): 3391, 3103, 2986, 1704, 1354, 1102, 793, 723 cm^{-1}.

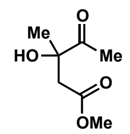

Methyl-3-hydroxy-3-methyl-4-oxopentanoate [CAS: 1824400-96-5]**:** general procedure **C** was applied using methyl-2-bromoacetate (1.55 equiv., 4.70 mmol, 0.72 g), 2,3-butanedione (1.00 equiv., 3.00 mmol, 0.25 g), NaI (1.55 equiv., 4.70 mmol, 0.70 g) and indium powder (1.05 equiv., 3.15 mmol, 0.36 g) in dry DMF (0.3 M, 10.00 ml). Purification of the crude product by flash column chromatography (20% ethyl acetate in hexane) yielded α-hydroxyketone (0.35 g, 80% yield, colorless liquid). **^{1}H NMR** (300 MHz, $CDCl_3$): δ 4.29-4.20 (*s*, 1H), 3.65-3.55 (*s*, 3H), 3.00-2.85 (*d*, *J* = 16.5 Hz, 1H), 2.54-2.51 (*d, J* = 16.5 Hz, 1H), 2.29-2.18 (*s*, 3H), 1.29-1.21 (*s*, 3H). **^{13}C NMR** (75 MHz, $CDCl_3$):

δ 211.9, 172.3, 77.3, 51.92, 43.0, 25.1, 24.0. **IR** (neat): 3495, 2981, 2957, 2925, 2854, 1711, 1642, 1353, 1162, 1001, 727 cm^{-1}.

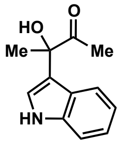

3-Hydroxy-3-(1H-indol-3-yl)butan-2-one: general procedure **D** was applied using Indole (1.00 equiv., 3.00 mmol, 0.35 g), 2,3-butanedione (1.00 equiv., 3.00 mmol, 0.25 g), formic acid (0.01 mmol), TFA (0.03 equiv., 10.26 mg). Purification of the crude product by flash column chromatography (20% ethyl acetate in hexane, on TEA treated silica) yielded α-hydroxyketone (0.46 g, 75% yield, beige powder). **^{1}H NMR** (300 MHz, DMSO-d_6): δ 11.21-10.85 (*s*, 1H), 7.58-7.43 (*d, J* = 8.0 Hz, 1H), 7.37 – 7.22 (*d, J* = 8.0 Hz, 1H), 7.07- 6.96 (*t, J* = 7.6 Hz, 1H), 6.95-6.86 (*t, J* = 7.6 Hz, 1H), 5.71-5.62 (*s*, 1H), 3.36-3.27 (*s*, 1H), 2.55-2.41 (*s*, 3H), 2.09-1.98 (*s*, 3H), 1.67-1.55 (*s*, 3H). **^{13}C NMR** (75 MHz, $CDCl_3$): δ 211.1, 136.8, 125.0, 122.9, 122.4, 120.1, 119.8, 116.6, 111.4, 76.9, 24.5, 23.4. **IR** (neat): 3452, 3295, 1703, 1665, 1659, 1458, 1372, 1118, 738 cm^{-1}. **MP**: 181-183 °C. **HRMS** (*m/z*) calc. for $C_{12}H_{13}NO_2Na$ ($[M+Na]^+$): 226.0840; found: 226.0839.

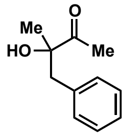

3-hydroxy-3-methyl-4-phenylbutan-2-one [CAS: 54123-76-1]**:** general procedure **A** was applied using benzyl bromide (1.50 equiv., 2.90 g, 17.40 mmol), 2,3-butanedione (1.00 equiv., 1.00 g, 11.60 mmol), Mg (3.00 equiv., 0.84 g) in dry THF (0.2 M, 50.00 ml). Purification of the crude product by flash column chromatography (15% ethyl acetate in hexane) yielded α-hydroxyketone (1.40 g, 72% yield, yellow oil). **^{1}H NMR** (300 MHz, $CDCl_3$): δ 7.39 – 7.04 (*m*, 5H), 3.63-355 (*s*, 1H), 3.06-2.91 (*d, J* = 4.0 Hz, 2H), 2.27-2.17 (*s*, 3H), 1.45-1.37 (*s*, 3H).**^{13}C NMR** (75 MHz, $CDCl_3$): δ 211.7, 135.6, 129.9, 128.3, 126.9, 79.3, 45.3, 25.0, 24.5. **IR** (neat): 3390, 3028, 2976, 2919, 1705, 1603, 1583, 1495, 1018, 700 cm^{-1}.

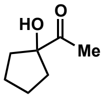

1-(1-Hydroxycyclopentyl)ethanone [CAS: 17160-89-3]**:** general procedure **B** was applied using 1-ethynylcyclopentanol (1.00 equiv., 1.50 g, 13.60 mmol), HgO (0.06 equiv., 0.17 g, 0.80 mmol), H_2SO_4 (0.30 ml, 1.70 mmol) in water (2.0 M, 6.00 ml). Purification of the crude product by flash column chromatography (10% diethyl ether in hexane) yielded α-hydroxyketone (1.35 g, 78% yield, pale yellow oil). **^{1}H NMR** (300 MHz, $CDCl_3$): δ 2.25-2.18 (*s*, 3H), 2.07-1.86 (*m*, 4H), 1.86-1.64 (*m*, 4H). **^{13}C NMR** (75 MHz, $CDCl_3$): δ 212.1, 87.0, 38.9, 25.4, 23.7. **IR** (neat): 3444, 2959, 2873, 1699, 1437, 1354, 1168, 621 cm^{-1}.

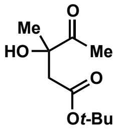

Tert-butyl 3-hydroxy-3-methyl-4-oxopentanoate: general procedure **C** was applied using *tert*-Butyl 2-bromoacetate (1.55 equiv., 7.70 mmol, 1.50 g), 2,3-butanedione (1.00 equiv., 5.00 mmol, 0.43 g), NaI (1.55 equiv., 7.70 mmol, 1.20 g) and indium powder (1.05 equiv., 5.25 mmol, 0.60 g) in dry DMF (0.30 M, 17.00 ml). Purification of the crude product by flash column chromatography (30% ethyl acetate in hexane) yielded the α-hydroxyketone (0.42 g, 42% yield, colorless liquid). **^{1}H NMR** (300 MHz, $CDCl_3$): δ 4.42-4.43 (*s*, 1H), 2.96-2.86 (*d, J* = 16.3 Hz, 1H), 2.57-2.46 (*d, J* = 16.4 Hz, 1H), 2.31-2.25 (*s*, 3H), 1.42-1.38 (*s*, 3H), 1.29-1.25 (*s*, 3H). **^{13}C NMR** (75 MHz, $CDCl_3$): δ 212.1, 171.2, 82.0, 44.3, 27.9, 25.1, 24.1. **IR** (neat): 3467, 2978, 2934, 1712, 1416, 1367, 1148, 1107, 841 cm^{-1}. **HRMS** (*m/z*) calc. for $C_{10}H_{18}O_4Na$ ($[M+Na]^+$): 225.1098 ; found: 225.1097.

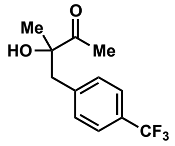

3-Hydroxy-3-methyl-4-(4-(trifluoromethyl)phenyl)butan-2-one: general procedure **C** was applied using 1-(bromomethyl)-4-(trifluoromethyl)benzene (1.55 equiv., 7.70 mmol, 1.8 g), 2,3-butanedione (1.00 equiv., 5.00 mmol, 0.43 g), NaI (1.55 equiv., 7.70 mmol, 1.20 g) and indium powder (1.05 equiv., 5.25 mmol, 0.60 g) in dry DMF (0.3 M, 17.0 ml). Purification of the crude product by flash column chromatography (40% ethyl acetate in hexane) yielded α-hydroxyketone (0.77 g, 63% yield, colorless liquid). **^{1}H NMR** (300 MHz, $CDCl_3$): δ 7.55-7.47 (*d, J* = 7.9 Hz, 2H), 7.39-7.27 (*d, J* = 8.0 Hz, 2H), 3.75-3.62 (*s*, 1H), 3.07-3.98 (*s*, 2H), 2.32-2.21 (*s*, 3H), 1.45-4.36 (*s*, 3H). **^{13}C NMR** (75 MHz, $CDCl_3$): δ 211.0, 139.7, 130.3 (4C), 125.1 (*q*, *J* = 3.9, 1C), 79.0, 44.8, 25.0, 24.2. **^{19}FNMR** (282 MHz, $CDCl_3$): δ -62.51. **IR** (neat): 3477, 3070, 1708, 1669, 1619, 1418, 1322, 1159, 1065 cm^{-1}. **HRMS** (*m/z*) calc. for $C_{12}H_{13}F_3O_2Na$ ($[M+Na]^+$): 269.0760; found: 269.0757.

Products

General procedure for dehydroxylation α-hydroxyketones was applied (see S2):

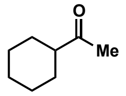

1-cyclohexylethanone [CAS: 823-76-7]**:** 1-(1-hydroxycyclohexyl)ethanone (71.00 mg) was used. 3 equiv. of proline, 4 h, at 130 ^{0}C. The product was purified by flash chromatography (5% ether in hexane) to afford *1-cyclohexylethanone* (45.00 mg, 72% yield, colorless liquid). **^{1}H NMR** (300 MHz, $CDCl_3$): δ 2.37-2.23 (*m*, 1H), 2.11-2.06 (*s*, 3H), 1.91 – 1.71 (*m*, 4H), 1.69 – 1.56 (*m*, 1H), 1.38-1.10 (*m*, 5H). **^{13}C NMR** (75 MHz, $CDCl_3$): δ 212.4, 51.4, 28.4, 27.9, 25.8, 25.6. **IR** (neat): 3400, 2927, 2854, 1741, 1707, 1449, 1352, 1166 cm^{-1}. **1-g Scale experiment:** 1-(1-hydroxycyclohexyl)ethanone (1.00 g, 7.00 mmol) was used. 10 h, at 100 °C. The product was purified by flash chromatography (5% ether in hexane) to yield 1-cyclohexylethanone (477.00 mg, 54% yield, colorless liquid).

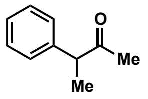

3-phenylbutan-2-one [CAS: 769-59-5]**:** 3-hydroxy-3-phenylbutan-2-one (82.00 mg) was used. 5 equiv. of proline, 12 h, 130 °C. The product was purified by flash chromatography (10% ethyl acetate in hexane) to afford *3-phenylbutan-2-one* (40.00 mg, 80% yield, yellow liquid). **^{1}H NMR** (300 MHz, $CDCl_3$): δ 7.37 – 7.27 (*m*, 2H), 7.27 – 7.17 (*m*, 3H), 3.80-3.68 (*q*, *J* = 7.0 Hz, 1H), 2.10-2.00 (*s*, 3H), 1.41-1.35 (*d*, *J* = 7.0 Hz, 3H). **^{13}C NMR** (75 MHz, $CDCl_3$): δ 208.9, 140.6, 128.9, 127.8, 127.2, 53.7, 28.4, 17.2. **IR** (neat): 3027, 2976, 2931, 2359, 1712, 1600, 1493, 1372, 1164, 699 cm^{-1}.

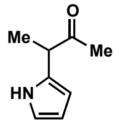

3-(1H-pyrrol-2-yl)butan-2-one: 3-hydroxy-3-(1H-pyrrol-2-yl)butan-2-one (77.0 mg) was used. 5 equiv. of proline, 12 h, 130 °C. The product was purified by flash chromatography (20% ethyl acetate in hexane) to afford *3-(1H-pyrrol-2-yl)butan-2-one* (38.00 mg, 53% yield, brown-yellow liquid). **^{1}H NMR** (300 MHz, $CDCl_3$): δ 8.47-8.12 (*s*, 1H), 6.79-6.66 (*m*, 1H), 6.06 – 6.03 (*m*, 1H), 6.16 – 6.13 (*m*, 1H),3.90-3.71 (*q, J* = 7.2 Hz, 1H), 2.25-2.03 (*s*, 3H), 1.49-1.37 (*d, J* = 7.2 Hz, 3H). **^{13}C NMR** (75 MHz, $CDCl_3$): δ 209.7, 129.6, 117.9, 108.4, 106.2, 46.4, 27.7, 16.6. **IR** (neat): 3369, 2956, 2926, 2872, 2855, 1704, 1639, 1555, 1355, 723 cm^{-1}. **HRMS** (*m/z*) calc. for $C_8H_{11}NONa$ ($[M+Na]^+$): 160.0733; found: 160.0733.

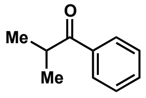

2-methyl-1-phenylpropan-1-one [CAS: 611-70-1]**:** 2-hydroxy-2-methyl-1-phenylpropan-1-one (164.0 mg) was used. 5 equiv. of proline, 12 h, 130 °C. The product was purified by flash chromatography (20% ethyl acetate in hexane) to afford *2-methyl-1-phenylpropan-1-one* (120.30 mg, 81% yield, colorless liquid). **^{1}H NMR** (300 MHz, $CDCl_3$): δ 8.00 – 7.91 (*m*, 2H), 7.58 – 7.57 (*m*, 1H), 7.56 – 7.43 (*m*, 2H), 3.66-3.43 (*p, J* = 6.8 Hz, 1H), 1.28-1.13 (*d, J* = 6.8 Hz, 6H). **^{13}C NMR** (75 MHz,

CDCl$_3$): δ 204.5, 136.2, 132.8, 128.6, 128.3, 35.3, 19.1. **IR** (neat): 2971, 2931, 2872, 1682, 1596, 1578, 1222, 977, 699 cm^{-1}.

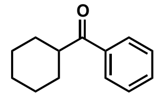

cyclohexyl(phenyl)methanone [CAS: 712-50-5]**:** (1-hydroxy-cyclohexyl)phenyl-methanone (102.0 mg) was used. 5 equiv. of proline, 10 h, 130 °C. The product was purified by flash chromatography (40% ethyl acetate in hexane) to afford *cyclohexyl(phenyl)methanone* (56.00 mg, 60% yield, yellow oil). **^{1}H NMR** (300 MHz, CDCl$_3$): δ 8.12 – 7.79 (*d, J* = 7.0 Hz, 2H), 7.63 – 7.49 (*m*, 3H), 3.29-3.22 (*m*, 1H), 1.91-1.70 (*m*, 5H), 1.52 – 1.23 (*m*, 5H). **^{13}C NMR** (75 MHz, CDCl$_3$): δ 203.9, 136.3, 132.7, 128.6, 128.2, 45.6, 29.4, 25.9, 25.9. **IR** (neat): 2927, 2853, 1679, 1596, 1579, 1462, 1289, 1206, 972, 695 cm^{-1}.

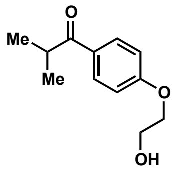

1-(4-(2-hydroxyethoxy)phenyl)-2-methylpropan-1-one [CAS: 159119-01-4]**:** 2-hydroxy-1-(4-(2-hydroxy-ethoxy)phenyl)-2-methylpropan-1-one (112.0 mg) was used. 5 equiv. of proline, 12 h, 130 °C. The product was purified by flash chromatography (60% ethyl acetate in hexane) to afford *1-(4-(2-hydroxyethoxy)phenyl)-2-methylpropan-1-one* (35.00 mg, 53% yield, colorless oil). **^{1}H NMR** (300 MHz, CDCl$_3$): δ 8.07 – 7.85 (*d, J* = 8.9 Hz, 2H), 7.04 – 6.81 (*d, J* = 8.9 Hz, 2H), 4.18-4.04 (*m*, 2H), 4.05-3.92 (*t, J* = 4.5 Hz, 2H), 3.57-3.43 (*p, J* = 6.8 Hz, 1H), 1.22-1.12 (*d, J* = 6.8 Hz, 6H). **^{13}C NMR** (75 MHz, CDCl$_3$): δ 203.2, 162.3, 130.6, 129.4, 114.2, 69.3, 61.3, 34.9, 19.3. **IR** (neat) 3386, 2969, 2931, 2872, 1670, 1597, 1573, 1224, 1157, 979, 632 cm^{-1}.

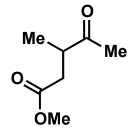

methyl-3-methyl-4-oxopentanoate [CAS: 25234-83-7]**:** methyl-3-hydroxy-3-methyl-4-oxo-pentanoate (80.0 mg) was used. 3 equiv. of proline, 8 h, 130 °C. The product was purified by flash chromatography (15% ethyl acetate in hexane) to afford *methyl-3-methyl-4-oxopentanoate* (60.00 mg, 81% yield, colorless oil). **^{1}H NMR** (300 MHz, CDCl$_3$): δ 3.68-3.59 (*s*, 3H), 3.14 – 2.91 (*m*, 1H), 2.83-2.66 (*dd, J* = 16.8, 8.7 Hz, 1H), 2.36-2.23 (*dd, J* = 16.8, 5.4 Hz, 1H), 2.21-2.16 (*s*, 3H), 1.18-1.08 (*d, J* = 7.3 Hz, 3H). **^{13}C NMR** (75 MHz, CDCl$_3$): δ 210.8, 172.8, 51.7, 42.7, 36.6, 28.4, 16.6. **IR** (neat) 2970, 2925, 2854, 1736, 1713, 1457, 1437, 1356, 1274, 1202, 1166, 1085, 1047, 1010, 880 cm^{-1}.

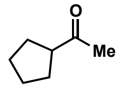

1-cyclopentylethanone [CAS: 6004-60-0]**:** 1-(1-hydroxycyclopentyl)-ethanone (65 mg) was used. 3 equiv. of proline, 4 h, at 130 °C. The product was purified by flash chromatography (5% ether in hexane) to afford *1-cyclopentylethanone* (0.43 g, 76% yield, colorless oil). **^{1}H NMR** (300 MHz, CDCl$_3$): δ 2.92-2.75 (*q*, *J* = 7.9 Hz, 1H), 2.16-2.08 (*s*, 3H), 1.82 – 1.53 (*m*, 8H). **^{13}C NMR** (75 MHz, CDCl$_3$): δ 211.3, 52.2, 28.8, 28.7, 25.9. **IR** (neat): 3355, 2924, 2852, 1643, 1450, 1017 cm^{-1}.

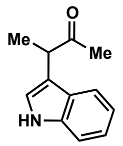

3-(1H-indol-3-yl)butan-2-one: 3-hydroxy-3-(1H-indol-3-yl)butan-2-one (102.0 mg) was used. 5 equiv. of proline, 12 h, 130 °C. The product was purified by flash chromatography (40% ethyl acetate in hexane, on neutralized silica using TEA) to afford *3-(1H-indol-3-yl)butan-2-one* (33.00 mg, 34% yield, colorless oil). **^{1}H NMR** (300 MHz, CDCl$_3$): δ 8.23-8.04 (*s*, 1H), 7.57 – 7.60 (*d, J* = 8.2 Hz, 1H), 7.39-7.36 (*d, J* = 8.1, 1.0 Hz, 1H), 7.25 – 7.08 (*m*, 3H), 4.09-3.94 (*q, J* = 7.0 Hz, 1H), 2.11-2.04 (*s*, 3H), 1.54-1.45 (*d, J* = 7.0 Hz, 3H). **^{13}C NMR** (75 MHz, CDCl$_3$): δ 210.1, 168.1, 136.3, 122.4, 121.8, 119.8, 118.9, 115.5, 111.3, 44.9, 27.6, 16.5. **IR** (neat): 3407, 305, 2973, 2930, 2872, 1697, 1618, 1456, 1352, 1097, 740 cm^{-1}. **HRMS** (*m/z*) calc. for $C_{12}H_{13}NONa$ ([M+Na]$^+$): 210.0895; found: 210.0889.

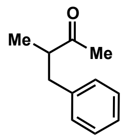

3-methyl-4-phenylbutan-2-one [CAS: 2550-27-8]**:** 3-hydroxy-3-methyl-4-phenylbutan-2-one (90.0 mg) was used. 5 equiv. of proline, 12 h, 130 °C. The product was purified by flash chromatography (20% ethyl acetate in hexane) to afford *3-methyl-4-phenylbutan-2-one* (60.00 mg, 72% yield, yellow oil). ^{1}**H NMR** (300 MHz, $CDCl_3$): *δ* 7.33 –7.08 (*m*, 5H), 3.07-2.92 (*dd, J* = 13.4, 6.7 Hz, 1H), 2.91-2.72 (*h, J* = 7.0 Hz, 1H), 2.62-2.47 (*dd, J* = 13.4, 7.7 Hz, 1H), 2.13-2.01 (*s*, 3H), 1.19-1.03 (*d, J* = 6.9 Hz, 3H). ^{13}**C NMR** (75 MHz, $CDCl_3$): *δ* 212.3, 139.6, 129.0, 128.6, 126.6, 48.8, 38.9, 29.1, 16.2. **IR** (neat): 3556, 3027, 2970, 2930, 2748, 1709, 1602, 1496, 1358, 955, 755, 698 cm^{-1}.

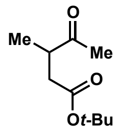

tert-butyl-3-methyl-4-oxopentanoate [CAS: 951217-76-8]**:** *tert*-butyl-3-hydroxy-3-methyl-4-oxopentanoate was used (101.0 mg). 3 equiv. of proline, 8 h, 130 °C. The product was purified by flash chromatography (30% ethyl acetate in hexane) to afford *tert-butyl-3-methyl-4-oxopentanoate* (66.00 mg, 70% yield, colorless liquid). ^{1}**H NMR** (300 MHz, $CDCl_3$): *δ* 3.01 – 2.77 (*m*, 1H), 2.71-2.56 (*dd, J* = 16.6, 8.6 Hz, 1H), 2.24-2.14 (*dd, J* = 16.6, 8.6 Hz, 1H), 2.19-2.16 (*s*, 3H), 1.41-1.36 (*s*, 9H), 1.12-1.05 (*d, J* = 7.4 Hz, 3H). ^{13}**C NMR** (75 MHz, $CDCl_3$): *δ* 210.9, 171.5, 80.6, 42.9, 38.2, 28.4, 28.0, 16.4. **IR** (neat): 3540, 3004, 2976, 2932, 2880, 2725, 1715, 1458, 1409, 1149, 847 cm^{-1}.

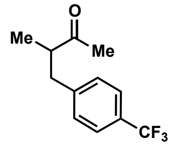

3-methyl-4-(4-(trifluoromethyl)phenyl)butan-2-one [CAS: 1248115-57-2]**:** 3-hydroxy-3-methyl-4-(4-(trifluoromethyl)phenyl)butan-2-one (123 mg) was used. 5 equiv. of proline,12 h, 130 °C. The product was purified by flash chromatography (40% ethyl acetate in hexane) to afford *3-methyl-4-(4-(trifluoromethyl)phenyl)butan-2-one* (75.00 mg, 65% yield, colorless oil). ^{1}**H NMR** (300 MHz, $CDCl_3$): *δ* 7.59-7.46 (*d, J* = 8.0 Hz, 2H), 7.317.21 (*d, J* = 6.1 Hz, 2H), 3.13-2.99 (*dd, J* = 13.5, 6.9 Hz, 1H), 2.91-2.76 (*h, J* = 7.1 Hz, 1H), 2.67-2.53 (*dd, J* = 13.5, 7.4 Hz, 1H), 2.15-2.05 (*s*, 3H), 1.14-1.06 (*d, J* = 7.0 Hz, 3H). ^{13}**C NMR** (75 MHz, $CDCl_3$): *δ* 211.4, 146.0, 143.9, 129.3 (4C), 125.3 (*q*, *J* = 3.9 Hz, 1C) , 48.5, 38.3, 28.8, 16.4. ^{19}**FNMR** (282 MHz, $CDCl_3$): *δ* -62.42. **IR** (neat): 3519, 3046, 2970, 2925, 2855, 2745, 1712, 1618, 1322, 1113, 1066 cm^{-1}.

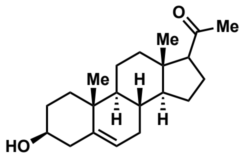

pregnenolone [1950561-02-0]***:*** 17-α-hyfroxypregnenolone (332.5 mg) was used. 5.0 equiv. of proline, 8 h, 130.0 °C. The product was purified by flash chromatography (1 % MeOH/DCM) to obtain pregnenolone as a mixture of diastereomers (38.0 mg, 23.0 % yield, pale yellow solid). d.r. 83:17. ^{1}**H NMR** (300 MHz, $CDCl_3$): mixture of diastereomers: *δ* 5.40-5.31 (m, 1H), 2.57-2.46 (m, 1H), 2.34.2.14 (m, 3H), 2.04-1.92 (m, 2H), 190-172 (m, 5H), 1.69-1.58 (m, 3H), 1.54-1.41 (m, 4H), 1.22-1.11 (m, 2H), 1.10-1.02 (m. 1H). major diastereomer: *δ* 3.57-3.45 (m, 1H), 2.13-2.08 (s, 1H), 1.01-0.97 (s, 3H), 0.65-0.60 (s, 1H). minor diastereomer: *δ* 3.44-3.34 (m. 1H), 2.07-2.04 (s, 3H), 0.92-0.90 (s, 3H), 0.59-0.54 (s, 3H). ^{13}**C NMR** (75 MHz, $CDCl_3$): major diastereomer: δ 209.6, 140.7, 121.4, 71.7, 63.7, 56.9, 49.9, 44.0, 42.2, 38.8, 37.2, 36.5, 31.8, 31.8, 31.6, 31.6, 31.6, 24.5, 22.8, 21.1, 19.4, 13.2. minor diastereomer: *δ* 140.4, 121.6, 77.4, 76.6, 71.6, 61.3, 58.1, 57.3, 50.5, 50.1, 49.9, 49.5, 39.4, 35.1, 34.0, 26.0, 24.8, 20.6, 19.4, 13.7. **IR** (neat): 3431, 2929, 2849, 2359, 1698, 1433, 1355, 1193, 1049, 952, 733 cm^{-1}. [4]

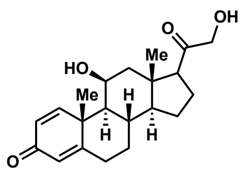

1,2-didehydrocorticosterone [58761-79-8]: prednisolone (180.00 mg, 0.50 mmol) was used. 5 equiv. of proline, 8 h, 130 °C. The product was purified by flash chromatography (5% MeOH/DCM) to obtain 1,2-didehydrocorticosterone (65.0 mg, 37% yield). ^{1}**H NMR** (300 MHz, $CDCl_3$): δ 7.27 (d, *J* = 10.4 Hz, 1H), 6.24 (d, *J* = 10.1 Hz,

10.1 Hz, 1H), 6.01 (s, 1H), 4.44 (s, 0H), 3.44 (*dd*, *J* = 24.2, 6.9 Hz, 2H), 2.65 – 2.44 (m, 2H), 2.38 – 2.06 (m, 5H), 2.03 – 1.82 (m, 5H), 1.74 – 1.56 (m, 2H), 1.46 (s, 3H), 1.45 – 1.36 (m, 1H), 1.16 (s, 3H), 1.08 – 1.01 (m, 1H). **^{13}C NMR** (75 MHz, $CDCl_3$): δ 219.1, 186.6, 169.9, 156.2, 127.8, 122.5, 69.6, 55.9, 51.7, 47.0, 46.1, 44.1, 43.2, 40.7, 35.2, 32.7, 31.8, 30.8, 21.9, 21.1, 15.7. **IR** (neat): 3429, 2924, 1437, 1655, 1616, 1449, 1086, 887, 729 cm^{-1}. [5]

References

1 Stacy, G.W.; Hainley, C. A. *J. Am. Chem. Soc.* **1951**, *73*, 5911.
2 Blomberg, C.; Hartog, F. A. *Synthesis,* **1977**, 18.
3 Ryang, H. S.; Sakurai, H. *J. Chem. Soc., Chem. Commun*., **1972**, 77.
4 Chen, F.; Topf, C.; Radnik, J.; Kreyenschulte, C.; Lund, H.; Schneider, M.; Surkus, A.; He, L.; Junge, K.; Beller, M. *J. Am. Chem. Soc.* **2016**, *138*, 8781.
5 a) Ryang, H. S.; Sakurai, H. *Chem. Pharm. Bull.* **1986**, *34*, 3722. b) Kime, D. E. *J. Chem. Soc. Chem. Comm*. **1972**, 77.

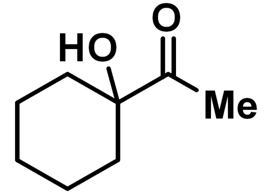
HO
O
Me

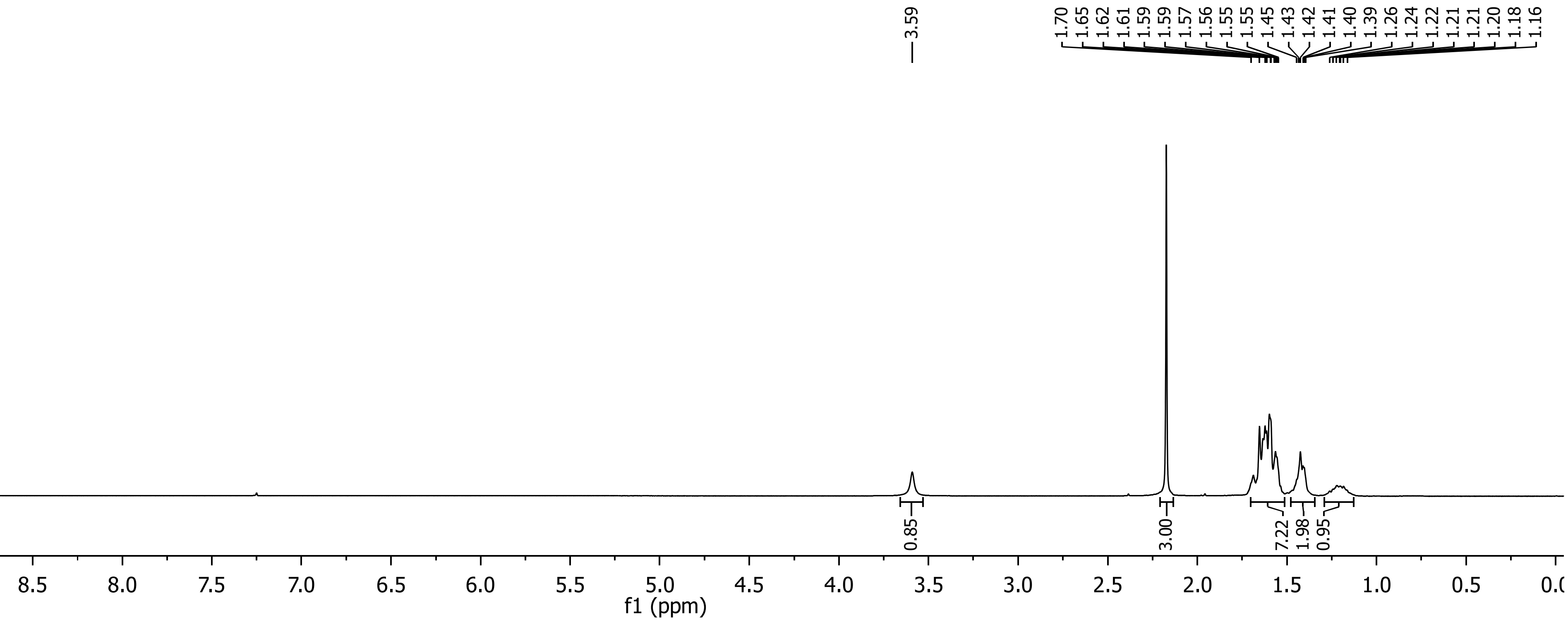
3.59
1.70 1.65 1.62 1.61 1.59 1.59 1.57 1.56 1.55 1.55 1.45 1.43 1.42 1.41 1.40 1.39 1.26 1.24 1.22 1.21 1.21 1.20 1.18 1.16
0.85
3.00
7.22
1.98
0.95
9.5 9.0 8.5 8.0 7.5 7.0 6.5 6.0 5.5 5.0 4.5 4.0 3.5 3.0 2.5 2.0 1.5 1.0 0.5
f1 (ppm)

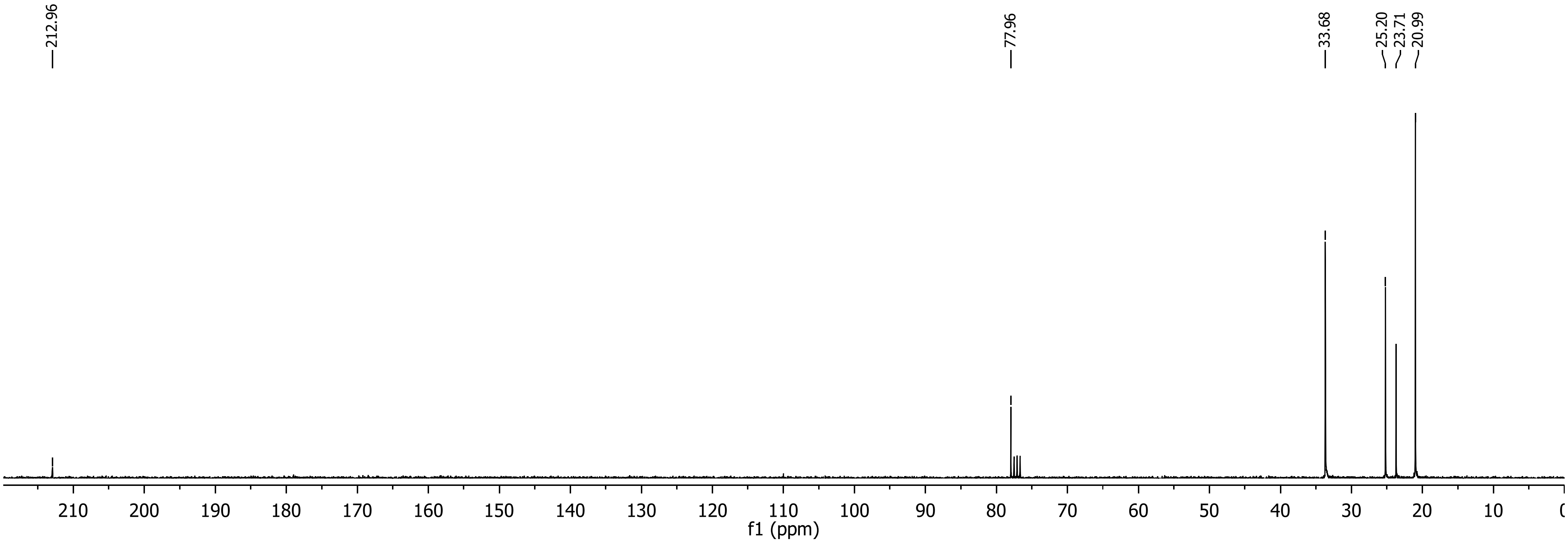
212.96
77.96
33.68
25.20
23.71
20.99
210 200 190 180 170 160 150 140 130 120 110 100 90 80 70 60 50 40 30 20 10
f1 (ppm)

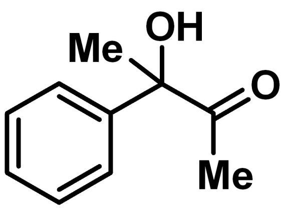
Me
OH
O
Me

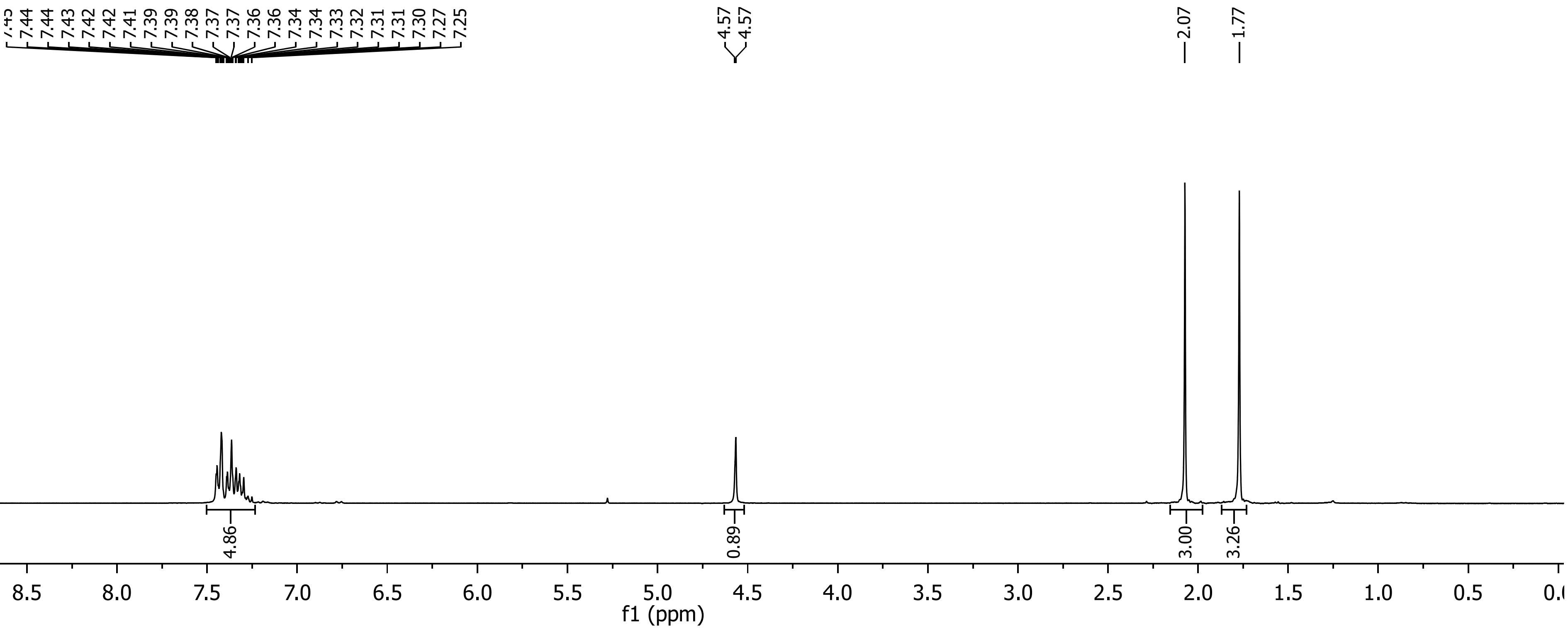
7.44 7.44 7.43 7.42 7.42 7.41 7.39 7.39 7.38 7.37 7.37 7.36 7.36 7.34 7.34 7.33 7.32 7.31 7.31 7.30 7.27 7.25
4.57 4.57
2.07
1.77
4.86
0.89
3.00
3.26
9.5 9.0 8.5 8.0 7.5 7.0 6.5 6.0 5.5 5.0 4.5 4.0 3.5 3.0 2.5 2.0 1.5 1.0 0.5
f1 (ppm)

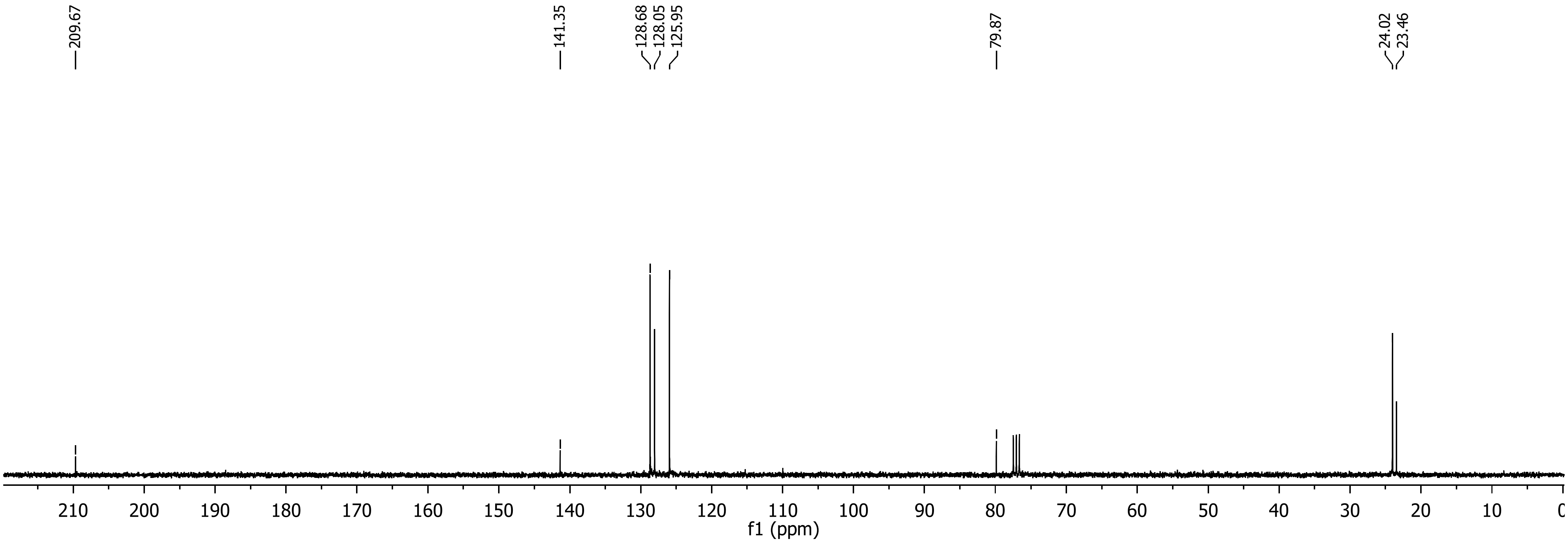
209.67
141.35
128.68
128.05
125.95
79.87
24.02
23.46
210 200 190 180 170 160 150 140 130 120 110 100 90 80 70 60 50 40 30 20 10
f1 (ppm)

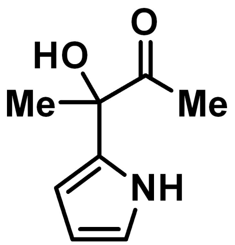
HO
O
Me
Me
NH

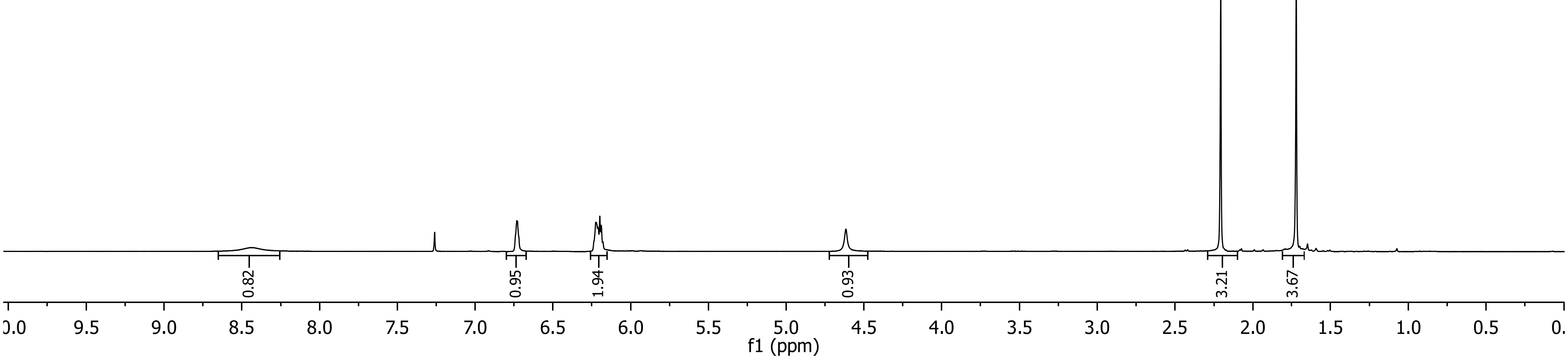
6.74
6.74
6.73
6.73
6.72
6.72
6.23
6.23
6.22
6.22
6.21
6.21
6.20
6.19
6.19
4.62
2.21
2.21
1.72
0.82
0.95
1.94
0.93
3.21
3.67
9.5
9.0
8.5
8.0
7.5
7.0
6.5
6.0
5.5
5.0
4.5
4.0
3.5
3.0
2.5
2.0
1.5
1.0
0.5
f1 (ppm)

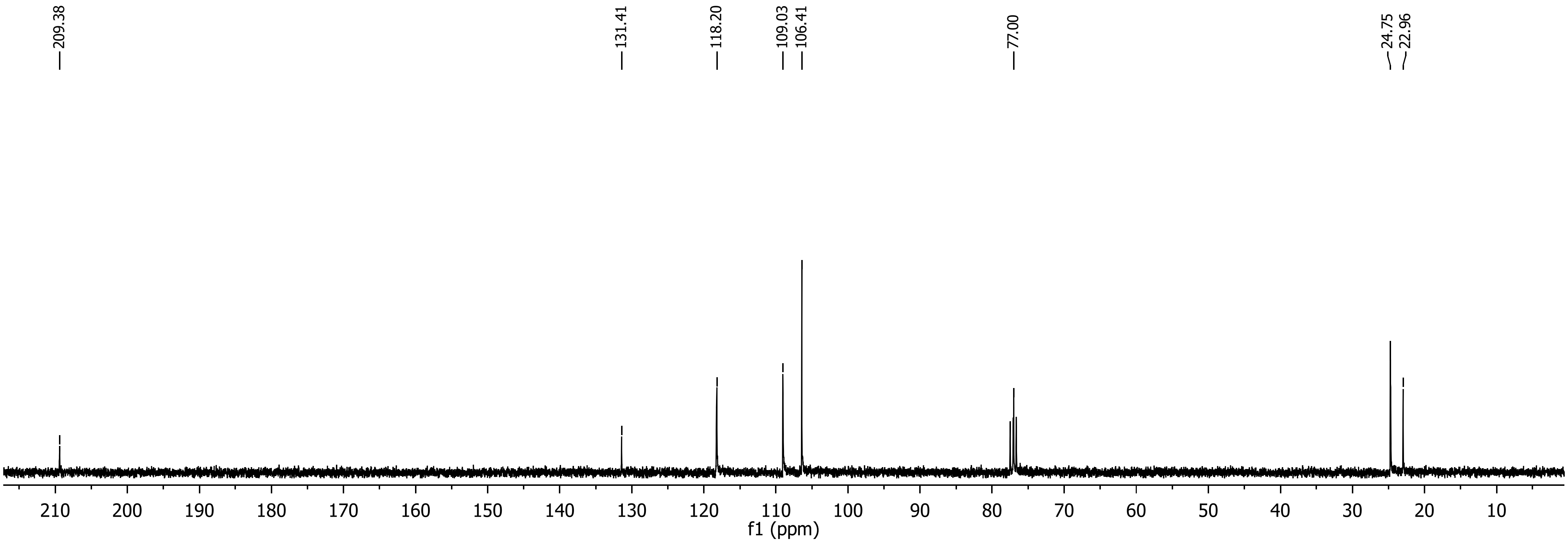
209.38
131.41
118.20
109.03
106.41
77.00
24.75
22.96
210
200
190
180
170
160
150
140
130
120
110
100
90
80
70
60
50
40
30
20
10
f1 (ppm)

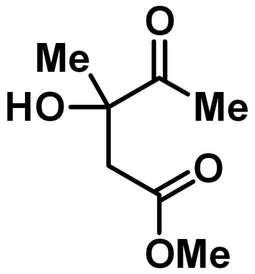
Me
HO
O
Me
O
OMe

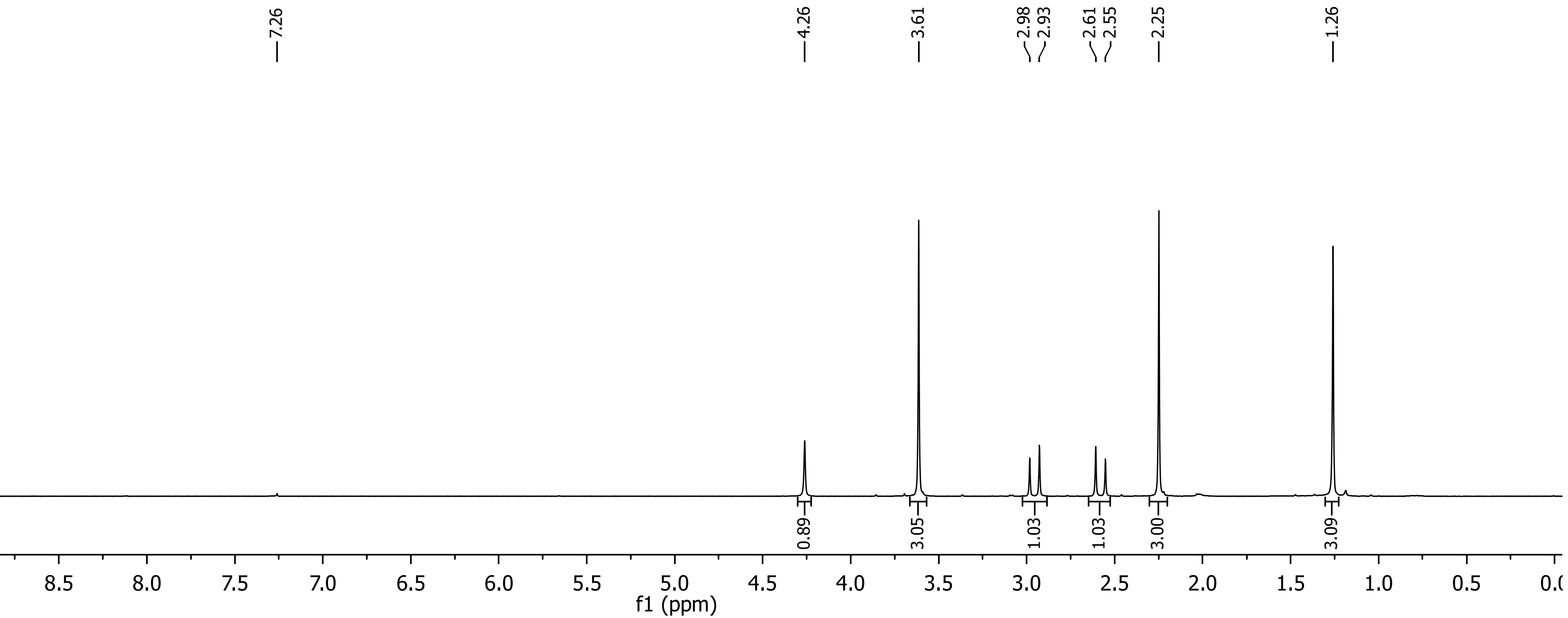
7.26
4.26
3.61
2.98
2.93
2.61
2.55
2.25
1.26
0.89
3.05
1.03
1.03
3.00
3.09
f1 (ppm)

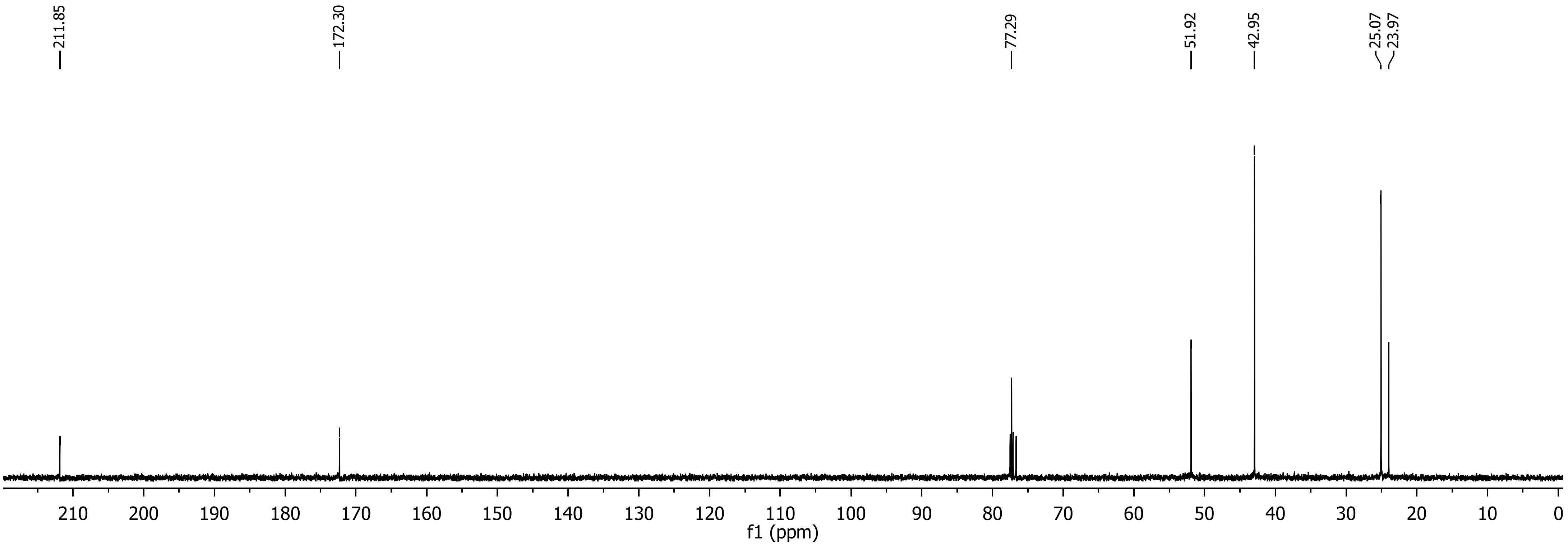
211.85
172.30
77.29
51.92
42.95
25.07
23.97
f1 (ppm)

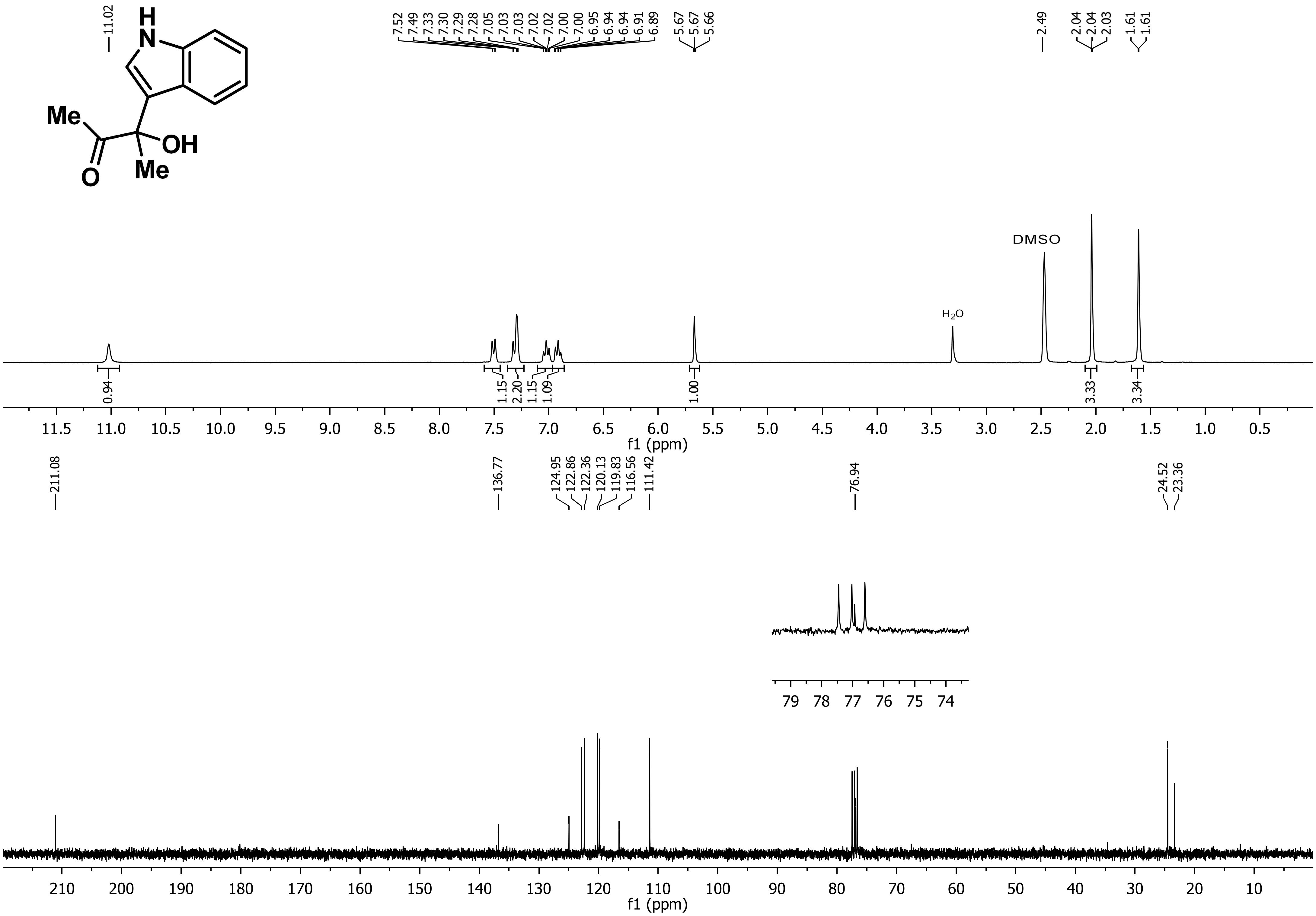
H
N
Me
OH
Me
O
11.02
7.52 7.49 7.33 7.30 7.29 7.28 7.05 7.03 7.03 7.02 7.02 7.00 7.00 6.95 6.94 6.94 6.91 6.89
5.67 5.67 5.66
2.49
2.04 2.04 2.03
1.61 1.61
DMSO
H2O
0.94
1.15 2.20 1.15 1.09
1.00
3.33
3.34
11.5 11.0 10.5 10.0 9.5 9.0 8.5 8.0 7.5 7.0 6.5 6.0 5.5 5.0 4.5 4.0 3.5 3.0 2.5 2.0 1.5 1.0 0.5
f1 (ppm)
211.08
136.77
124.95 122.86 122.36 120.13 119.83 116.56 111.42
76.94
24.52 23.36
79 78 77 76 75 74
210 200 190 180 170 160 150 140 130 120 110 100 90 80 70 60 50 40 30 20 10
f1 (ppm)

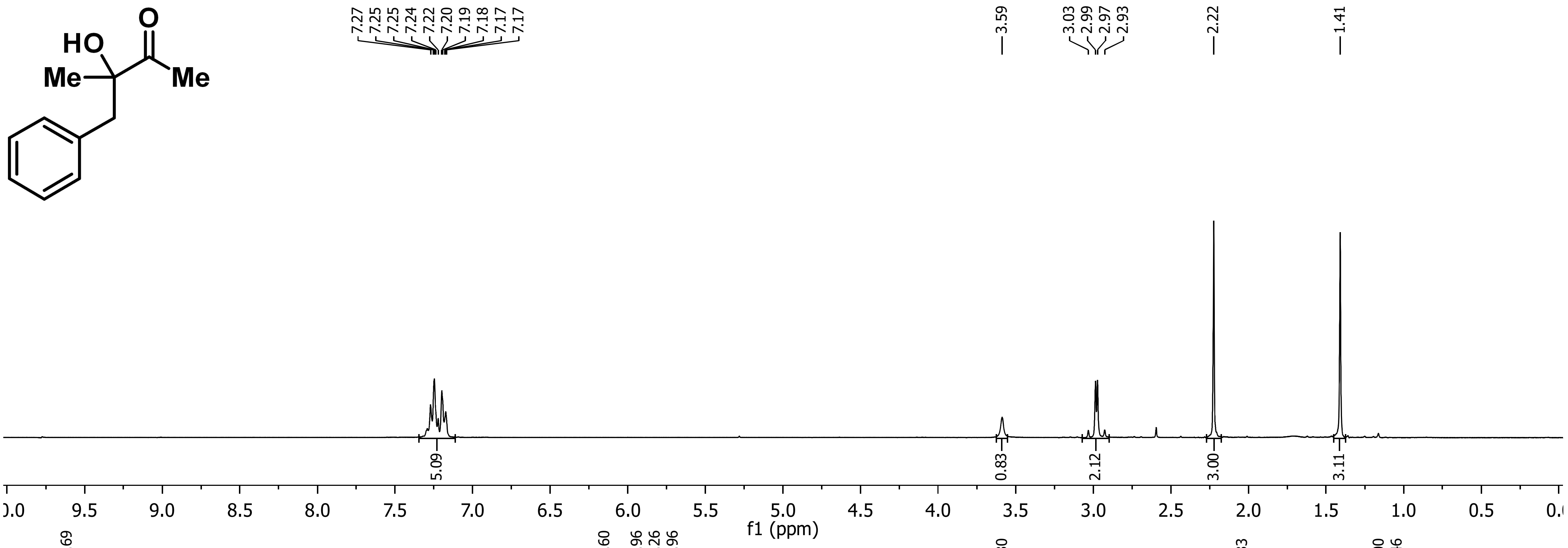
HO
O
Me
Me
7.27 7.25 7.25 7.24 7.22 7.20 7.19 7.18 7.17 7.17
3.59
3.03 2.99 2.97 2.93
2.22
1.41
5.09
0.83
2.12
3.00
3.11
f1 (ppm)

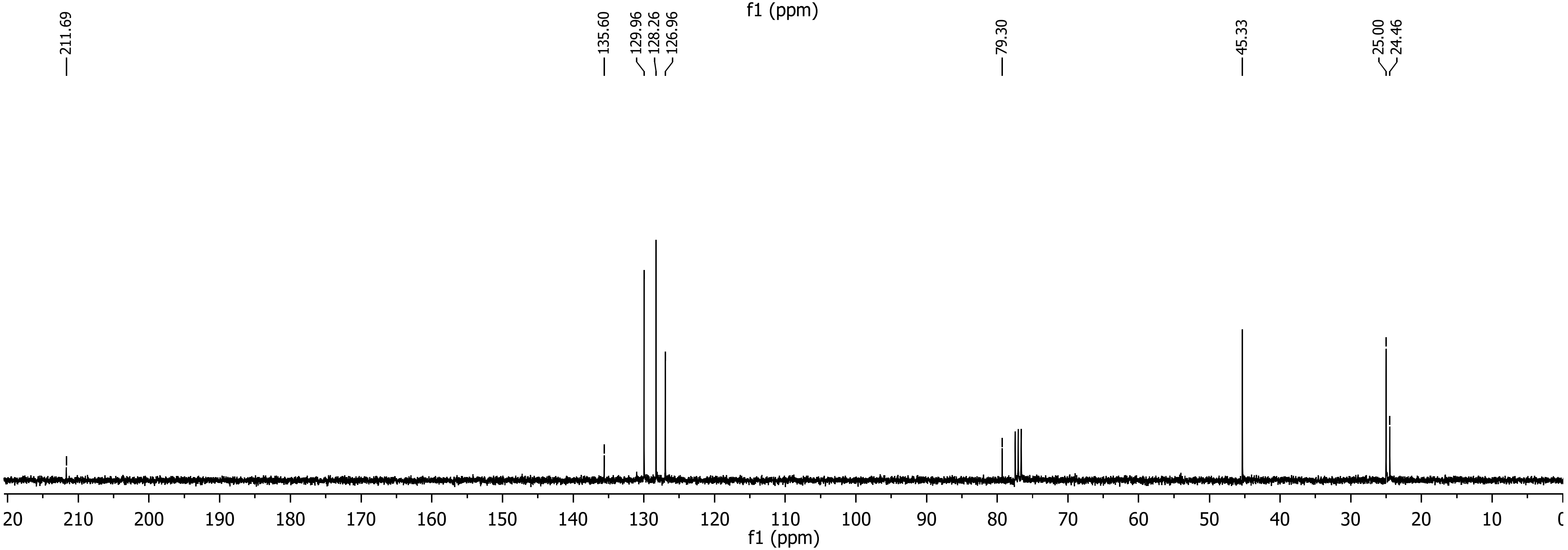
211.69
135.60
129.96
128.26
126.96
79.30
45.33
25.00
24.46
f1 (ppm)

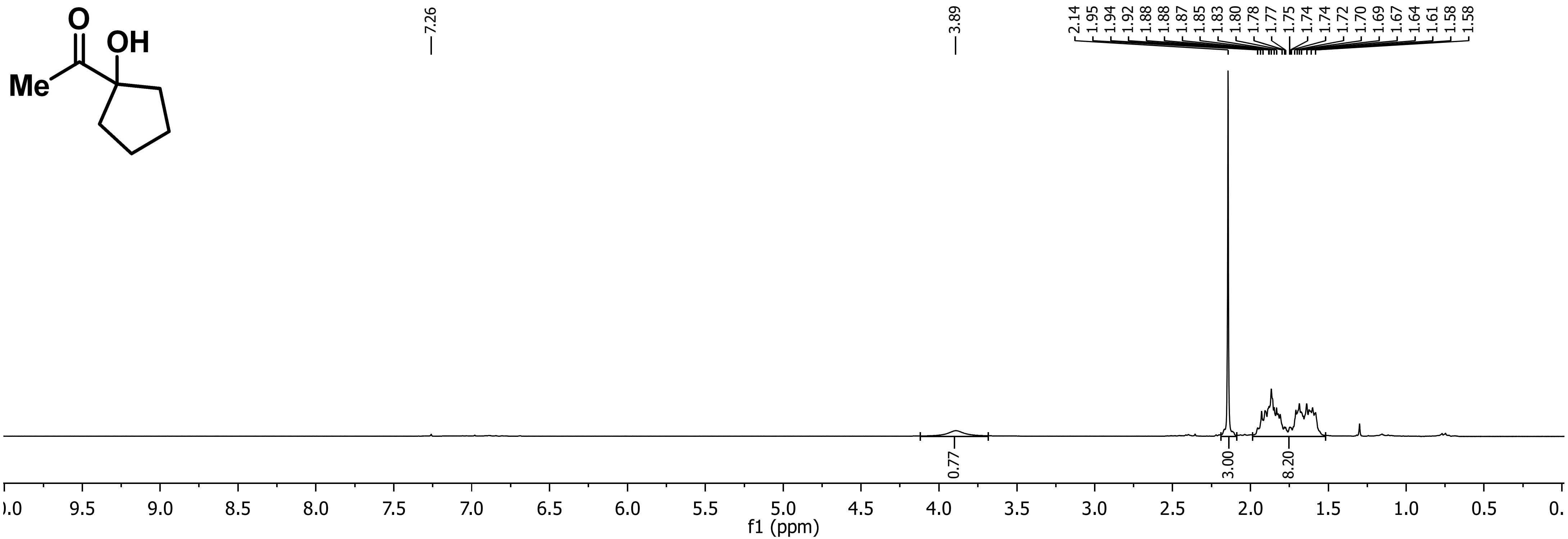
O
OH
Me
7.26
3.89
2.14 1.95 1.94 1.92 1.88 1.88 1.87 1.85 1.83 1.80 1.78 1.77 1.75 1.74 1.74 1.72 1.70 1.69 1.67 1.64 1.61 1.58 1.58
0.77
3.00
8.20
9.5 9.0 8.5 8.0 7.5 7.0 6.5 6.0 5.5 5.0 4.5 4.0 3.5 3.0 2.5 2.0 1.5 1.0 0.5
f1 (ppm)

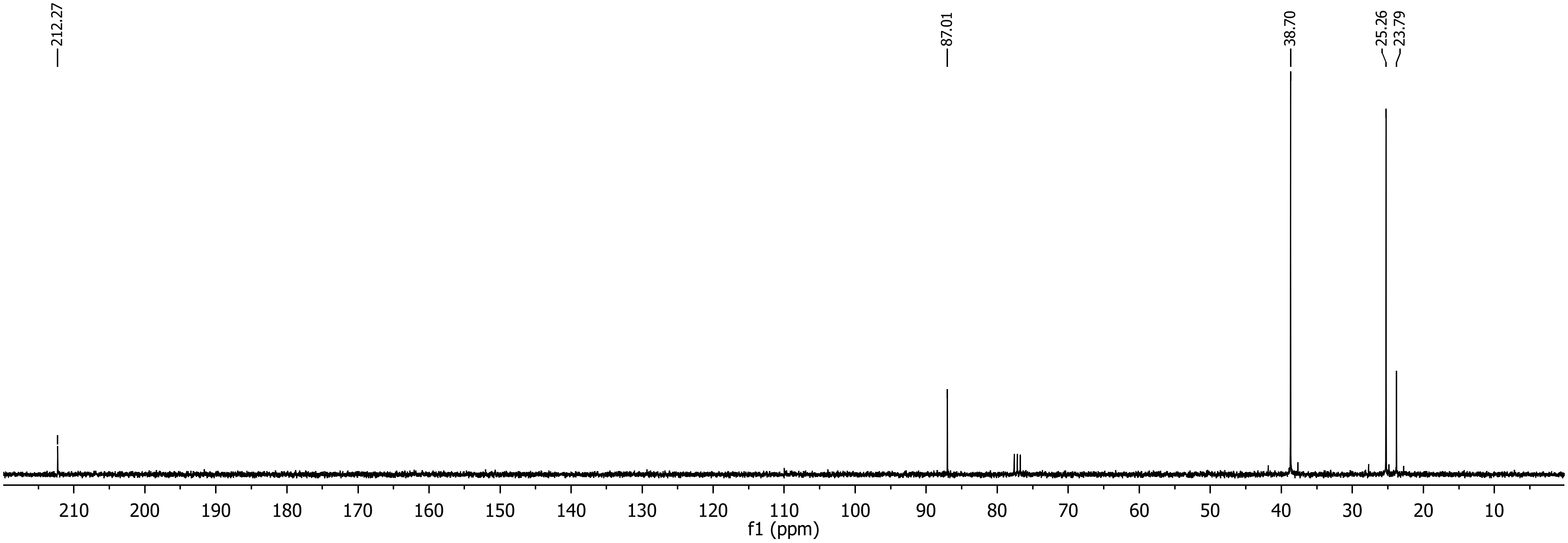
212.27
87.01
38.70
25.26
23.79
210 200 190 180 170 160 150 140 130 120 110 100 90 80 70 60 50 40 30 20 10
f1 (ppm)

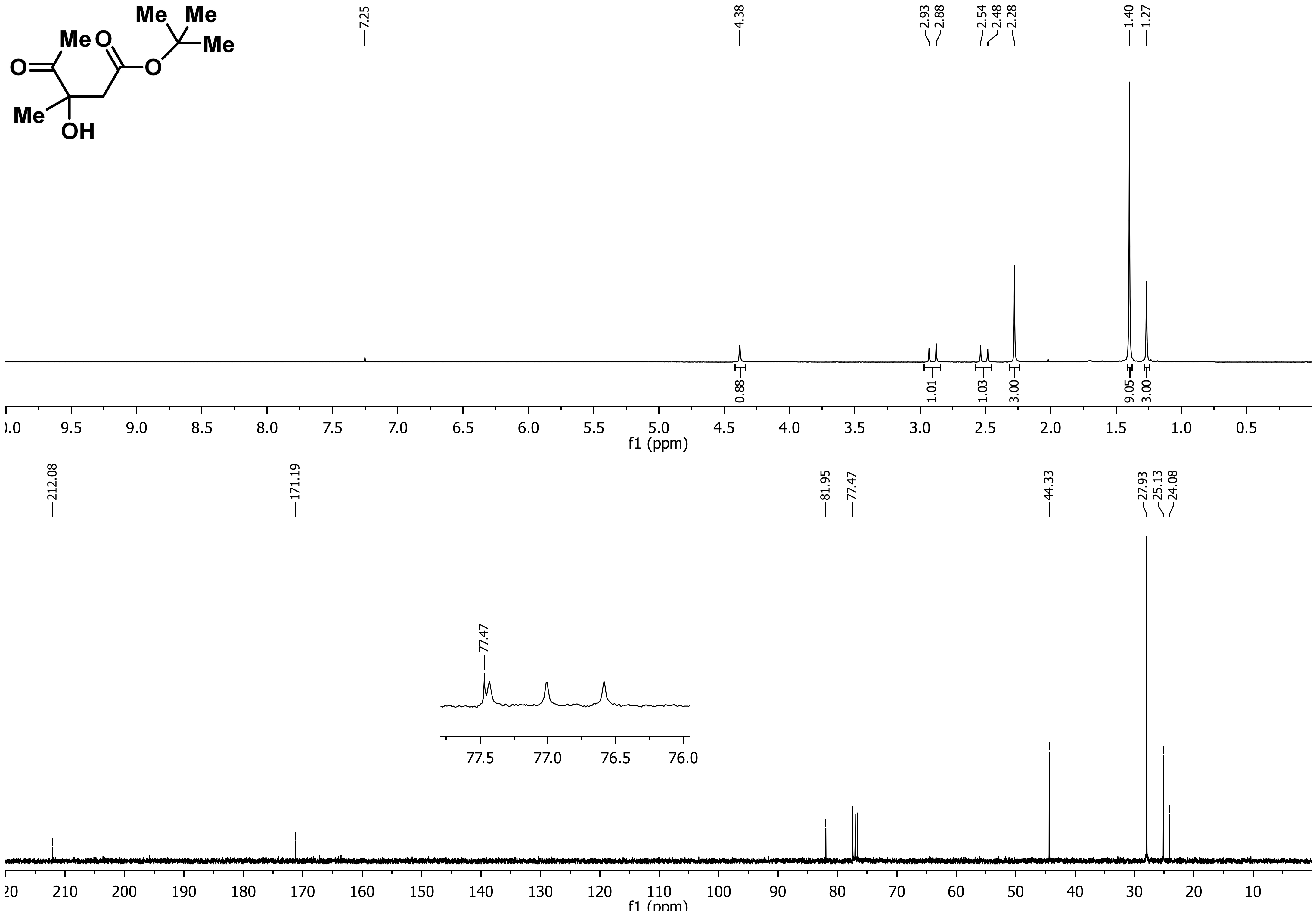

Me
Me
Me
Me
O
O
O
Me
OH
7.25
4.38
2.93
2.88
2.54
2.48
2.28
1.40
1.27
0.88
1.01
1.03
3.00
9.05
3.00
f1 (ppm)
212.08
171.19
81.95
77.47
44.33
27.93
25.13
24.08
77.47
77.5
77.0
76.5
76.0
f1 (ppm)

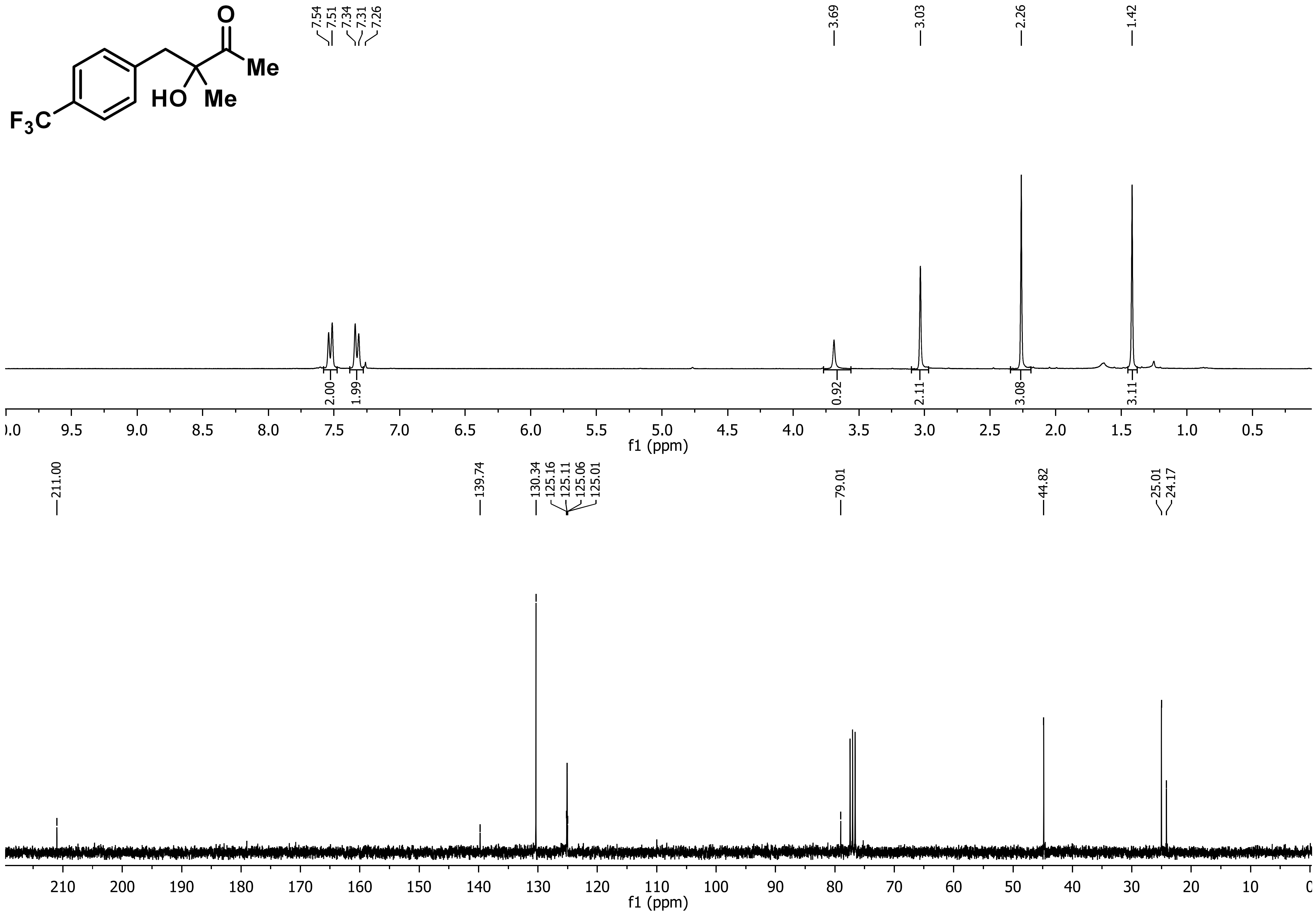
O
Me
HO
Me
F3C
7.54
7.51
7.34
7.31
7.26
3.69
3.03
2.26
1.42
2.00
1.99
0.92
2.11
3.08
3.11
f1 (ppm)
211.00
139.74
130.34
125.16
125.11
125.06
125.01
79.01
44.82
25.01
24.17
f1 (ppm)

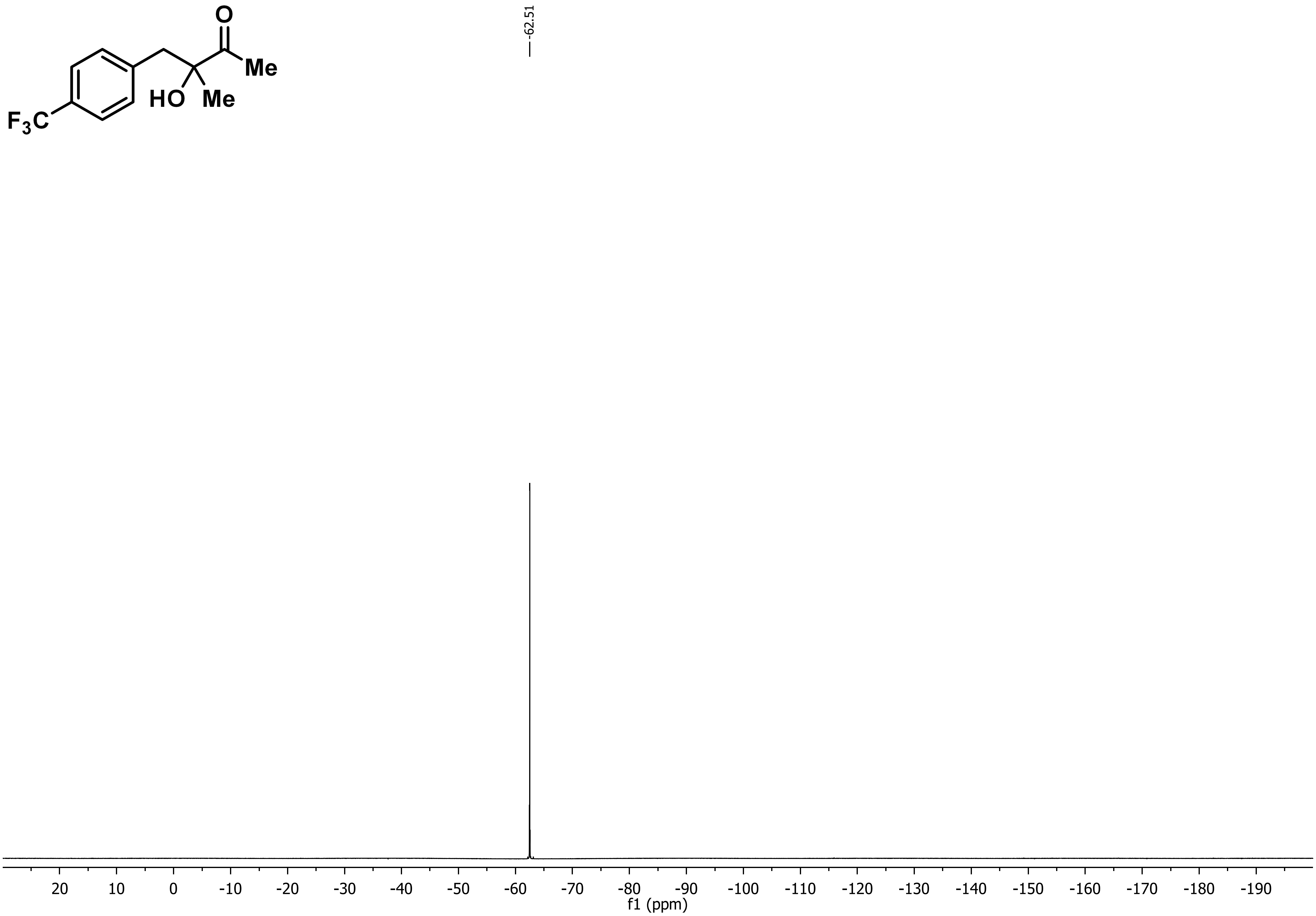

O
Me
HO
Me
F_3C
-62.51
20
10
0
-10
-20
-30
-40
-50
-60
-70
-80
-90
-100
-110
-120
-130
-140
-150
-160
-170
-180
-190
f1 (ppm)

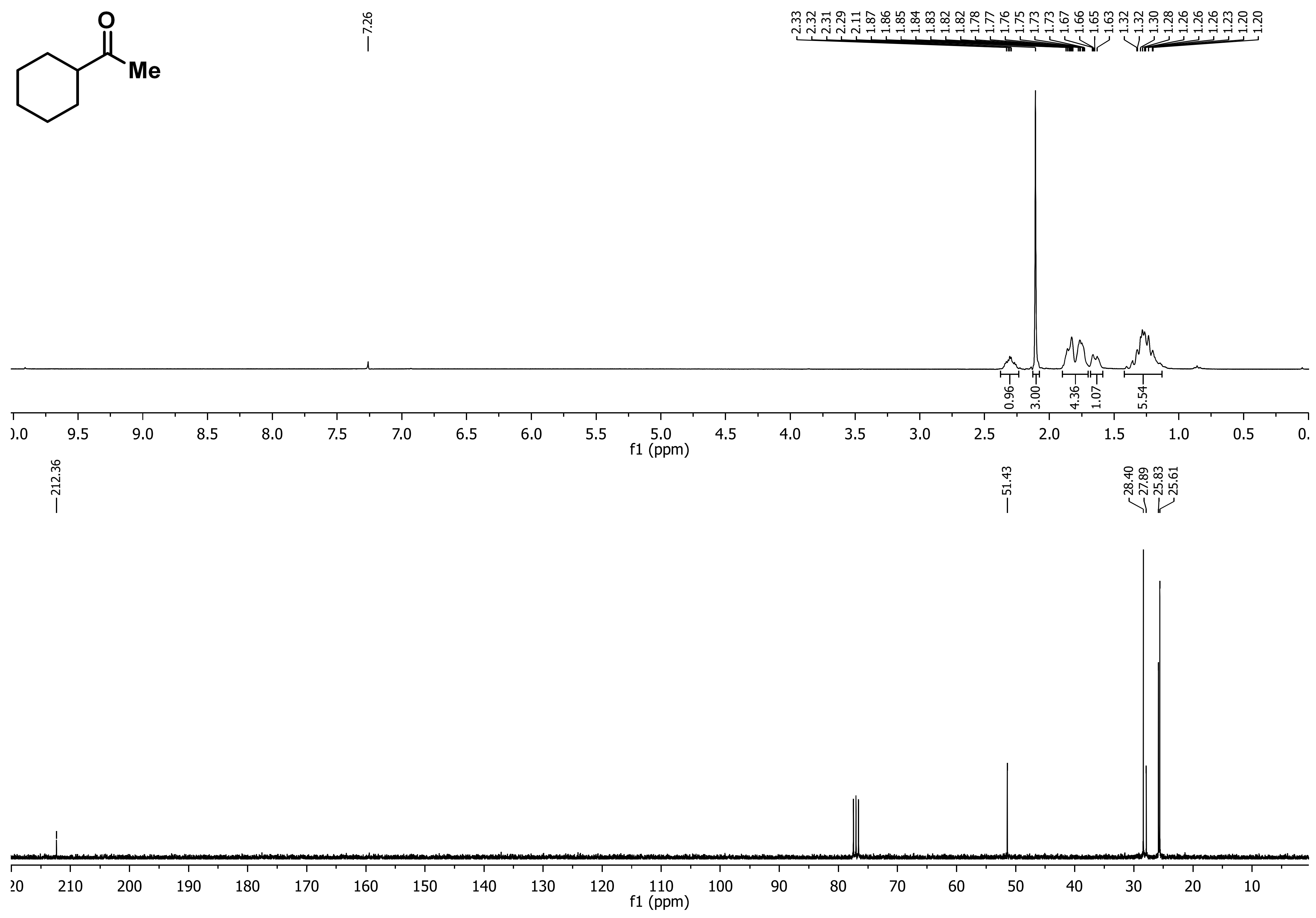

O
Me
7.26
2.33 2.32 2.31 2.29 2.11 1.87 1.86 1.85 1.84 1.83 1.82 1.82 1.78 1.77 1.76 1.75 1.73 1.73 1.67 1.66 1.65 1.63 1.32 1.32 1.30 1.28 1.26 1.26 1.26 1.23 1.20 1.20
0.96 3.00 4.36 1.07 5.54
f1 (ppm)
212.36
51.43
28.40 27.89 25.83 25.61
f1 (ppm)

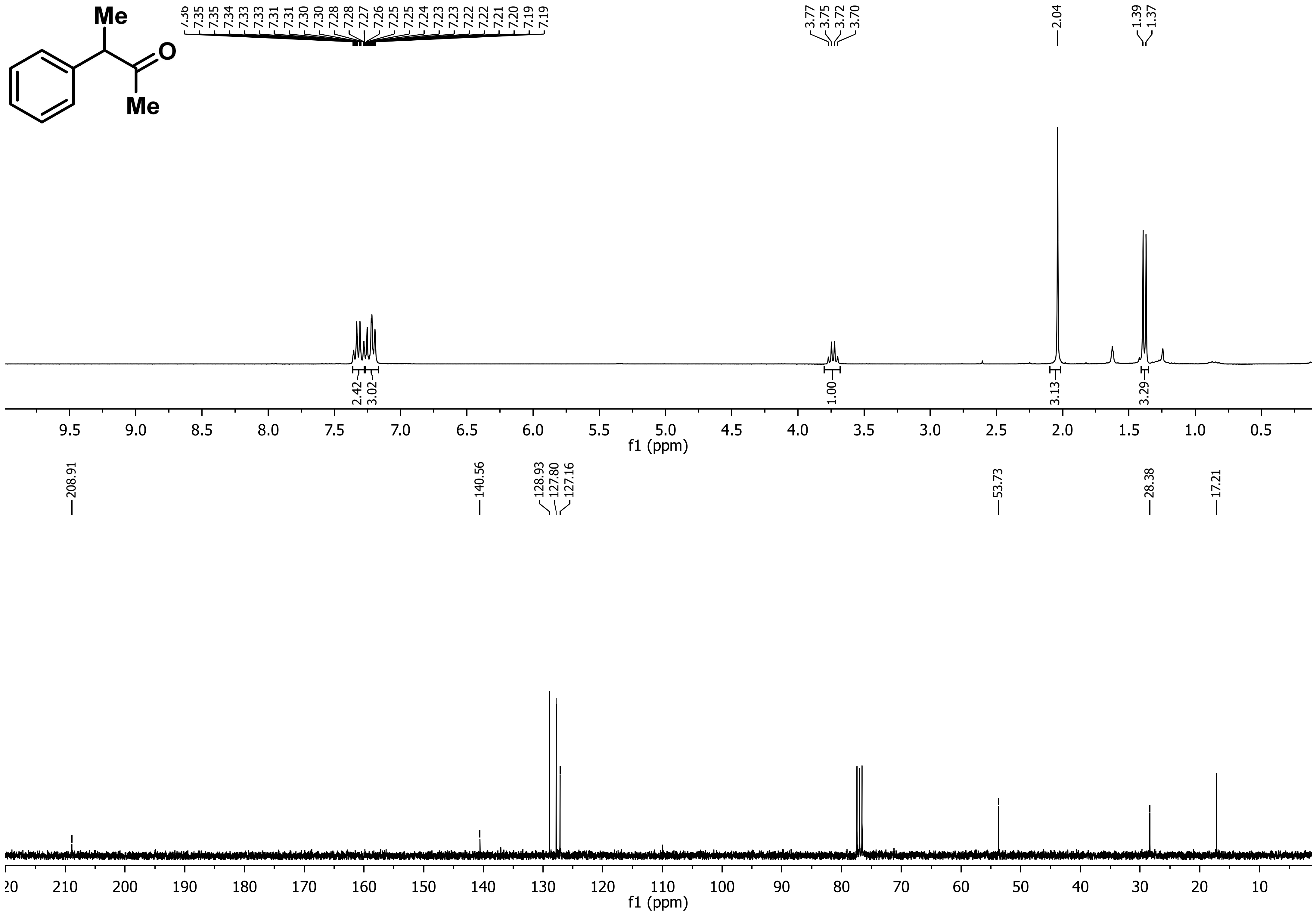

Me
O
Me
7.35 7.35 7.34 7.33 7.33 7.31 7.31 7.30 7.30 7.28 7.28 7.27 7.26 7.25 7.25 7.24 7.23 7.23 7.22 7.22 7.21 7.20 7.19 7.19
3.77 3.75 3.72 3.70
2.04
1.39 1.37
2.42 3.02 1.00 3.13 3.29
f1 (ppm)
208.91
140.56
128.93 127.80 127.16
53.73
28.38
17.21
f1 (ppm)

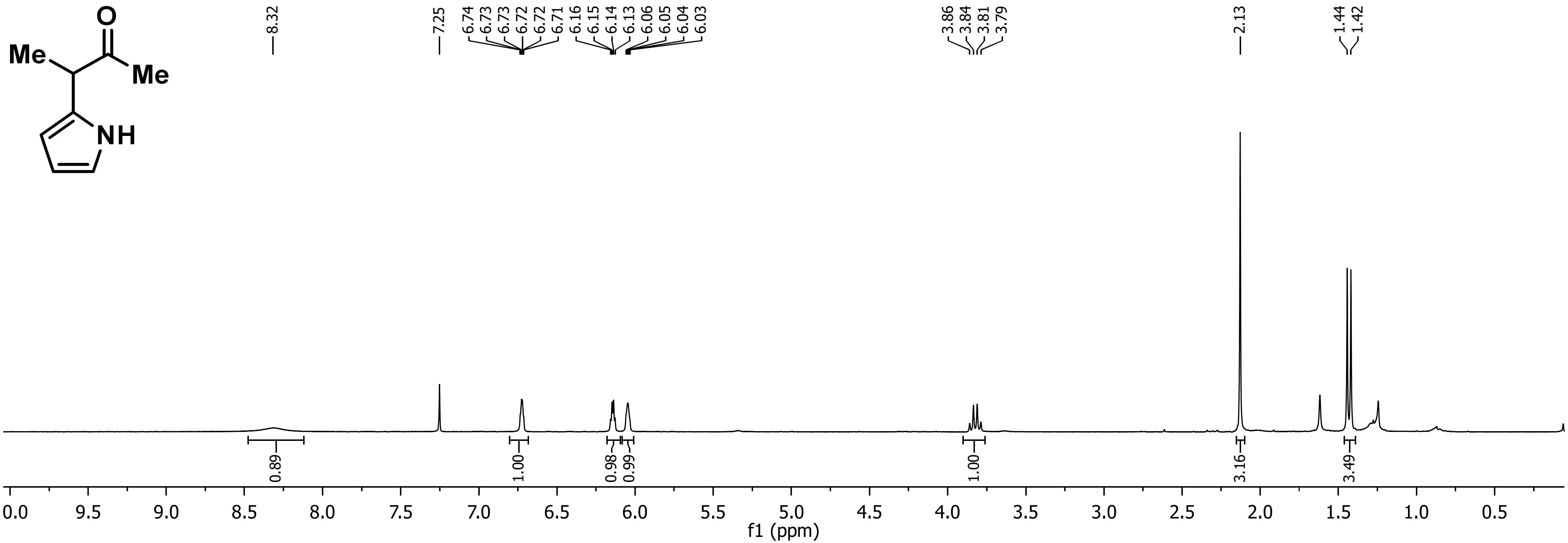
O
Me
Me
NH
8.32
7.25
6.74
6.73
6.73
6.72
6.72
6.71
6.16
6.15
6.14
6.13
6.06
6.05
6.04
6.03
3.86
3.84
3.81
3.79
2.13
1.44
1.42
0.89
1.00
0.98
0.99
1.00
3.16
3.49
f1 (ppm)

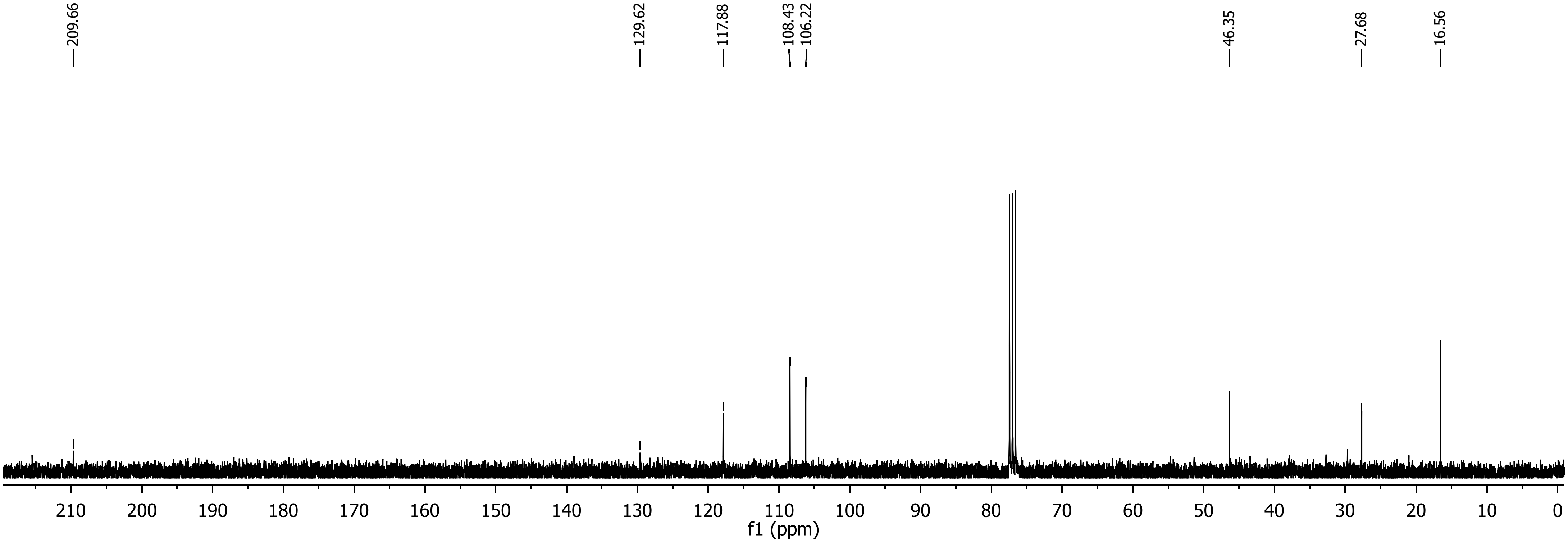
209.66
129.62
117.88
108.43
106.22
46.35
27.68
16.56
f1 (ppm)

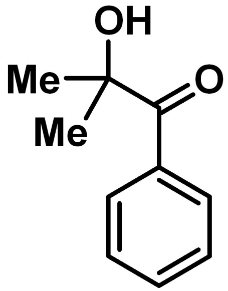
OH
Me
O
Me

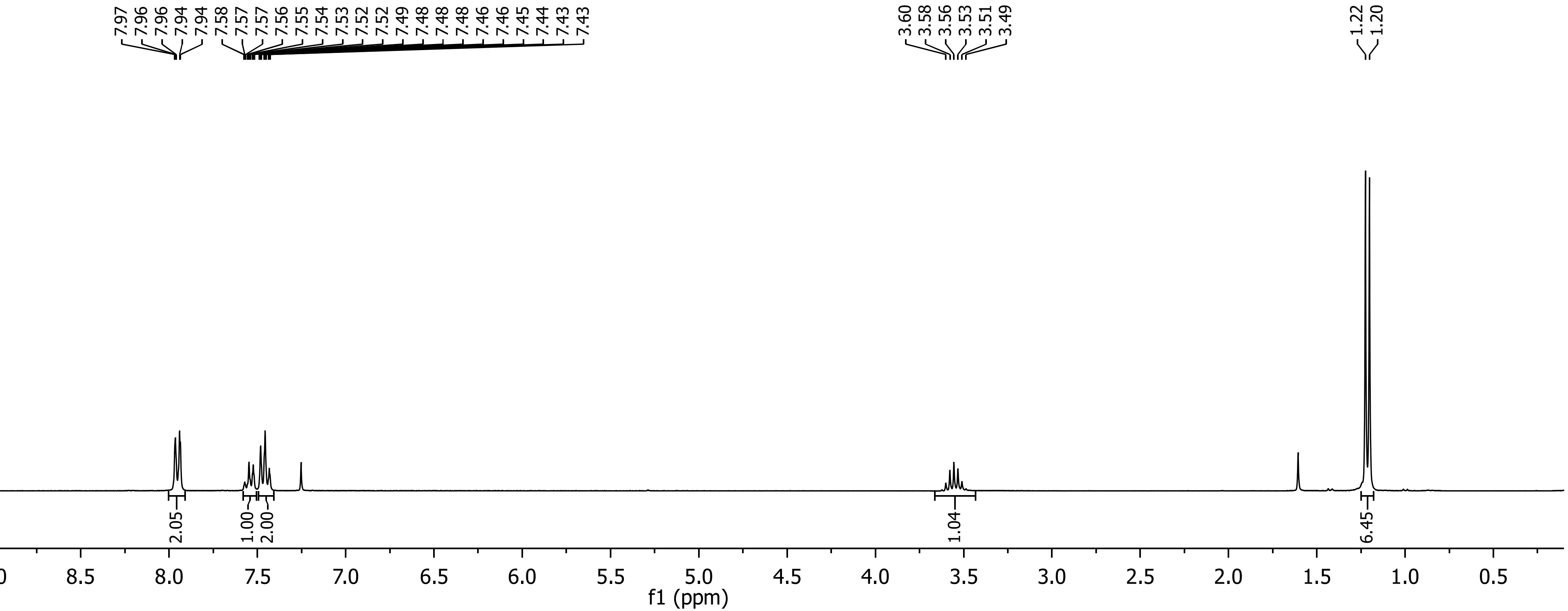
7.97
7.96
7.96
7.94
7.94
7.58
7.57
7.57
7.56
7.55
7.54
7.53
7.52
7.52
7.49
7.48
7.48
7.48
7.46
7.46
7.45
7.44
7.43
7.43
3.60
3.58
3.56
3.53
3.51
3.49
1.22
1.20
2.05
1.00
2.00
1.04
6.45
9.5
9.0
8.5
8.0
7.5
7.0
6.5
6.0
5.5
5.0
4.5
4.0
3.5
3.0
2.5
2.0
1.5
1.0
0.5
f1 (ppm)

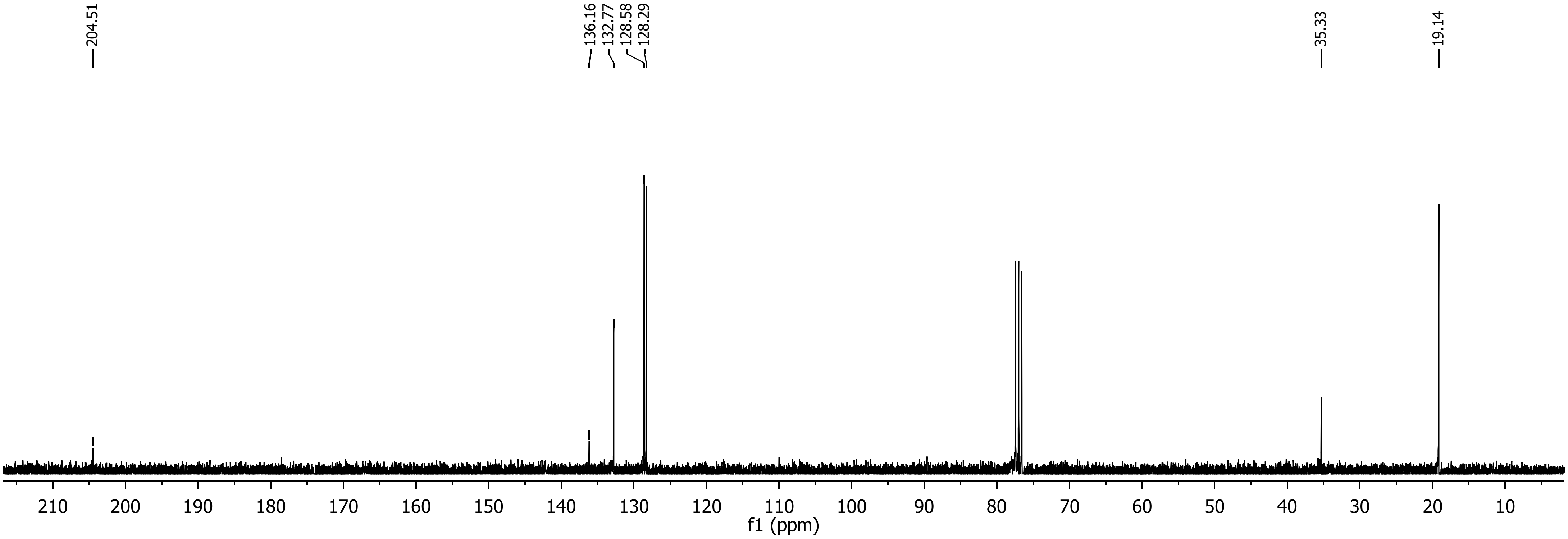
204.51
136.16
132.77
128.58
128.29
35.33
19.14
210
200
190
180
170
160
150
140
130
120
110
100
90
80
70
60
50
40
30
20
10
f1 (ppm)

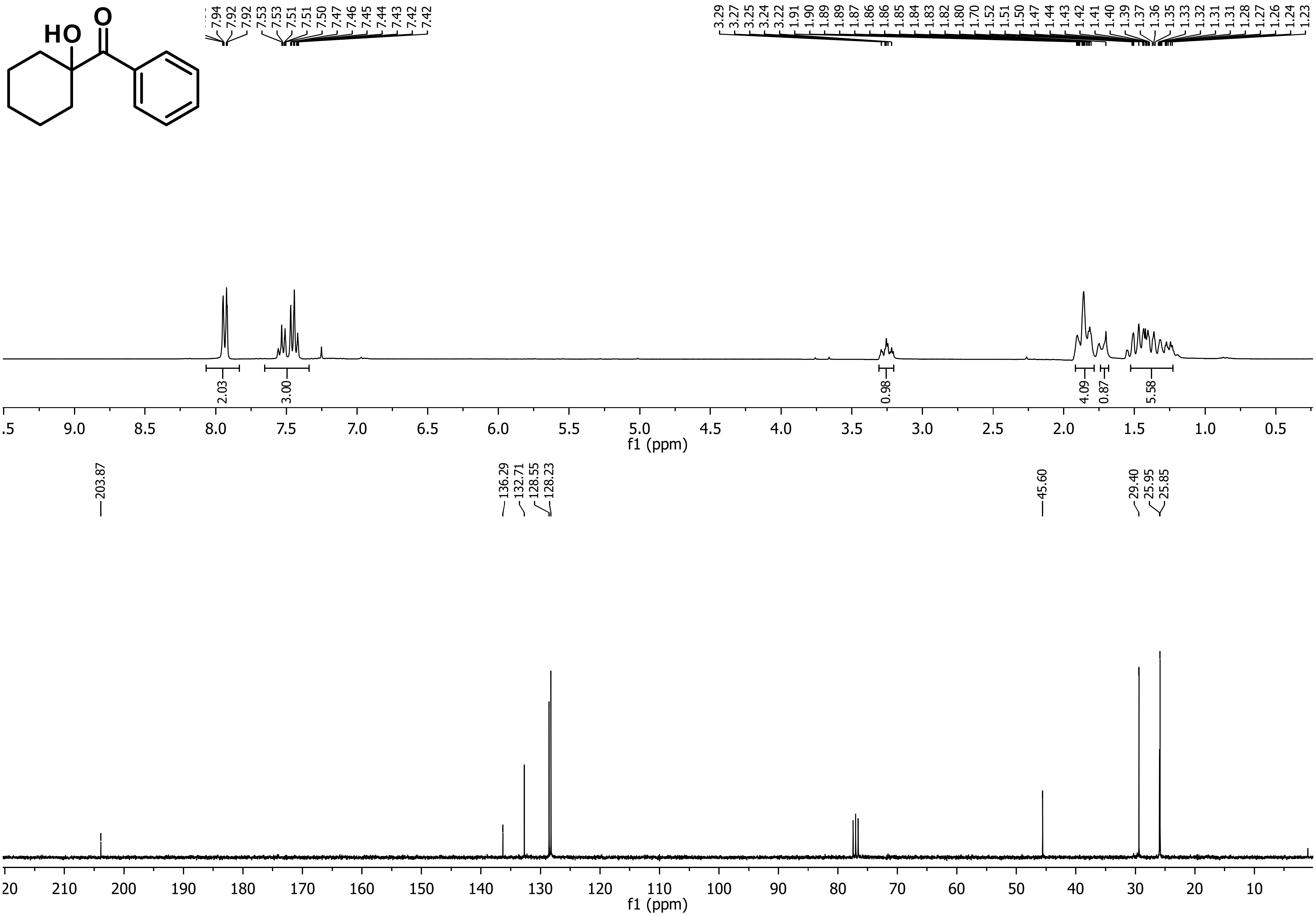
HO
O
7.94
7.92
7.92
7.53
7.53
7.51
7.51
7.50
7.47
7.46
7.45
7.44
7.43
7.42
7.42
3.29
3.27
3.25
3.24
3.22
1.91
1.90
1.89
1.89
1.87
1.86
1.86
1.85
1.84
1.83
1.82
1.80
1.70
1.52
1.51
1.50
1.47
1.44
1.43
1.42
1.41
1.40
1.39
1.37
1.36
1.35
1.33
1.32
1.31
1.31
1.28
1.27
1.26
1.24
1.23
2.03
3.00
0.98
4.09
0.87
5.58
9.0
8.5
8.0
7.5
7.0
6.5
6.0
5.5
5.0
4.5
4.0
3.5
3.0
2.5
2.0
1.5
1.0
0.5
f1 (ppm)
203.87
136.29
132.71
128.55
128.23
45.60
29.40
25.95
25.85
210
200
190
180
170
160
150
140
130
120
110
100
90
80
70
60
50
40
30
20
10
f1 (ppm)

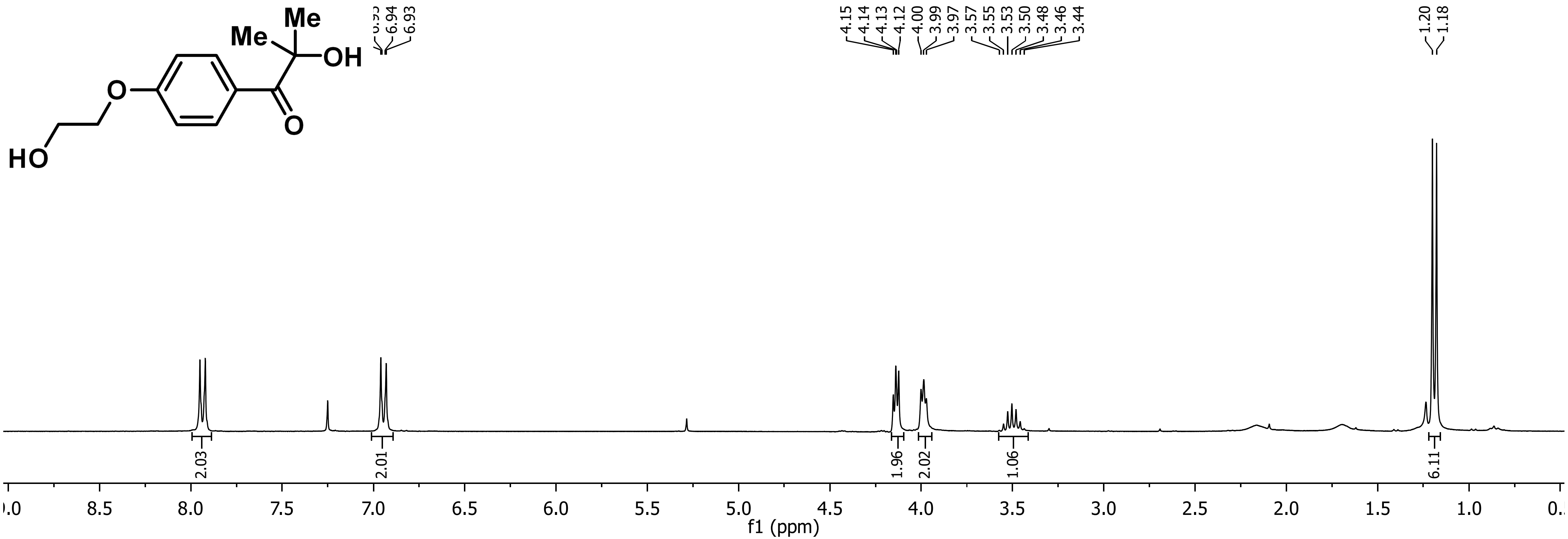
Me
Me
OH
O
O
HO
6.94
6.93
4.15
4.14
4.13
4.12
4.00
3.99
3.97
3.57
3.55
3.53
3.50
3.48
3.46
3.44
1.20
1.18
2.03
2.01
1.96
2.02
1.06
6.11
8.5
8.0
7.5
7.0
6.5
6.0
5.5
5.0
4.5
4.0
3.5
3.0
2.5
2.0
1.5
1.0
f1 (ppm)

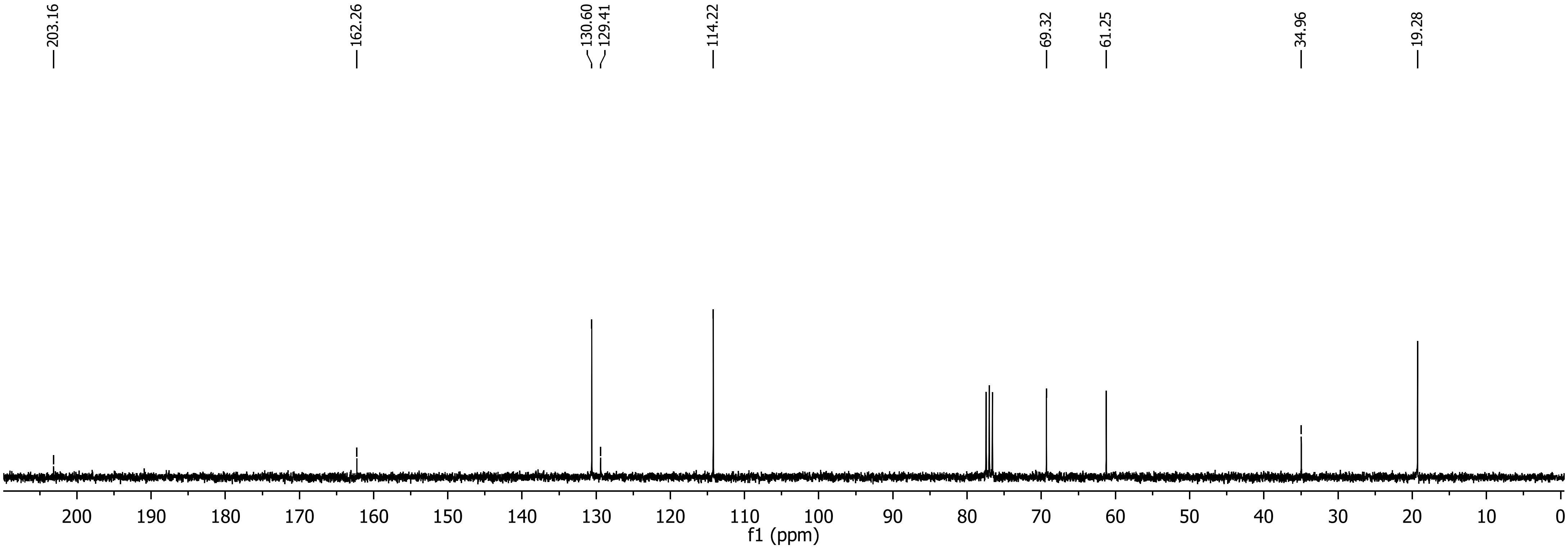
203.16
162.26
130.60
129.41
114.22
69.32
61.25
34.96
19.28
200
190
180
170
160
150
140
130
120
110
100
90
80
70
60
50
40
30
20
10
0
f1 (ppm)

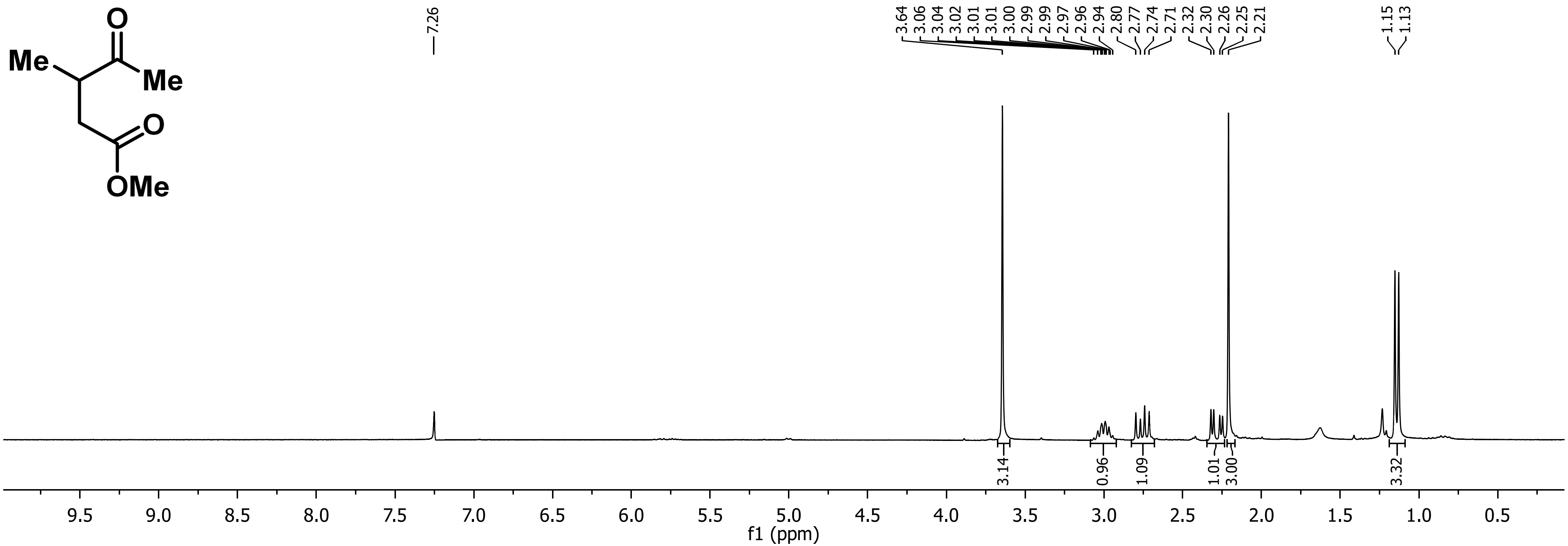
O
Me
Me
O
OMe
7.26
3.64
3.06
3.04
3.02
3.01
3.01
3.00
2.99
2.99
2.97
2.96
2.94
2.80
2.77
2.74
2.71
2.32
2.30
2.26
2.25
2.21
1.15
1.13
3.14
0.96
1.09
1.01
3.00
3.32
9.5 9.0 8.5 8.0 7.5 7.0 6.5 6.0 5.5 5.0 4.5 4.0 3.5 3.0 2.5 2.0 1.5 1.0 0.5
f1 (ppm)

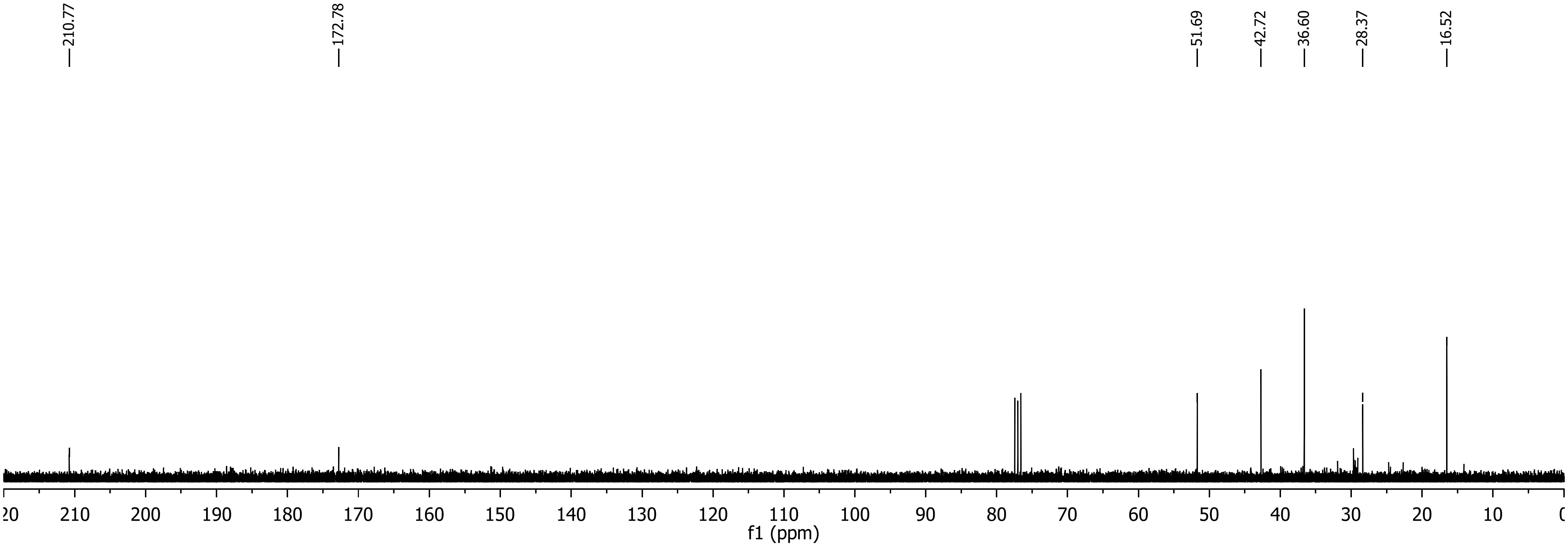
210.77
172.78
51.69
42.72
36.60
28.37
16.52
210 200 190 180 170 160 150 140 130 120 110 100 90 80 70 60 50 40 30 20 10
f1 (ppm)

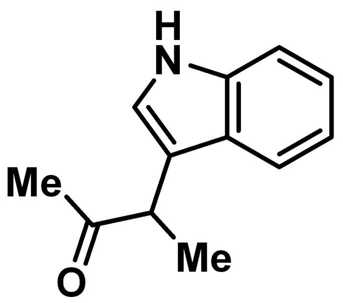
H
N
Me
Me
O

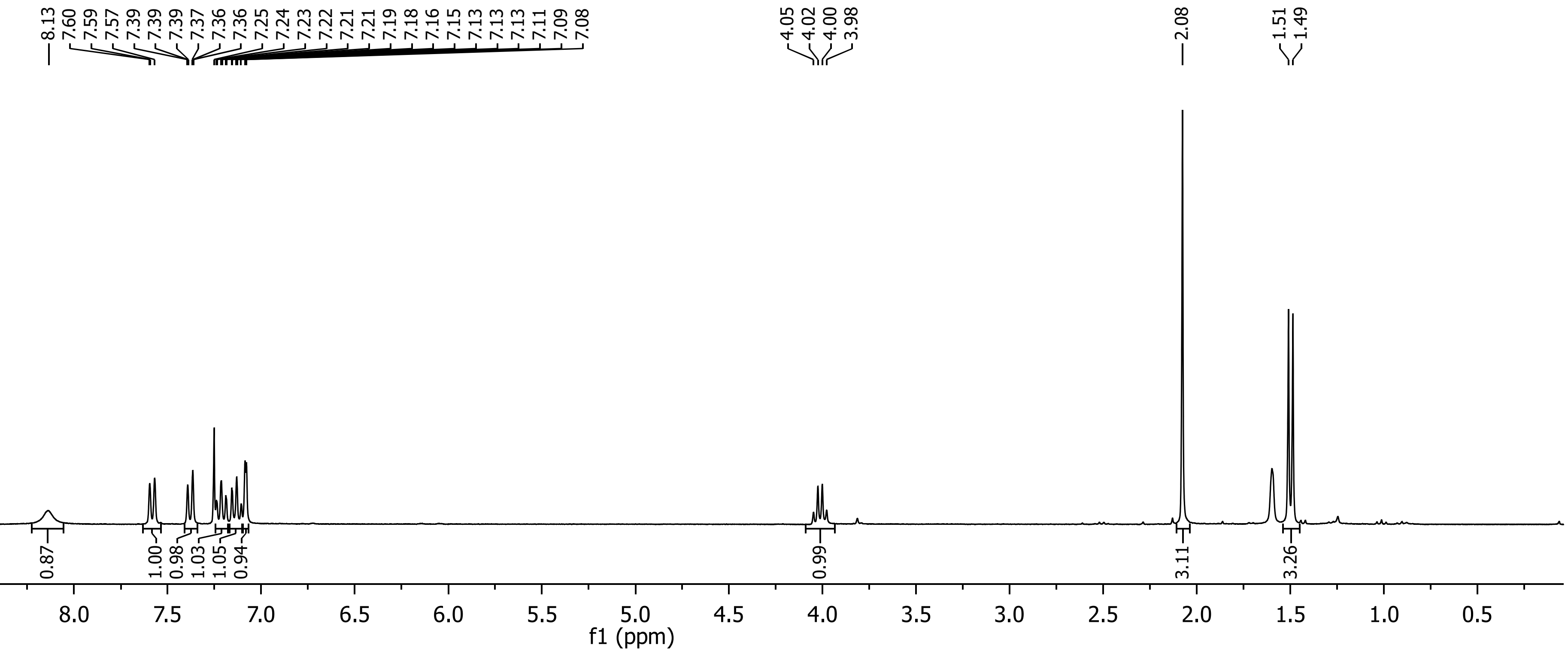
8.13
7.60
7.59
7.57
7.39
7.39
7.39
7.37
7.36
7.36
7.25
7.24
7.23
7.22
7.21
7.21
7.19
7.18
7.16
7.15
7.13
7.13
7.13
7.11
7.09
7.08
4.05
4.02
4.00
3.98
2.08
1.51
1.49
0.87
1.00
0.98
1.03
1.05
0.94
0.99
3.11
3.26
9.5
9.0
8.5
8.0
7.5
7.0
6.5
6.0
5.5
5.0
4.5
4.0
3.5
3.0
2.5
2.0
1.5
1.0
0.5
f1 (ppm)

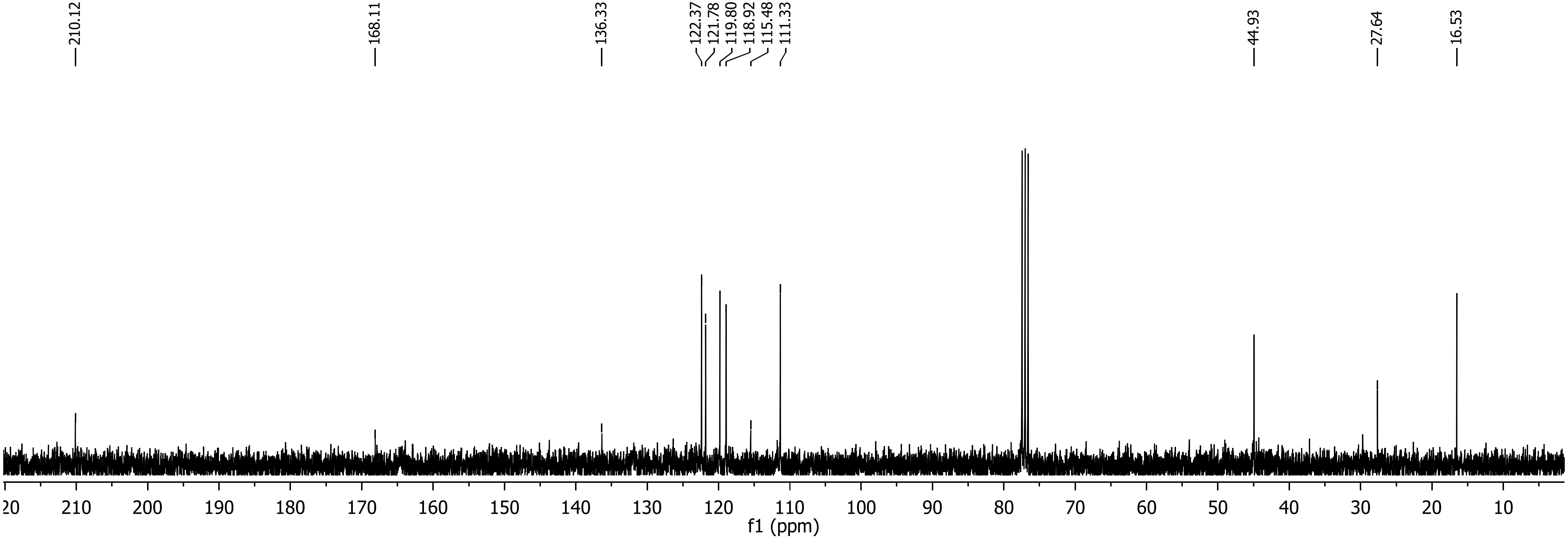
210.12
168.11
136.33
122.37
121.78
119.80
118.92
115.48
111.33
44.93
27.64
16.53
20
210
200
190
180
170
160
150
140
130
120
110
100
90
80
70
60
50
40
30
20
10
f1 (ppm)

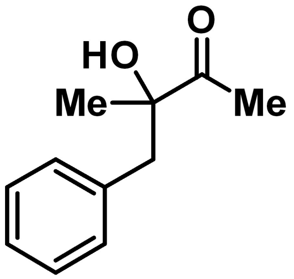
HO
O
Me
Me

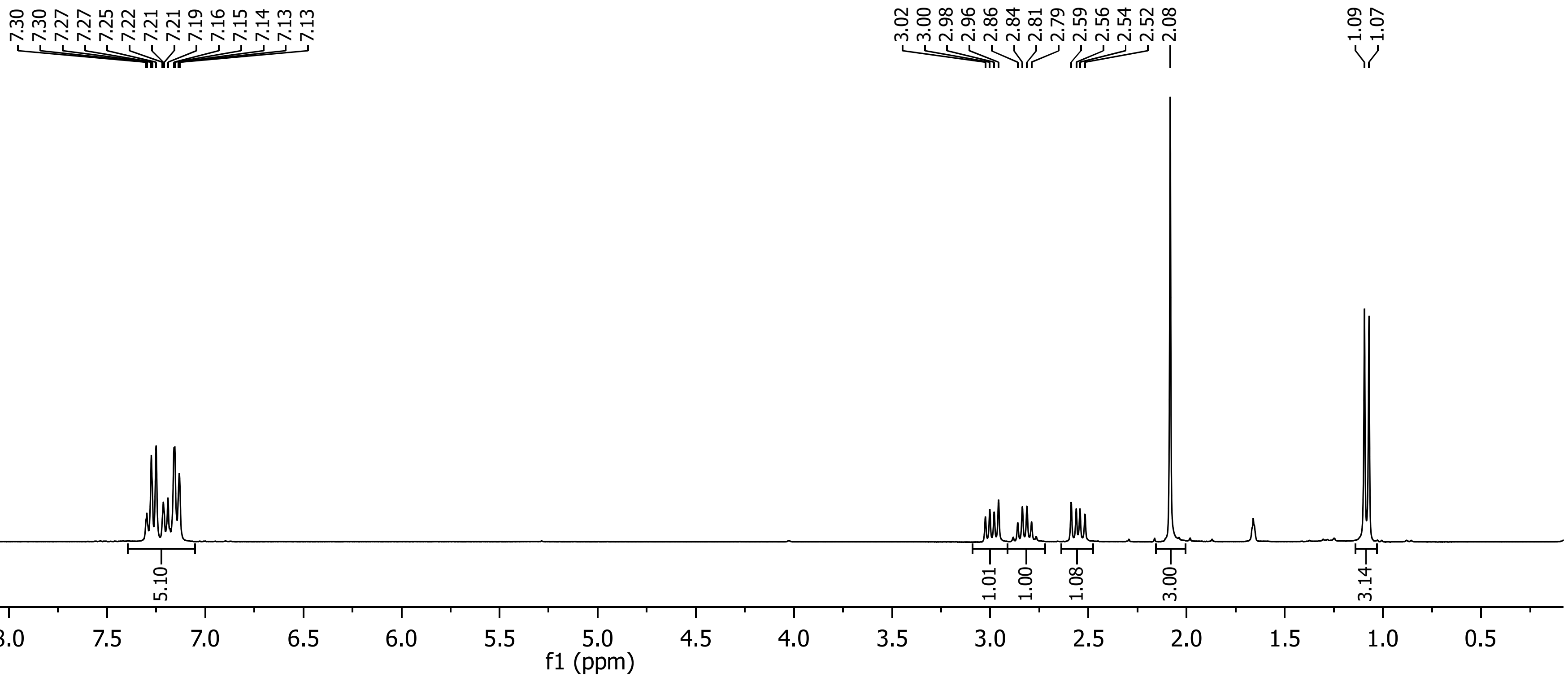
7.30
7.30
7.27
7.27
7.25
7.22
7.21
7.21
7.19
7.16
7.15
7.14
7.13
7.13
3.02
3.00
2.98
2.96
2.86
2.84
2.81
2.79
2.59
2.56
2.54
2.52
2.08
1.09
1.07
5.10
1.01
1.00
1.08
3.00
3.14
9.5 9.0 8.5 8.0 7.5 7.0 6.5 6.0 5.5 5.0 4.5 4.0 3.5 3.0 2.5 2.0 1.5 1.0 0.5
f1 (ppm)

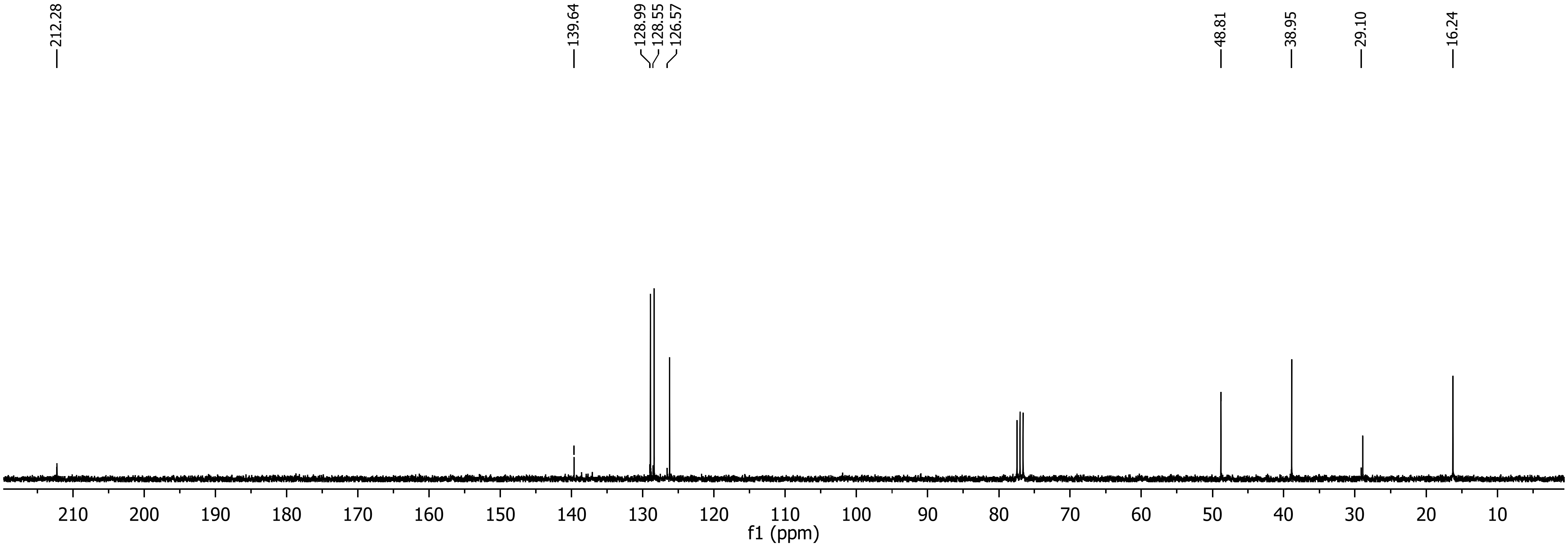
212.28
139.64
128.99
128.55
126.57
48.81
38.95
29.10
16.24
210 200 190 180 170 160 150 140 130 120 110 100 90 80 70 60 50 40 30 20 10
f1 (ppm)

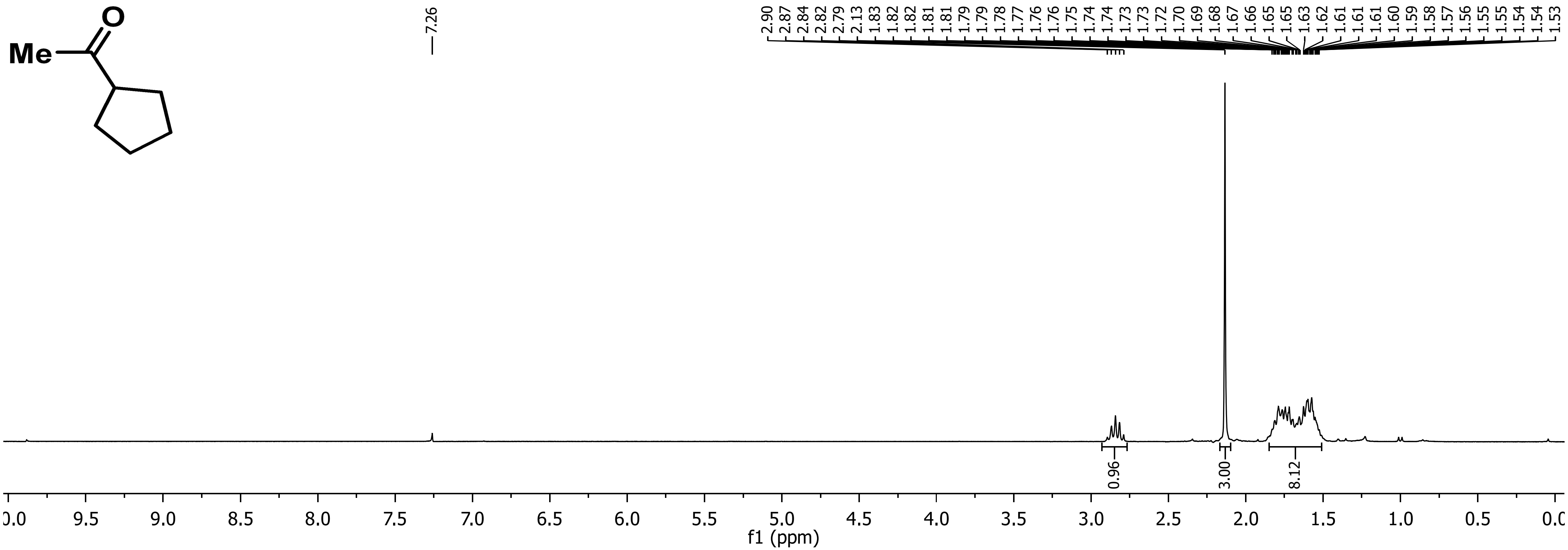
Me
O
7.26
2.90 2.87 2.84 2.82 2.79 2.13 1.83 1.82 1.82 1.81 1.81 1.79 1.79 1.78 1.77 1.76 1.76 1.75 1.74 1.74 1.73 1.73 1.72 1.70 1.69 1.68 1.67 1.66 1.65 1.65 1.63 1.62 1.61 1.61 1.61 1.60 1.59 1.58 1.57 1.56 1.55 1.55 1.54 1.54 1.53
0.96
3.00
8.12
9.5 9.0 8.5 8.0 7.5 7.0 6.5 6.0 5.5 5.0 4.5 4.0 3.5 3.0 2.5 2.0 1.5 1.0 0.5
f1 (ppm)

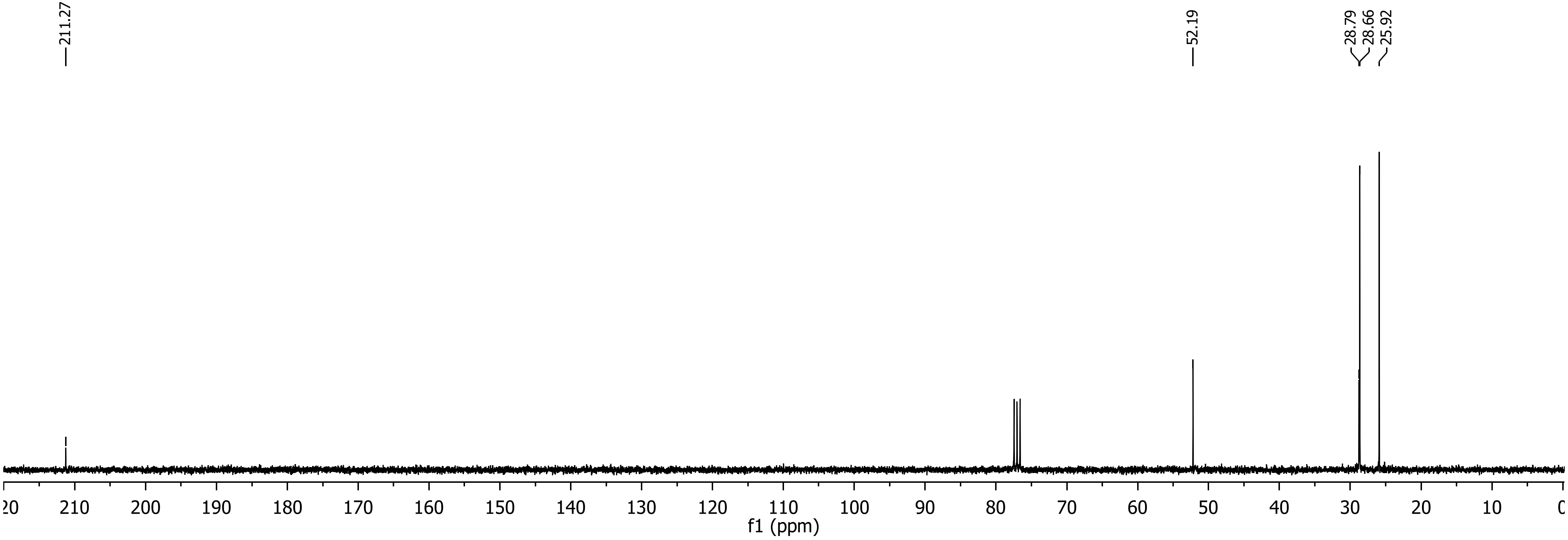
211.27
52.19
28.79
28.66
25.92
210 200 190 180 170 160 150 140 130 120 110 100 90 80 70 60 50 40 30 20 10
f1 (ppm)

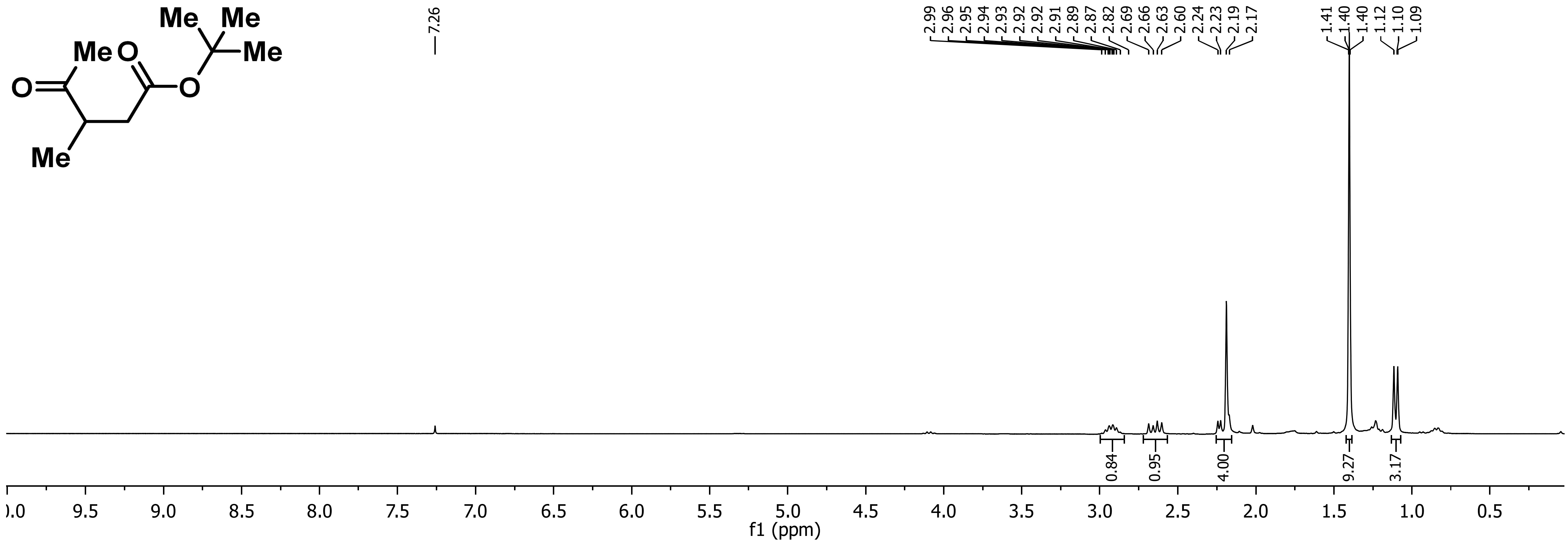
Me
Me
Me
Me
O
O
O
Me
7.26
2.99 2.96 2.95 2.94 2.93 2.92 2.92 2.91 2.89 2.87 2.82 2.69 2.66 2.63 2.60 2.24 2.23 2.19 2.17
1.41 1.40 1.40 1.12 1.10 1.09
0.84
0.95
4.00
9.27
3.17
f1 (ppm)

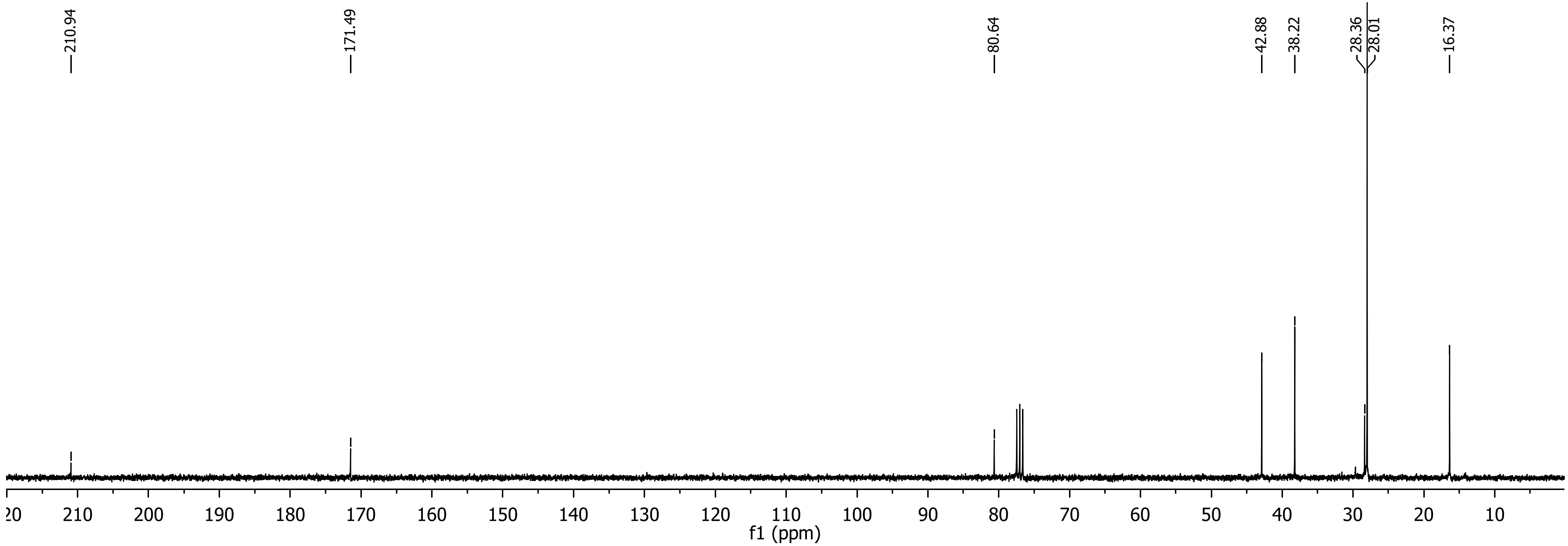
210.94
171.49
80.64
42.88
38.22
28.36
28.01
16.37
f1 (ppm)

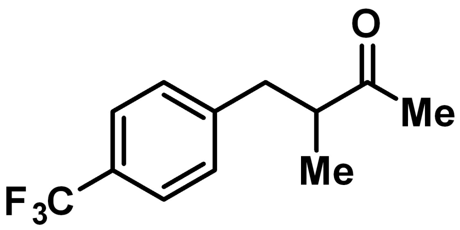
O
Me
Me
F3C

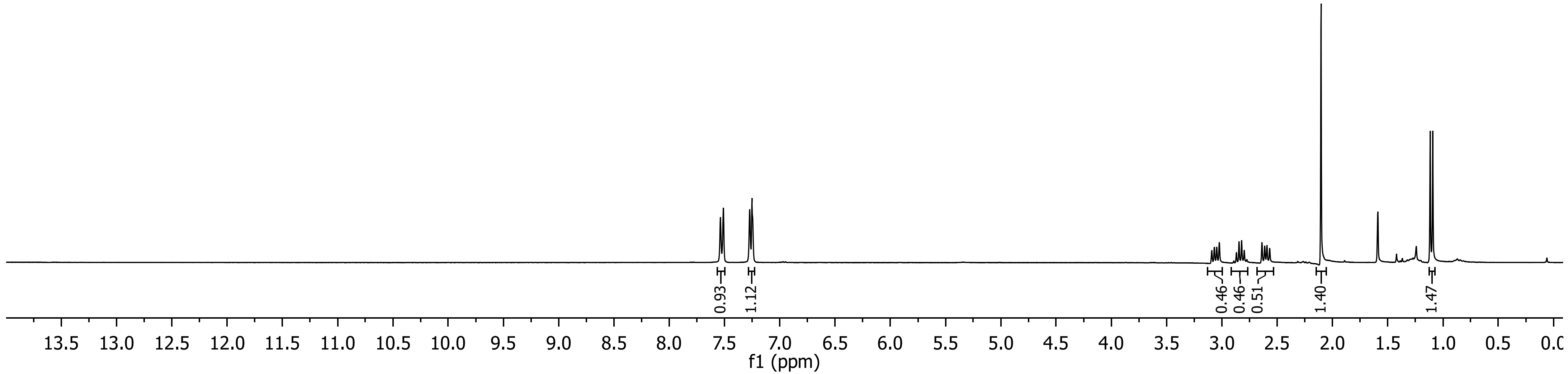
7.54
7.51
7.27
7.25
7.24
3.09
3.07
3.05
3.02
2.87
2.84
2.82
2.80
2.64
2.61
2.59
2.57
2.10
1.12
1.09
0.93
1.12
0.46
0.46
0.51
1.40
1.47
13.5 13.0 12.5 12.0 11.5 11.0 10.5 10.0 9.5 9.0 8.5 8.0 7.5 7.0 6.5 6.0 5.5 5.0 4.5 4.0 3.5 3.0 2.5 2.0 1.5 1.0 0.5
f1 (ppm)

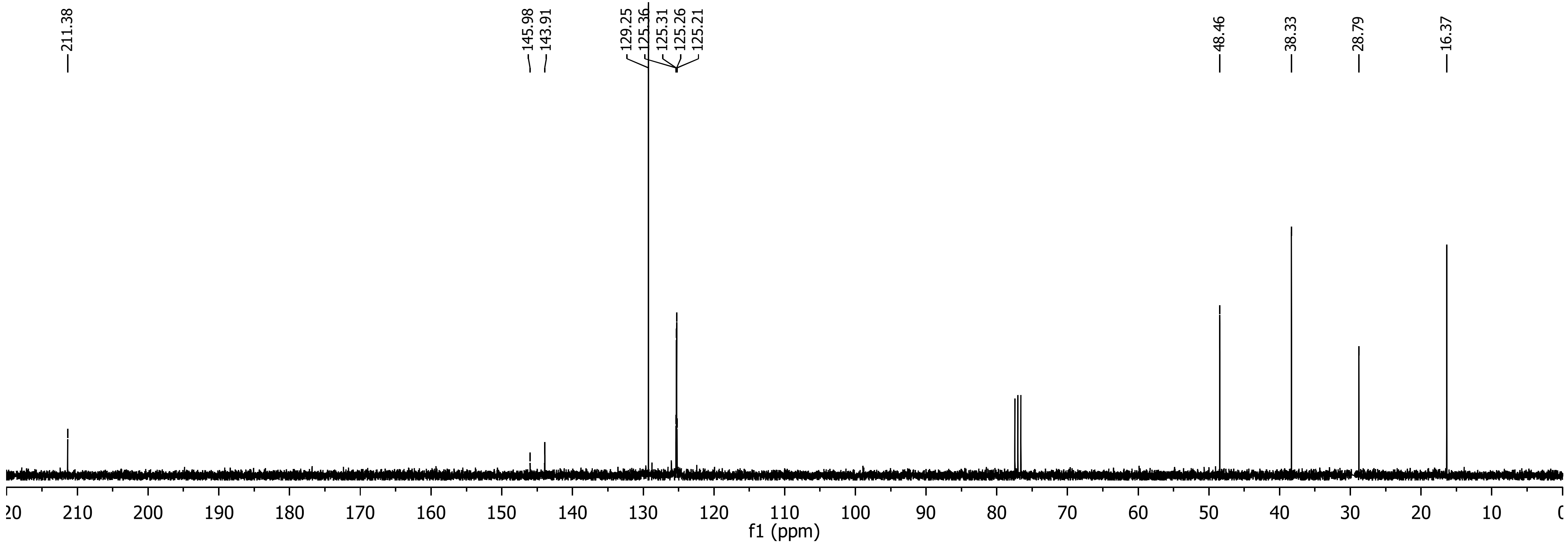
211.38
145.98
143.91
129.25
125.36
125.31
125.26
125.21
48.46
38.33
28.79
16.37
210 200 190 180 170 160 150 140 130 120 110 100 90 80 70 60 50 40 30 20 10
f1 (ppm)

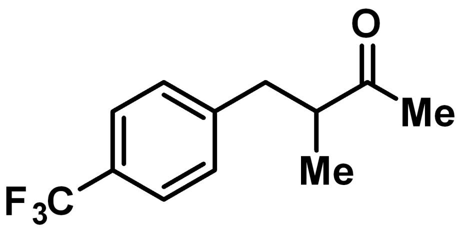
O
Me
Me
F3C

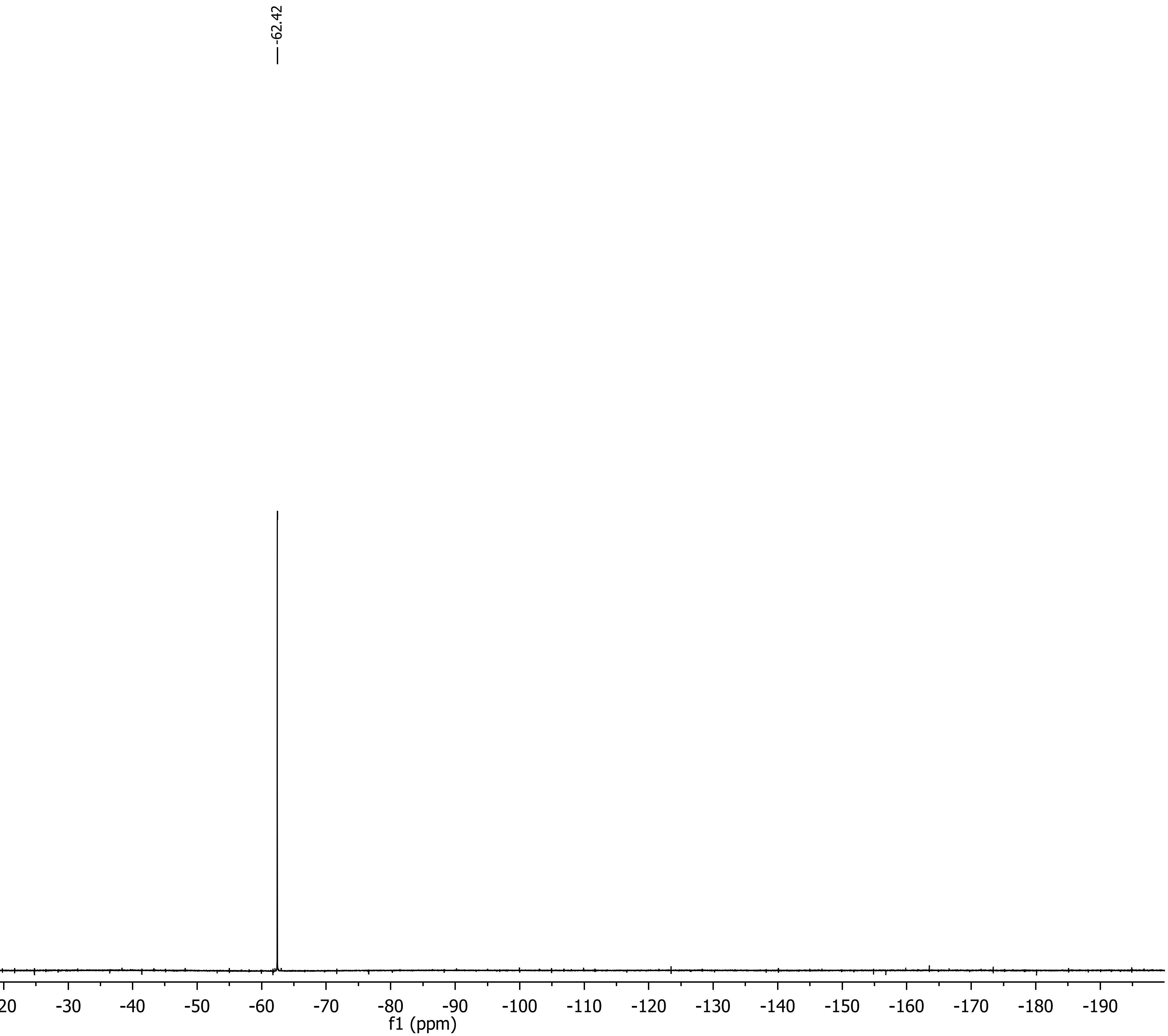
-62.42
20
10
0
-10
-20
-30
-40
-50
-60
-70
-80
-90
-100
-110
-120
-130
-140
-150
-160
-170
-180
-190
f1 (ppm)

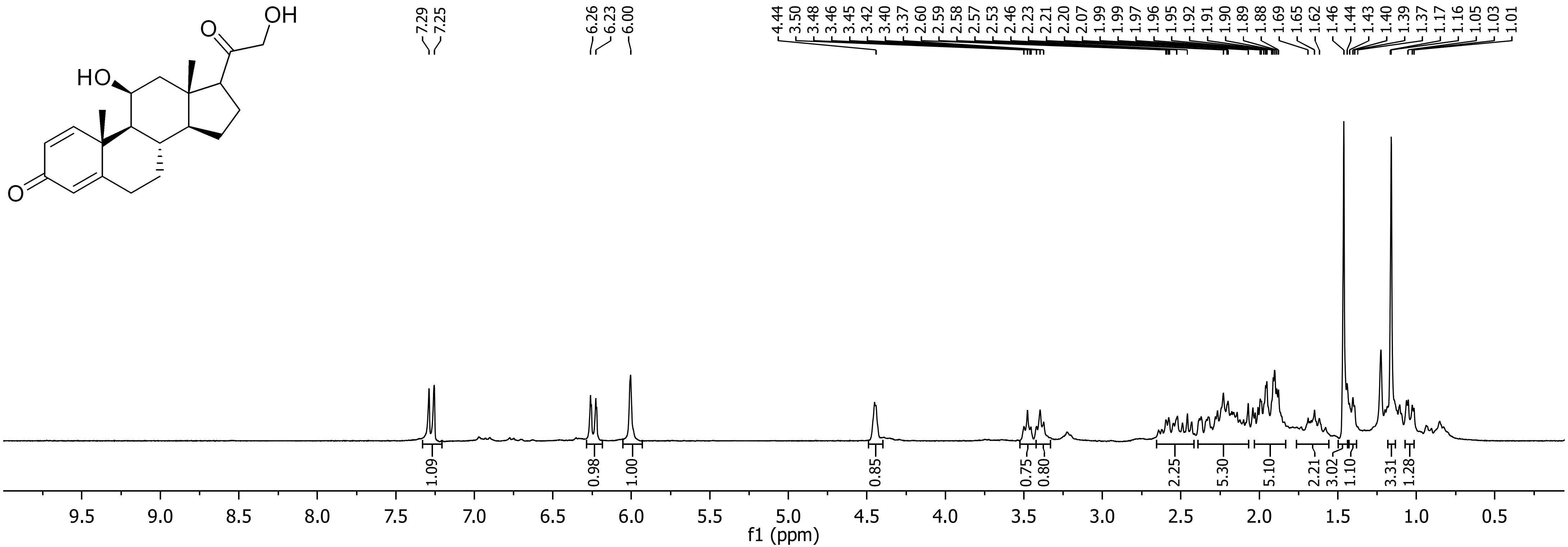
OH
O
HO
O
7.29
7.25
6.26
6.23
6.00
4.44
3.50
3.48
3.46
3.45
3.42
3.40
3.37
2.60
2.59
2.58
2.57
2.53
2.46
2.23
2.21
2.20
2.07
1.99
1.99
1.97
1.96
1.95
1.92
1.91
1.90
1.89
1.88
1.69
1.65
1.62
1.46
1.44
1.43
1.40
1.39
1.37
1.17
1.16
1.05
1.03
1.01
1.09
0.98
1.00
0.85
0.75
0.80
2.25
5.30
5.10
2.21
3.02
1.10
3.31
1.28
9.5
9.0
8.5
8.0
7.5
7.0
6.5
6.0
5.5
5.0
4.5
4.0
3.5
3.0
2.5
2.0
1.5
1.0
0.5
f1 (ppm)

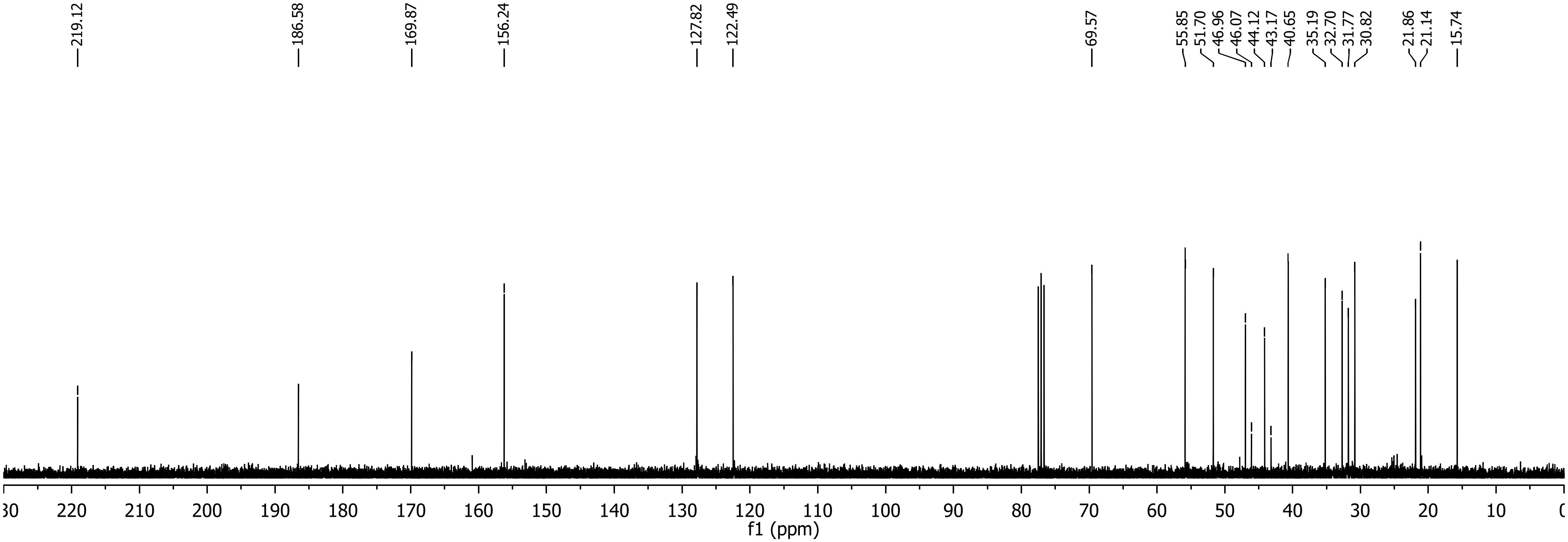
219.12
186.58
169.87
156.24
127.82
122.49
69.57
55.85
51.70
46.96
46.07
44.12
43.17
40.65
35.19
32.70
31.77
30.82
21.86
21.14
15.74
220
210
200
190
180
170
160
150
140
130
120
110
100
90
80
70
60
50
40
30
20
10
f1 (ppm)

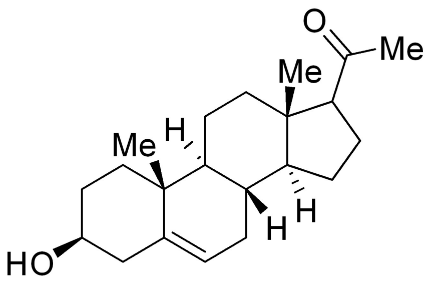
O
Me
Me
H
Me
H
H
HO

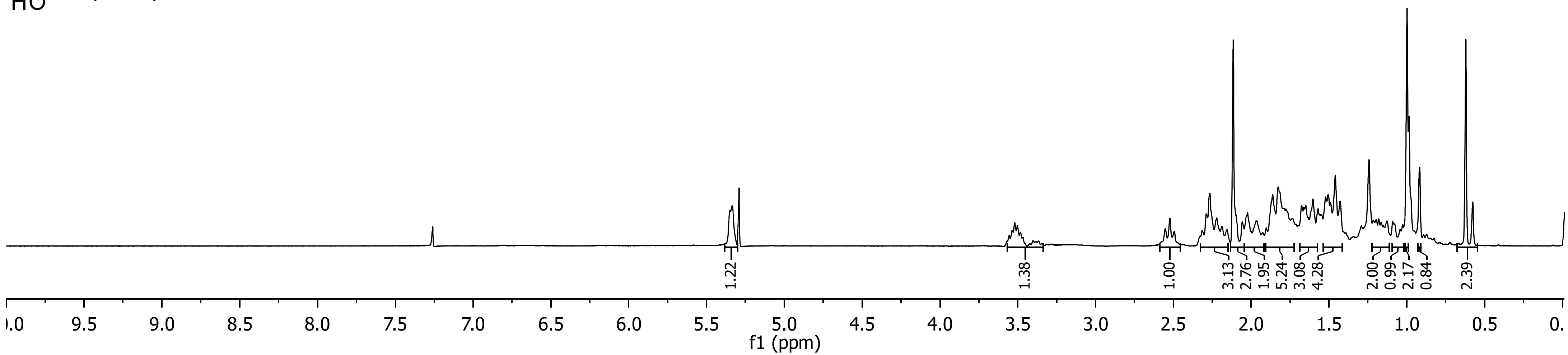
1.22
1.38
1.00
3.13
2.76
1.95
5.24
3.08
4.28
2.00
0.99
2.17
0.84
2.39
9.5 9.0 8.5 8.0 7.5 7.0 6.5 6.0 5.5 5.0 4.5 4.0 3.5 3.0 2.5 2.0 1.5 1.0 0.5
f1 (ppm)

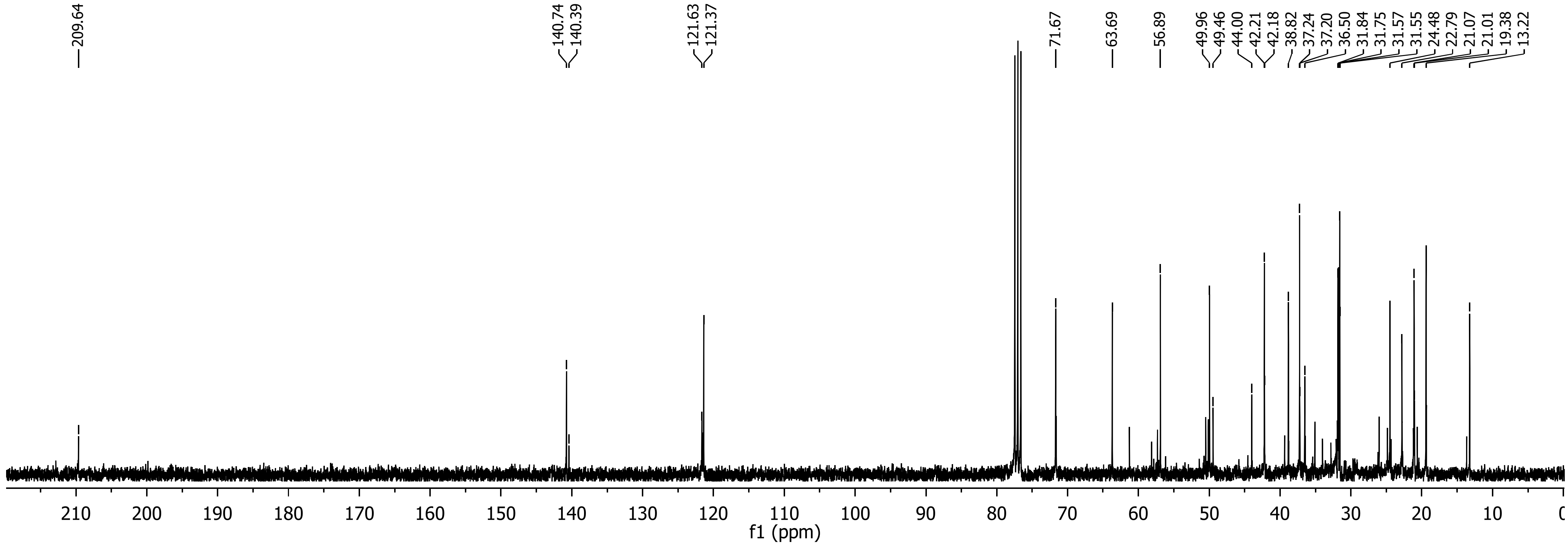
209.64
140.74
140.39
121.63
121.37
71.67
63.69
56.89
49.96
49.46
44.00
42.21
42.18
38.82
37.24
37.20
36.50
31.84
31.75
31.57
31.55
24.48
22.79
21.07
21.01
19.38
13.22
210 200 190 180 170 160 150 140 130 120 110 100 90 80 70 60 50 40 30 20 10
f1 (ppm)
